# Development and evaluation of a scoring system for assessing incisions in laser surgery

**DOI:** 10.1038/s41598-022-18969-0

**Published:** 2022-08-30

**Authors:** Martin Hohmann, David Kühn, Moritz Späth, Max Rohde, Florian Stelzle, Florian Klämpfl, Michael Schmidt

**Affiliations:** 1 Institute of Photonic Technologies (LPT), Friedrich-Alexander-Universität Erlangen-Nürnberg (FAU), Konrad-Zuse-Straße 3/5, 91052 Erlangen, Germany; 2 Erlangen Graduate School in Advanced Optical Technologies (SAOT), Paul-Gordan-Straße 6, 91052 Erlangen, Germany; 3 Department of Oral and Maxillofacial Surgery, University Hospital Erlangen, Ulmenweg 18, 91054 Erlangen, Germany

**Keywords:** Applied optics, Optical sensors, Biomedical engineering, Data acquisition, Medical research

## Abstract

The idea of laser surgery is nearly as old as the laser itself. From the first trials to modern laser surgery systems, it was and is the aim to selectively cut the tissue in the focus spot without causing harm to surrounding structures. This is only possible when the correct parameters for the surgical laser are chosen. Usually, this is done by parameter studies. However, the concrete evaluation scheme often differs between groups and more precise approaches require staining and microscopic evaluation. To overcome these issues, a macroscopic scoring system is presented and evaluated. It can be shown that the scoring system works well and, thus, a laser cut can be evaluated within a few seconds. At the same time, the whole cutting front is taken into account. The presented scoring system is evaluated by the intra class correlation (ICC). The final agreement between different raters is more than 0.7. Therefore, the scoring system can be used to optimize and evaluate the cutting process and it should be suitable for comparing the results between different groups. Definitely, it can be applied for scoring within a group to enable e.g., a profound statistical analysis for a parameter study.

## Introduction

It is known that laser surgery has developed to be a general accepted tool in various surgical areas^1^ as the use of lasers in hospitals is increasing^2^. For laser surgery, lasers provide comparable results as conventional surgery while enabling minimal invasiveness^3,4^. There are many other advantages such as great healing potential, less postoperative inflammation and swelling^5^ and the concomitant coagulation of small blood vessels allows for a dry operating field and better visibility^6^. In addition to the classical laser surgery, new technologies emerge which rely on local heating of the tissue such as monopolar coagulation or plasma beam coagulation^7^.

Despite the fact that laser surgery and other modalities provide a lot of advantages, no haptic feedback is provided compared to the conventional work with surgical instruments. Thus, the risk of tissue damage to vital structures lies within any non-contact device. Hence, it is of utmost importance for any surgical laser application to know the tissue damage (e.g. depth of damage, different damage zones, reversible damage versus irreversible damage). Especially for surgical interventions in the direct vicinity of sensible anatomical structures which have to be preserved (nerves, major blood vessels, salivery ducts, urinary ducts,...) exact parameters of laser damage to the tissue is absolutely necessary to provide minimal damage to the patient.

For better understanding of the thermal damage, the heat distribution should be considered. In general for laser surgery, the energy from the laser is absorbed, resulting in increasing temperature of the tissue. The increasing temperature leads to the desired ablation of the material. The heat transport, however, causes the unwanted side effect of damaging the surrounding. For this, Lévesque et al.^8^ investigated different heat transport models on bone between 20 and 320 °C. Heat conduction, heat convection and heat radiation all take place. Already beginning from 125 °C, the heat radiation dominates^8^ with a dependence on $T^4$. Thus, a small temperature increase leads to a large increase of the amount of transported heat. For low temperatures in the range from 20 to 50 °C, heat conduction dominates^8,9^. Based on the reached temperature in the surrounding, denaturation, carbonisation and thermo-mechanical ablation might dominate. This effect was already shown by McKenzie^10^.

Nevertheless, not only the understanding of the cause of the damage is essential, also the evaluation of the damage is of great importance for practical applications. Originally, hematoxylin–eosin (HE)-staining is used to assess the thermal damage^11^. Later, Goertz^12^ evaluated different histological stainings for denaturation. It could be shown that the Hinshaw-Pearse-staining allows to visualize thermal influences. Later, Vescovi et al.^13^ used a point scale for histological samples. Their scoring is based on the morphology of the incision as well as the alteration of vessels and cell structures; for staining, standard HE-staining is used. Magdy et al.^14^ compared the effectiveness and damage of dissection-ligation, monopolar electrocautery and laser tonsillectomies. For damage assessment, HE-staining is used where thermally damaged areas show a dark colour. Cercadillo-Ibarguren et al.^15^ had the most elegant solution by adding Masson-Trichromat-staining for masking of false positives by the HE-staining. Cercadillo-Ibarguren et al.^15^ also measured the thickness of the thermally damaged tissue and used this as quantification for comparison of different methods.

Separate from the method from Cercadillo-Ibarguren et al.^15^, there is no means of reliable quantification of the damage by laser surgery known to the authors so far. While the methods from Cercadillo-Ibarguren et al.^15^ and Vescovi et al.^13^ are already a huge advancement, they have major drawbacks: first, as statistically reliable results should be generated, a large amount of measurements is required. Due to the fact that the process of staining and microscopic analysis takes a lot of time, this is not an optimal solution. Second, microscopic approaches can only analyse a small section of the cut under evaluation. It is likely that this is not a reliable representation of the cut done by the laser or other tools and, thus, it is not optimally suited for statistical evaluation.

Therefore, this study proposes a macroscopic scoring system is proposed which overcomes these issues: a scoring system is used to classify clinical pictures or injury patterns as well as to make diagnoses and to be able to describe different patient conditions in a uniform nomenclature. The scoring system can be conducted within a few seconds by a trained scientist, which is comparably fast and an advantage for the practical application. In comparison, HE-staining consists of several steps that incluce cryosectioning of specimen, tissue preparation, and the staining process itself. While the staining time alone is limited to a few minutes of reagent exposure, the entire process of tissue preparation, staining and preparation of specimen for microscopic evaluation takes several hours. Thus, many samples can be rated by the proposed scoring system easily within the time required for HE-staining. Another advantage is that the entire cutting front is taken into account so that the scoring represents the quality of the overall cut.

The requirement for a good scoring system is its reliability or, in other words, the fact that different raters give the same or at least similar scores. As this part is the most essential part, this evaluation is the main part of this study. To even generalize the result more, the scores from trained and untrained scientists are evaluated and compared. The agreement between the raters is evaluated by intra class correlation (ICC)^16^ as the state of the art statistical evaluation technique for rater agreement and consistency.

## Materials and methods

The “Materials and methods” section consists of two parts. In the first part, the scoring system is presented and explained. In the second part, the methods for the evaluation of the scoring system are shown. All cuts are done with a CO$_2$-laser with varying parameters on muscle tissue from freshly slaughtered pigs bought from a local butcher. Those parameters are not explained in detail as many parameters were varied and, at the same time, the laser parameters are not relevant for this study. The parameter study will be done in a follow-up study with the help of the presented scoring system.

The central goal of the scoring system presented in this study is to be fast to allow the evaluation of many cuts for a reliable statistical interpretation of the cuts. With the help of the ICC, it can be evaluated if the scoring system provides these reliable results. This is done by evaluating the scoring of the same samples by different raters.

### Scoring system

**Figure 1:**
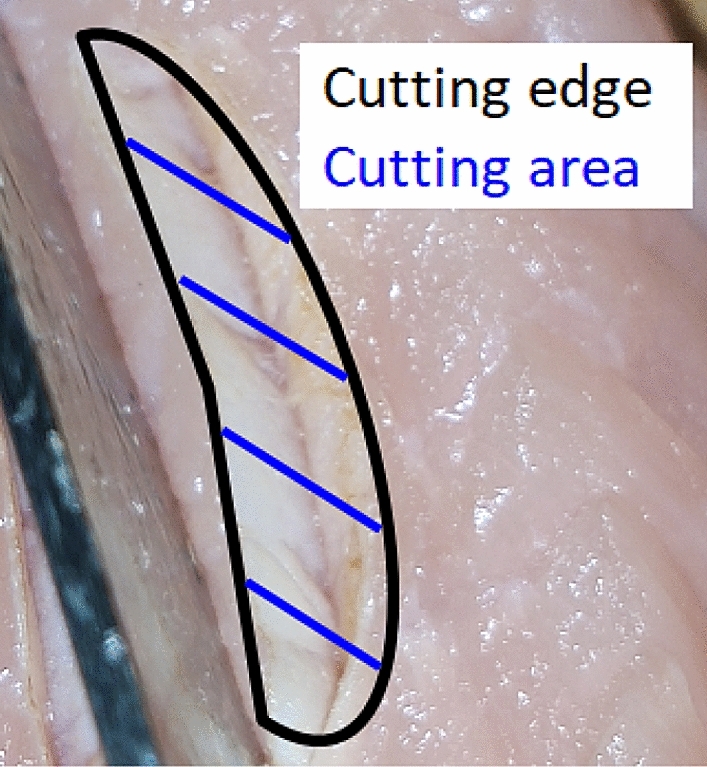
Exemplary cut without much tissue damage. The two methods of scoring are the cutting edge (CE); blue scoring of the cutting area (CA).

When a standard cut is taken into account, there are two potential visible parts which can be used for scoring: the cutting area (CA) and the cutting edge (CE). Both of these parts are shown in Fig. 1. The CA is defined as the area of the new surface produced by the cutting action which is marked by the blue hatching lines in Fig. 1. The CE is defined as the fine rim between the cut and the uncut surface of the tissue. This is marked by the black line in Fig. 1. The differentiation between CA and CE is done for the following reasons: most of the potential damage occurs on the CA. Hence, it should contain more information about the damage by the cut. It might, however, not be accessible in practice as opening the cut might lead to further damage. The CE is always accessible but it can only transfer indirect information about the cutting area by the interaction of the hot fumes and the ablated material from the ablation process. It is known from the field of laser material processing that the ablated material carries relevant information about the interaction process of the laser with the substrate^17^. Therefore, it is expected that the CE can be used for a reliable evaluation of the laser surgery process. In summary, the CA is expected to have a more precise and easier evaluation compared to the CE; its evaluation, however, might not always be possible.

There are two possible features which can be used for the evaluation of the scoring of the laser surgery process (in terms of CA and CE): the colour of the tissue and the covered area of discoloured tissue. There are basically five tissue colours which might appear: black, dark brown, light brown, white and pinkish. The colours black, dark brown and light brown represent the degree of carbonization of the tissue. The colour white pinpoints to coagulation effects, while pinkish represents mainly undamaged tissue. These different colours would lead already to a possible scoring system. To add additional information, the amount of area which is damaged is taken into account.

Different ranges of colour darkening can be defined. In this study, the following ranges are chosen: 80%+, 50%+, 30%+, below 30% and no or nearly no darkening of the tissue. These numbers are chosen as they allow optimization of the parameter studies towards high quality cuts. As mentioned before, the tissue colour in laser surgery can also be roughly grouped into five categories (black, dark brown, light brown, white, pinkish/natural colour). Thus, it makes sense that the scoring system comprises five rating variables. As a single colour does not appear isolated, each score should span a range of discolourations. In other words: a statistical analysis only makes sense if the difference between complete carbonization or partial carbonization can be distinguished. By the different amounts of brown/black tissue and by their colour, the carbonization can be evaluated.

**Figure 2:**
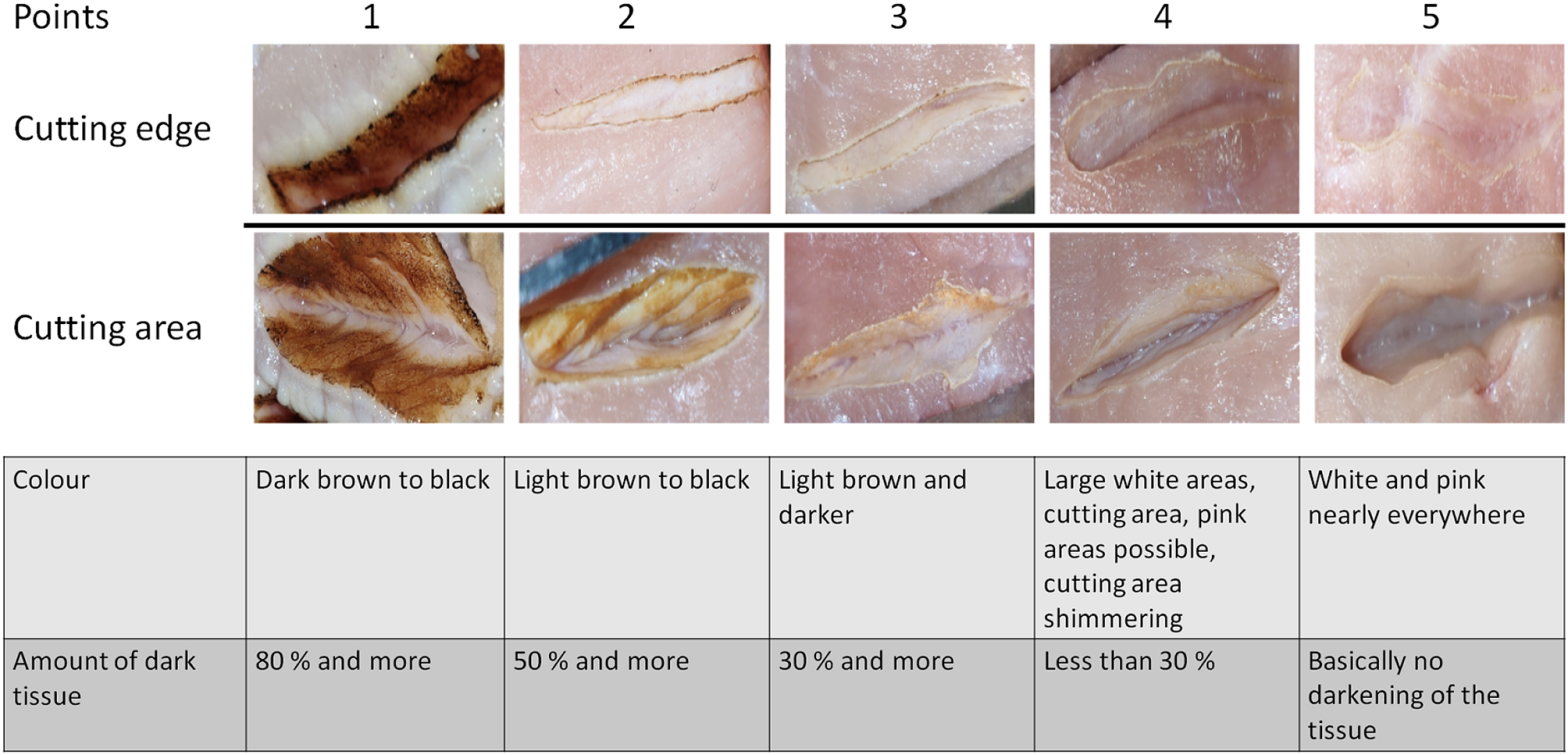
Example of the scoring system: the columns represent the points in the scoring system while the rows represent the scoring for the cutting edge (CE) and the area inside the cut (cutting area), respectively.

The final scoring parameters are shown in Fig. 2. The scoring system is set to range from 1 point to 5 points where 5 is the least darkening (which is considered the best):

1. The results show strong carbonization. The tissue is darkened nearly everywhere (80%+) and the colour is mostly dark brown to black.
2. The carbonization is still there but less than for the previous score. More than half of the tissue (50%+) is darkened. In contrast to score ’1’, light brown tissue colouring is present. Sometimes black areas might still appear.
3. The carbonization is light. Normally, no black tissue should appear. Typically, carbonization leads to light brown and dark brown tissue colouring. In total, around 30–50% of the tissue are darkened.
4. Less than 30% of the tissue is darkened. Pink areas might appear and typically only light brown tissue should appear. For ex-vivo experiments, shimmering is possible which is caused from present water. Normally, a large part of the tissue is white due to coagulation.
5. There is nearly no tissue damage except the cut itself. There should be no or nearly no darkening of the tissue. The dominating tissue colours are pinkish and white.

### Evaluation of the scoring system

#### Background

For the scoring system, a few general thoughts have to be considered. First, there is currently no gold standard for the evaluation of laser surgery to which the scoring system can be compared. This is a similar problem scientists face in the field of psychology as there is no objective gold standard for many psychological diseases. Despite this, a scoring system would be helpful for these diseases. This problem can be overcome by assessing how similar independent people rate the same patient. In the case of this study, it is checked how different people rate the same laser surgery cuts independently from each other. For the analysis of a new scoring system, the ICC is the state of the art to analyse the inter-rater reliability^18^. In a simplified explanation, the ICC checks how similar different raters rate each sample. If the different raters rate similar, the ICC will be high and the scoring system performs well. Moreover, the results from the ICC can be compared to a correlation as the correct rating is not known a priori.

#### Scoring procedure

**Figure 3:**
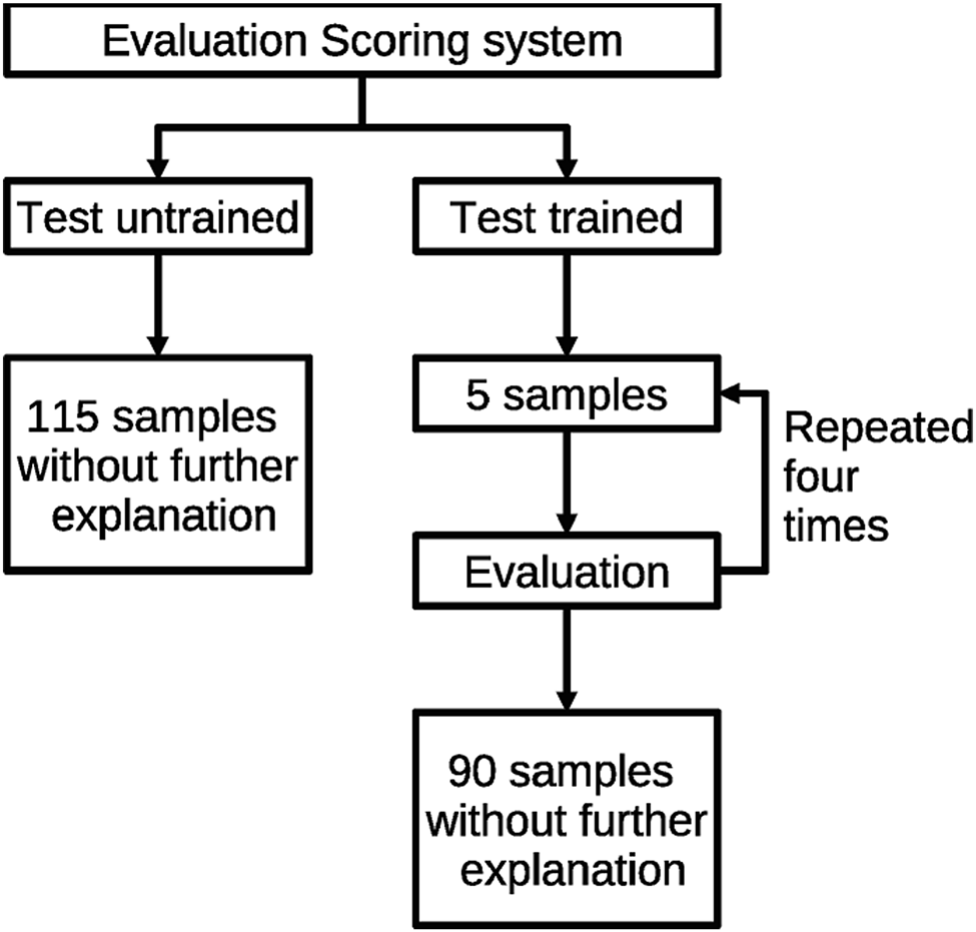
Evaluation of the scoring system. In the first setting, the scoring is done for untrained raters (left) and in the second setting for trained raters (right).

For the evaluation of the scoring system, two different settings are studied as shown in Fig. 3: first, a group of six scientists is asked to evaluate 115 cuts with just the explanation shown in Fig. 2. All images used for the analysis in this study are available in the “Supplementary materials”. This experiment should show that the scoring system is already reliable for untrained scientists and, therefore, is suited for the evaluation of the laser surgery cuts. Second, a group of six different scientists is asked to do the same evaluation again. In this part, the additional information in Fig. 1 is provided and the first 25 examples are used as a training session with regular feedback. For this, the raters are asked to rate the first five cuts. Afterwards, the solution according to the first author of the present study is presented and discussed. This is repeated for the next 20 cuts with the same procedure as shown in Fig. 3 on the right path. It should be noted that the important part of the training period is the discussion in which questions by the raters arise. The order of the pictures for evaluation was slightly changed for the test of the trained raters. This was done to ensure that from all cutting qualities at least 3 examples are present in the first 25 samples.

In total, all 12 raters are scientists in the fields of optics. None of them has experience in the field of laser surgery. Only two of the scientists have experience in biophotonics. Both of these scientists are in the untrained raters group. The rest of the scientists works in the field of laser material processing with at least one of the following fields of expertise: Welding/Cutting with lasers, additive manufacturing with metals, simulation of laser material processing and sensing for laser material processing.

#### Statistical evaluation

The agreement of the raters is evaluated by ICC^16^ in Python with the pingouin framework^19^ with the command “intraclass-corr”. The ICC is a descriptive statistics that can be used when units (the quality of the cut) are organized in groups of quantitative measurements (scores). It is seen as framework of random effects models. The ICC describes how closely units in the same group resemble each other. It is used to quantify the degree to which different observers (in this study: raters) are consistent or reproduce the same results. As a reminder, the ICC can be seen as a more sophisticated version of a correlation such as Pearson’s correlation. While typical correlations only correlate pairwise, the ICC correlates all scores at once. This leads to two conclusions: The ICC is to be preferred and it can be interpreted similar to a standard correlation coefficient. The ICC ranges from zero to one and the higher the ICC, the better is the scoring system. According to Koo et al.^18^, an ICC between 0.50 and 0.75 is moderate, between 0.75 and 0.90 is good and higher numbers mean excellent. Other authors speak about a high agreement when the ICC is larger than 0.7^20^. In general, the range of an optimal result for the ICC is still under discussion.

The untrained as well as the trained raters are evaluated by the ICC for the whole data for the CE as well as the CA. For the CA, a more elevated analysis is done by using the ICC for the first and last third of the scored cuts. Through this information, the effect of learning experience on the scoring system can be gathered. The effect of training is measured by comparing the results of the ICC from the first third and from the last third of the scored samples. For the ICC, six values are calculated^19^:

- **ICC1:** Each target is rated by a different rater. The raters are selected at random. For the calculation a one-way Anova fixed effects model is used. The ICC1 is sensitive to differences in the means between raters.
- **ICC2:** A random sample is rated by k raters and the absolute agreement is measured. The ICC2 is sensitive to interactions. The raters are selected randomly.
- **ICC3:** A fixed set of k raters rate each target. Hence, there is no generalization to a larger population of raters. The ICC3 is sensitive to interactions. The raters are selected e.g. due to abilities or special prior training.
- **ICC1k:** Same as ICC1 however, the reliability is estimated for the mean of k raters. The results are similar to the Spearman Brown adjusted reliability or Cornbach’s Alpha.
- **ICC2k:** Same as ICC2 however, the reliability is estimated for the mean of k raters. The results are similar to the Spearman Brown adjusted reliability or Cornbach’s Alpha.
- **ICC3k:** Same as ICC3 however, the reliability is estimated for the mean of k raters. The results are similar to the Spearman Brown adjusted reliability or Cornbach’s Alpha.

From this, it can be concluded that ICC3 has to be analysed in this study as fixed raters (which are scientist in the fields of optics) are chosen as raters. There are two different possible tests which can be done: the consistency or relative agreement versus the absolute agreement. While the latter describes how similar the raters do the rating, the first one describes the similarity of the tendency. This means if some raters rate the samples always lower, the consistency will be high. However, the agreement will be low.

As the model should test how good the results can be compared with different groups, the absolute agreement is calculated. It should be noted that the absolute agreement is always lower or equal to the consistency. Hence, if the absolute agreement is high, the relative agreement (consistency) will be even higher. Thus, the proposed scoring system is suited for parameter studies as well.

Additionally, the Spearman’s correlation is calculated as more readers might be familiar with it. It measures the linear correlation between two raters. However, as only a pairwise correlation can be done, the ICC is preferred. Thus as a result, the averaged Spearman’s correlation is presented for CA and CE. This should provide similar values as the ICC. Furthermore, the averaged Spearman’s correlation between CA and CE is presented to show the similarity between both of them and it is tested whether CE and CA show the same scores by means of the Wilcoxon signed-rank test. This test is chosen as the score is not Gaussian distributed and the samples are related (same sample).

## Results and discussions

In this section, the evaluation of the scoring method is presented.

### Evaluation scoring for untrained raters

Table 1 shows the results for untrained raters from the ICC for the CE and the CA, respectively. Additionally, “CI 95%” represents the confidence interval in which with 95% probability the final ICC is found. It can be seen that the ICC is higher for the CA. The feedback from the raters hints that scoring the CE is more difficult than scoring the CA. This is in agreement with the results from the CE and CA. Although for scoring, the CA is preferable for untrained raters, it is not always visible and available. In this case, the CE has to be used. However, the distribution of the results from the raters from the CA and the CE are significantly different ($p \ll 1 \times 10^{-3}$), meaning that CE and CA test something different. Nevertheless, both lead to similar results for good cuts, so both are valid. The Spearman’s correlation shows a similar trend. The average Spearman’s correlation between the CA and the CE is 0.41. Therefore, this also hints that CE and CA are partly independent parameters. The average Spearman’s correlation for CE and CA are 0.62 and 0.70, respectively. This results are comparable to the results from the ICC.

**Table 1:** Results ICC for the CE and CA for untrained raters.

Type	Description	ICC CE	CI 95% CE	ICC CA	CI 95% CA
ICC3	Single fixed raters	0.62	[0.54, 0.69]	0.71	[0.65, 0.77]

Table 2 shows the results of the ICC for the first and the last third of the CA. It can be clearly seen that there is a learning effect by the raters. The ICC increases by about 0.2. Thus, it is strongly recommended that new raters practice with one or two hundred samples before rating the final data set.

**Table 2:** ICC for the CE from the data from the first third and the last third of the scoring for untrained raters.

Type	Description	CA 1.	CI 95% CA 1	CA 3	CI 95% CA 3
ICC3	Single fixed raters	0.56	[0.43, 0.7]	0.75	[0.64, 0.84]

### Evaluation scoring for trained raters

Table 3 shows the results for trained raters from the ICC for the CE and the CA, respectively. It can be seen that the absolute agreement is higher for the CE than for the untrained raters in Table 1. The training seems to slightly increase the results for CE while the effect for CA is very small. It is expected that the small decrease with the CA might be caused by chance, by the effect that no scientist with experience in biophotonics did the rating with trained raters and/or by the alteration of the order of the pictures. To our view, the first explanation is the most likely one as the difference of the ICC is minimal. Again, the feedback from the raters hint that scoring the CE is more difficult than scoring the CA. In general, it can be said that the training is important especially for the CE.

**Table 3:** ICC for the CE and CA for trained raters.

Type	Description	ICC CE	CI 95% CE	ICC CA	CI 95% CA
ICC3	Single fixed raters	0.66	[0.58, 0.74]	0.69	[0.61, 0.76]

Table 4 shows the results of the ICC for the first and the last third of the CE for trained raters. The learning effect decreases compared to the untrained raters. However, it is still present. Hence, a long practice is required for more agreement between the different raters. Nevertheless, the training effect is lower in the case of trained raters.

**Table 4:** ICC for the CE from the data from the first third and the last third of the scoring for trained raters.

Type	Description	CA 1.	CI 95% CA 1.	CA 3.	CI 95% CA 3.
ICC3	Single fixed raters	0.64	[0.5, 0.78]	0.72	[0.59, 0.83]

### Limitations

In the first paragraph, the limitations of the evaluation of the scoring system are discussed. Afterwards, the limitations of the scoring system itself are presented and, in the last paragraph, the limitations of this study are shown.

There is currently one limitation of the evaluation of the scoring system. Due to the fact that many people should evaluate the scoring system, the evaluation was done on pictures of the cut instead of fresh cuts. However, this can also be turned into an advantage: It provides other researchers the opportunity to compare their own scoring results or even a modified scoring system to the one in this study as the pictures are available in the “Supplementary materials”.

A limitation of the presented scoring system is the transferability of the scoring results into clinical settings as only ex-vivo tissue from pigs were used. Due to this, effects such as perfusion are not regarded. In the current state, the clinical outcome of laser surgery cannot be predicted by the given scores; further studies are required to investigate if e.g. effects such as delayed wound healing or scar formation can be related to the scores. Furthermore, the fact that different tissue types such as skin or the vocal cord might tolerate a different amount of thermal damage is not considered yet. Nevertheless, this can be overcome easily: For sensitive tissue types, the worst score can be set to a lower amount of thermal damage. Finally, it should be highlighted that the usage of the presented scoring system allows at least ex-vivo a quick and efficient way for a parameter study for laser surgery. This will be shown in a follow-up study for cuts with a CO$_2$-laser.

The limitations of the current study are the following: First, all the evaluations were done on a single picture for each cut. Hence, parameters such as the angle or quality of the image influence the scoring. Second, none of the raters were experts in laser surgery. Thus, more work is needed to determine if a specific subclass of raters rate more consistently than another. Furthermore, the performance of the scoring system might also deviate if fresh tissue is evaluated directly after the cut. Despite these limitations, the scoring system still reaches an ICC of 0.71.

## Conclusion

It is possible to easily rate cuts done by a laser with the presented scoring system. CA and CE were evaluated. From the presented scoring systems, the one for the CA is preferable. As it cannot be accessed easily in all cases, the CE allows a similar use. According to the raters, however, the latter is more difficult to be rated, leading to a lower ICC. While the cuts were generated with a CO$_2$-laser in this study, there is no reason that the proposed scoring system cannot be applied to other laser systems or even other thermal cutting modalities.

Moreover, it is sure that the presented scoring is useful for optimizing the laser surgery parameters and might provide easy and quick results for their evaluation. The final ICC is 0.71. Hence, the presented scoring system should be a reliable, easy and fast system for the evaluation of cuts by means of laser surgery. The score of cuts can be compared between different groups. However, it should be evaluated by a second study with scoring fresh samples directly after the cut.

For the future, there is another study planned in which this scoring system is applied to fresh ex-vivo laser surgery of pig muscle tissue with a CO$_2$-laser. For this, the influence of the most important parameters on the results are studied and ranked by their importance.

## Supplementary Information

Supplementary Information.
