# Supplementary material for: Development and evaluation of a scoring system for assessing incisions in laser surgery
Source: Sci Rep. 2022 Aug 30;12:14741. doi: 10.1038/s41598-022-18969-0 (PMC9427958; doi:10.1038/s41598-022-18969-0)

Raw data for the study „Scoring for laser surgery“:

It contains the 115 images of laser cuts done with a CO_2 Laser. The images were presented in this way to the raters.

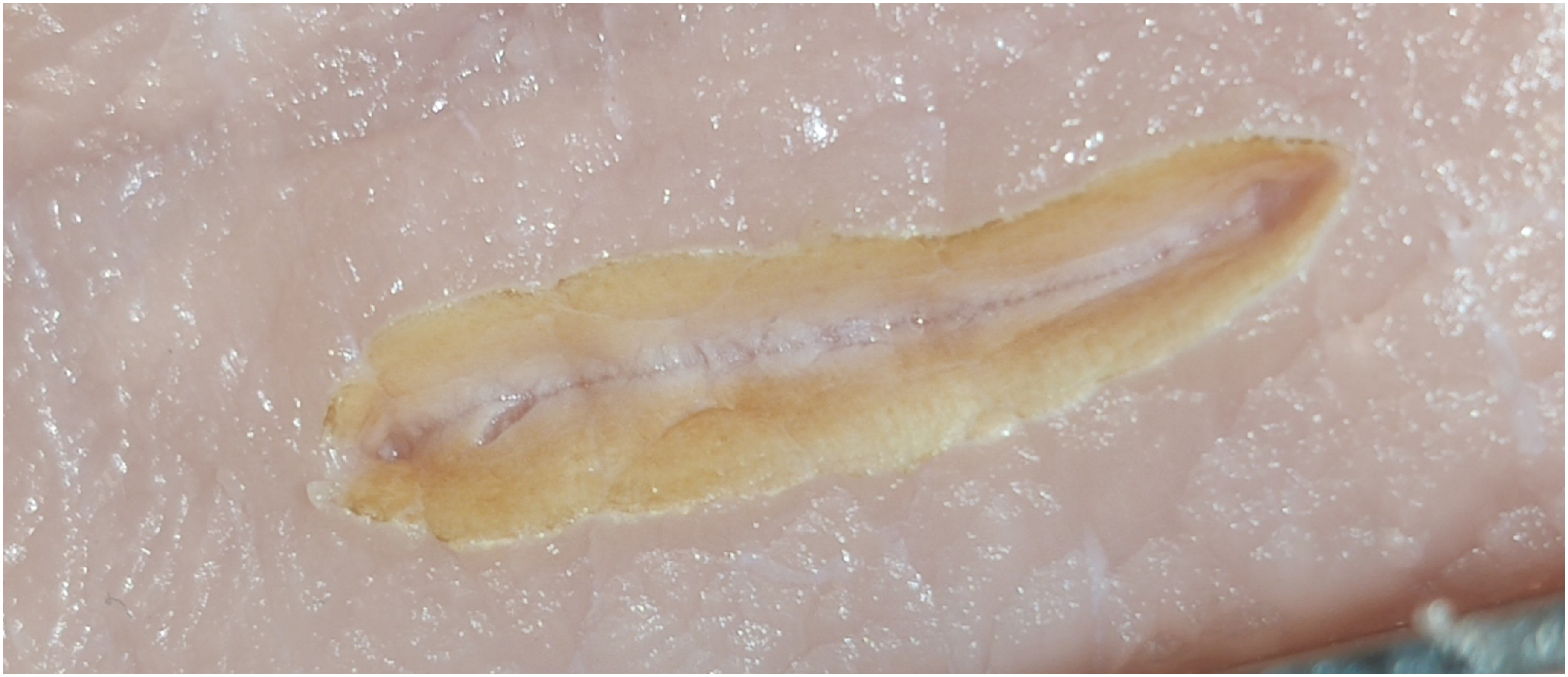

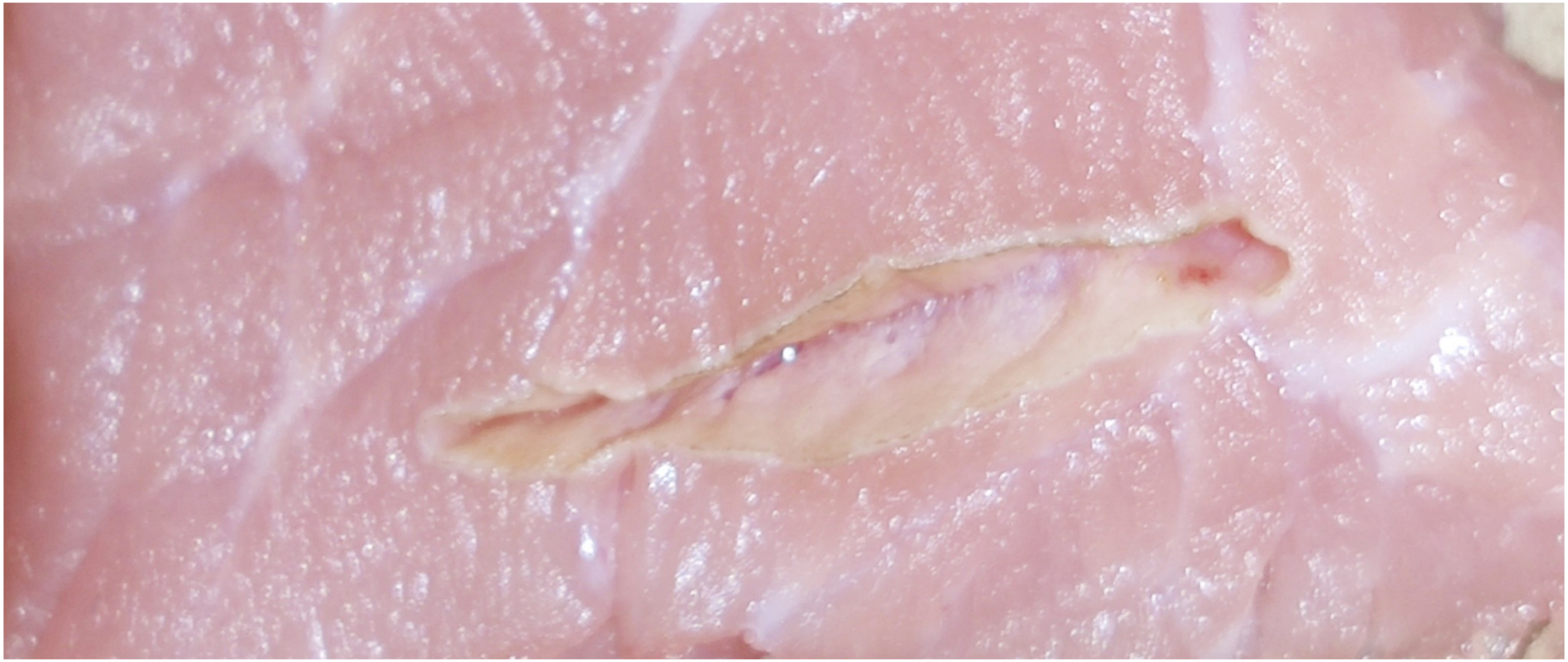

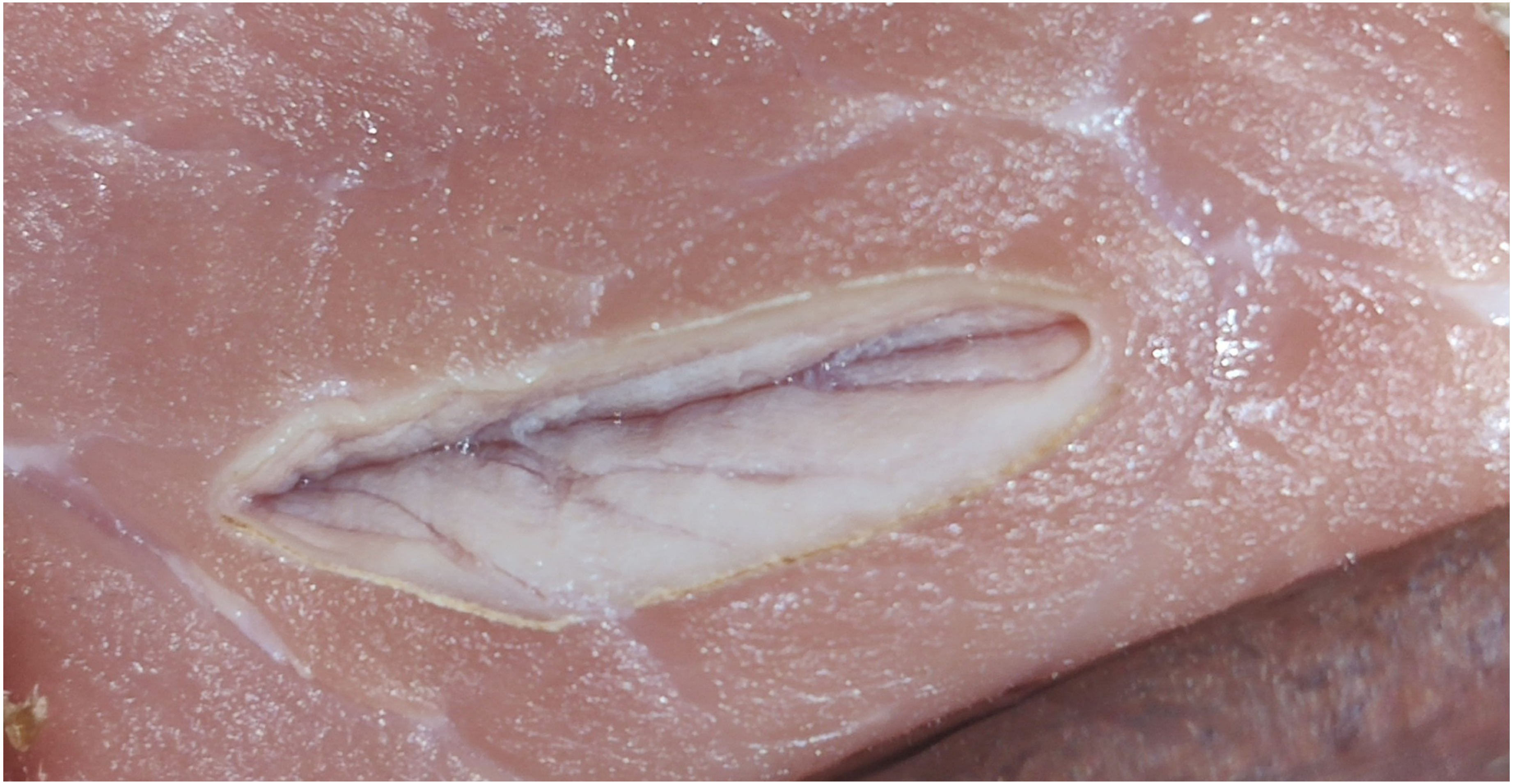

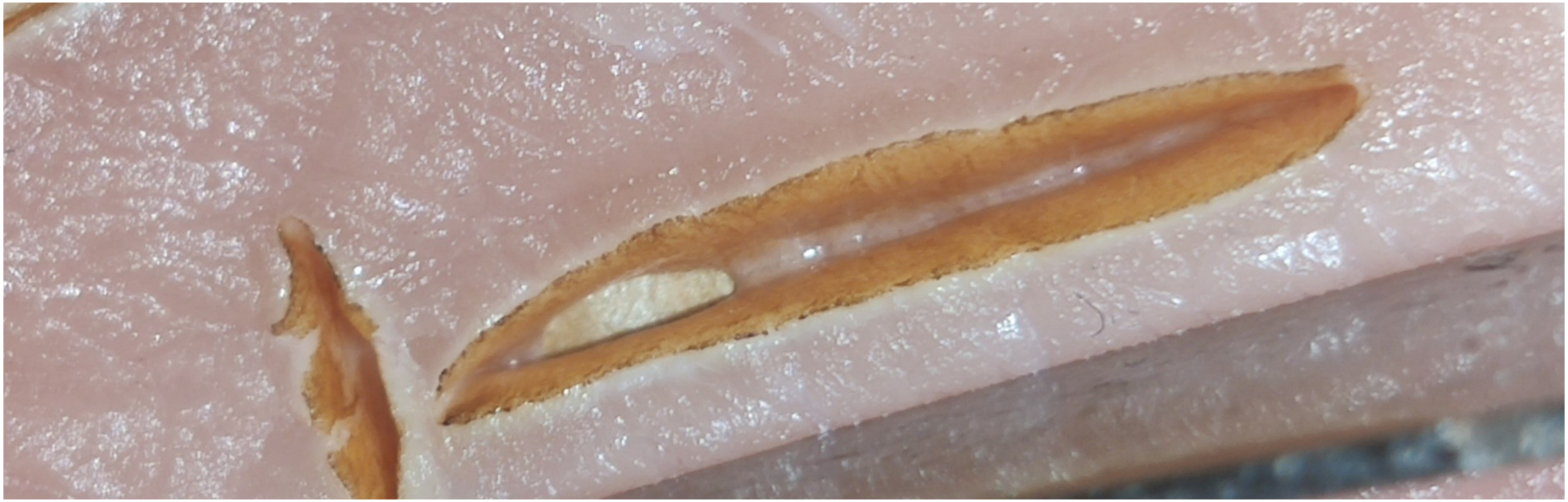

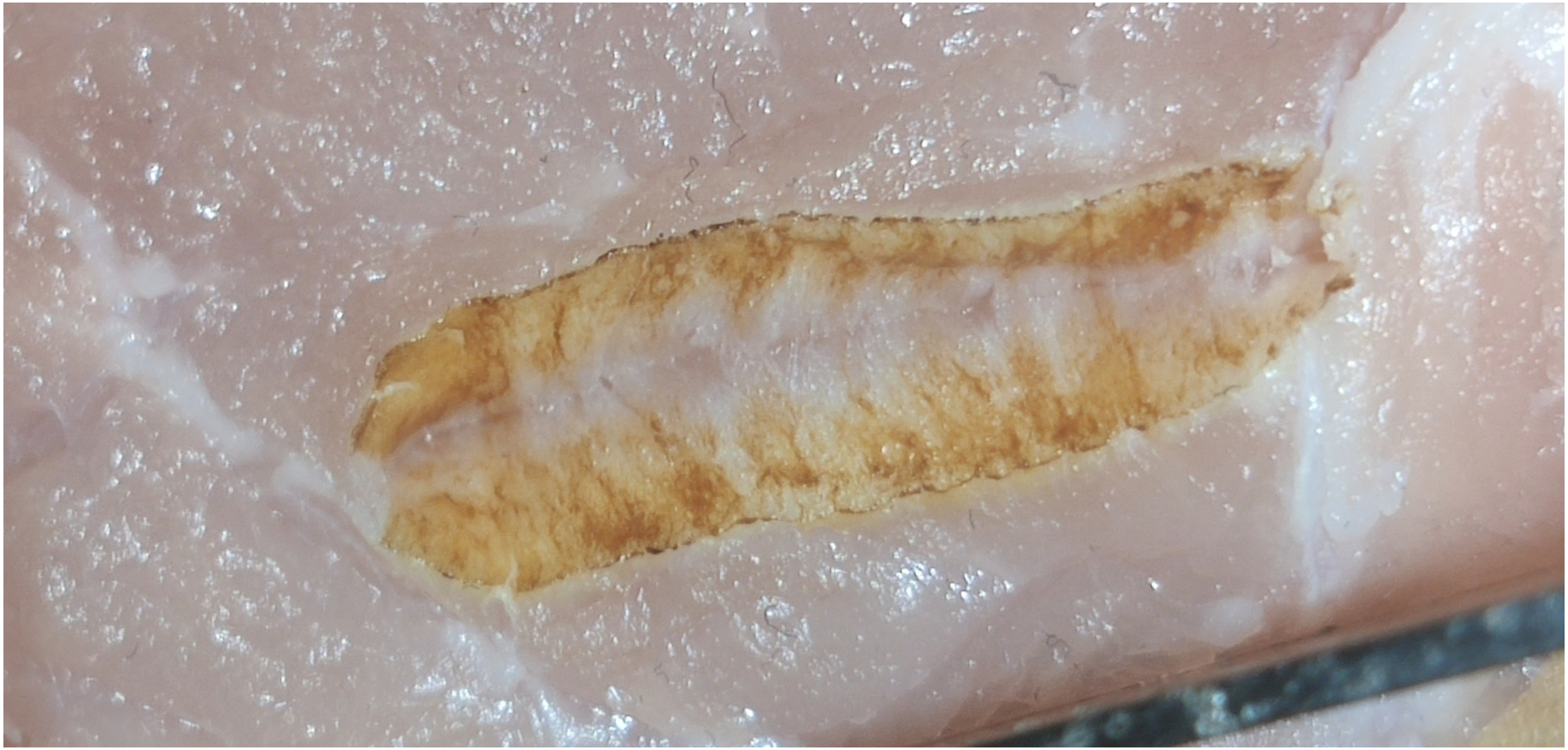

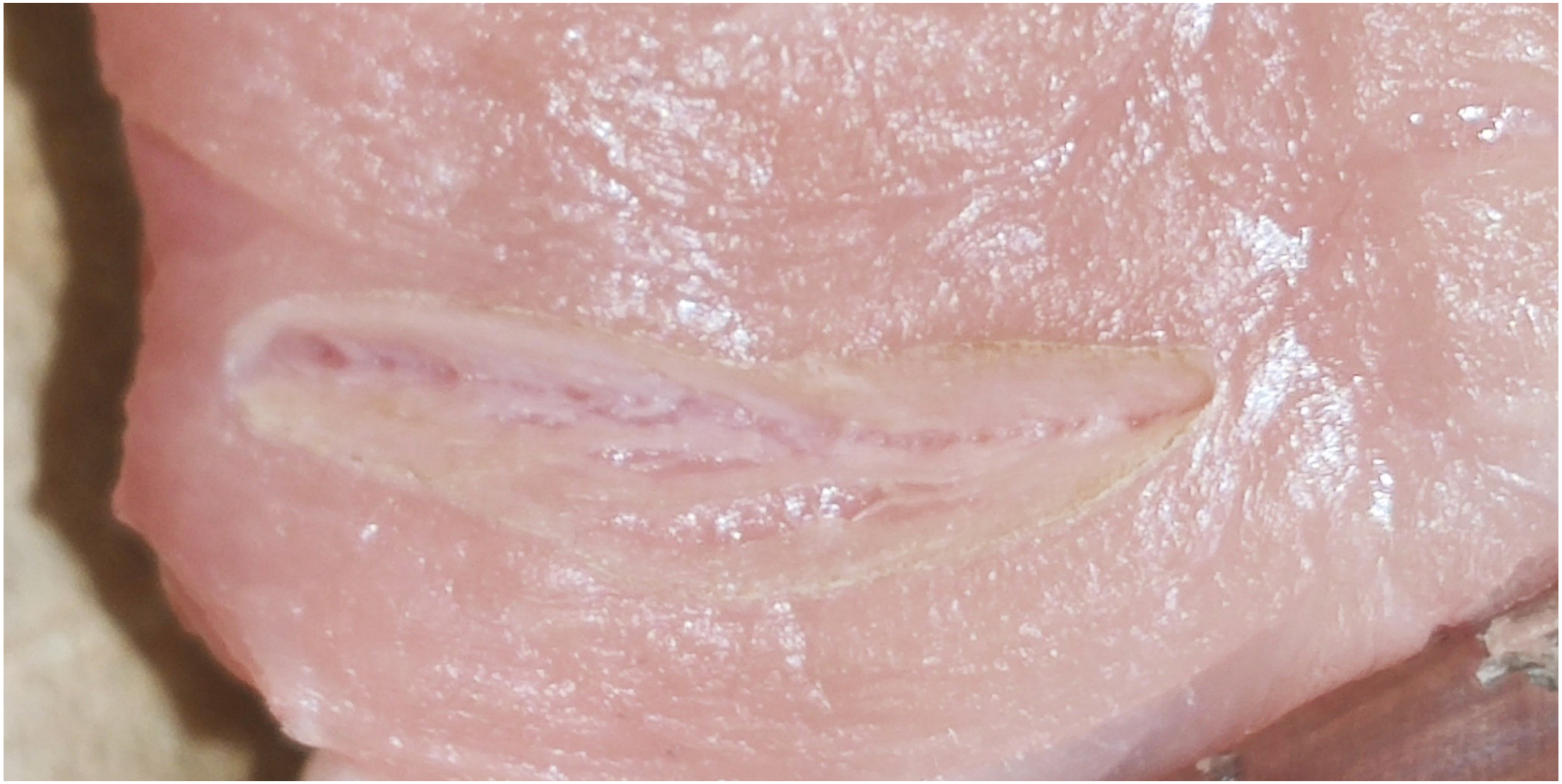

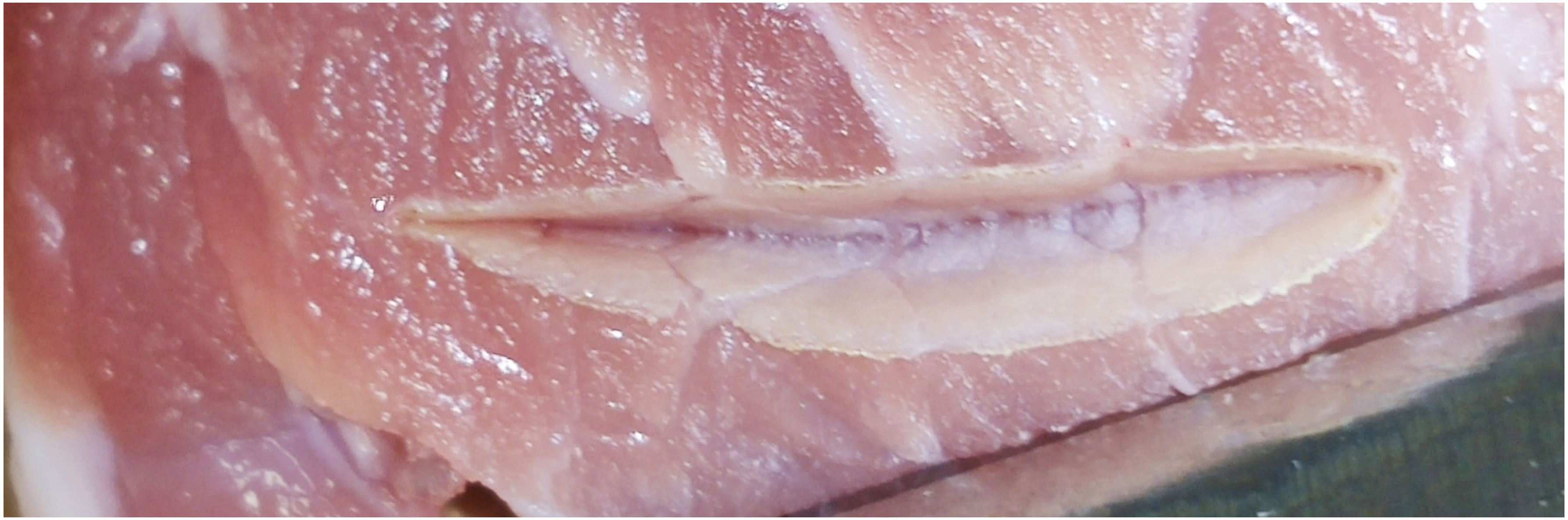

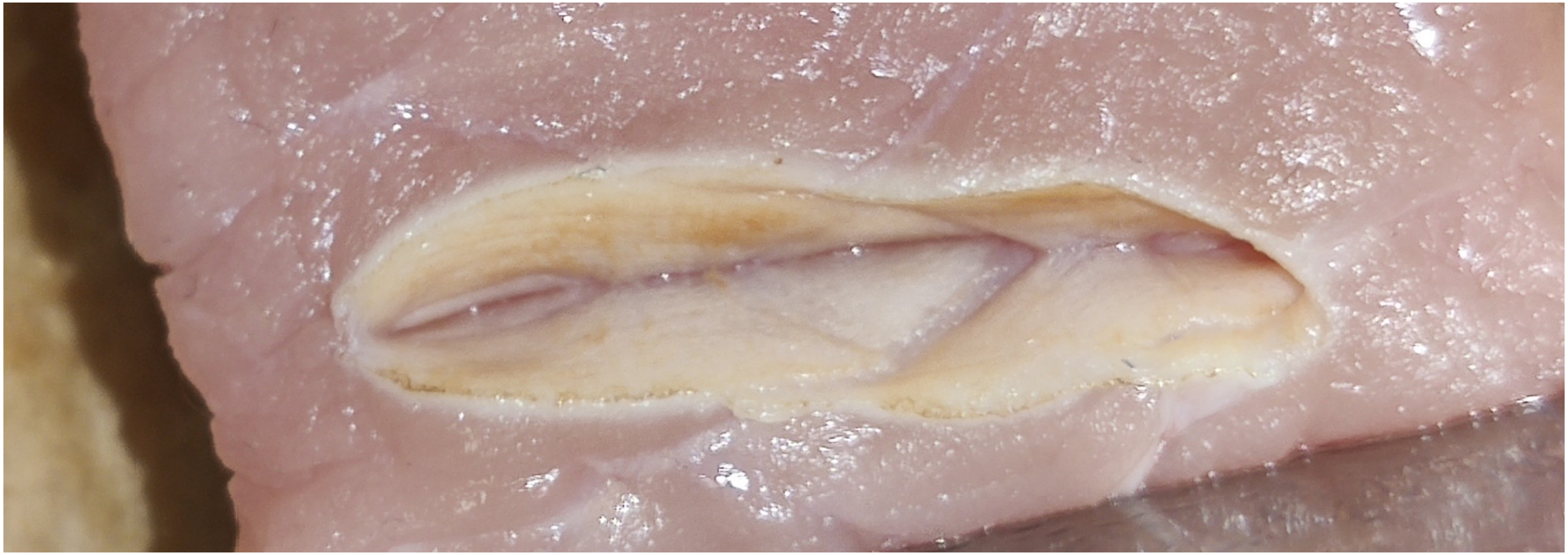

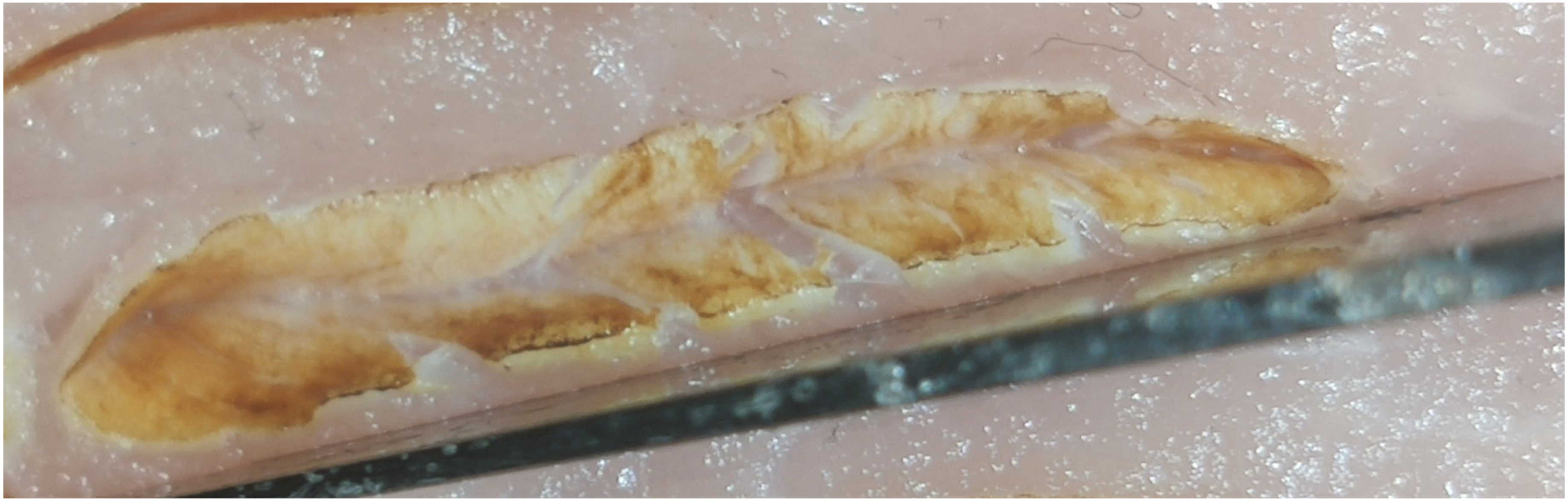

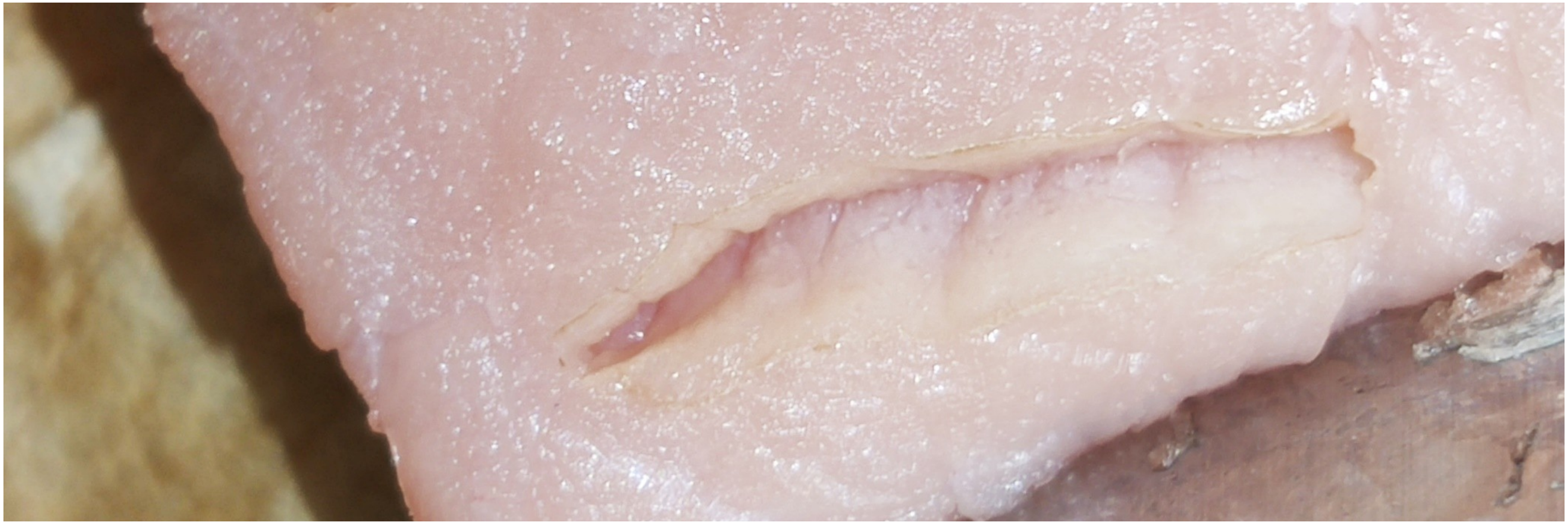

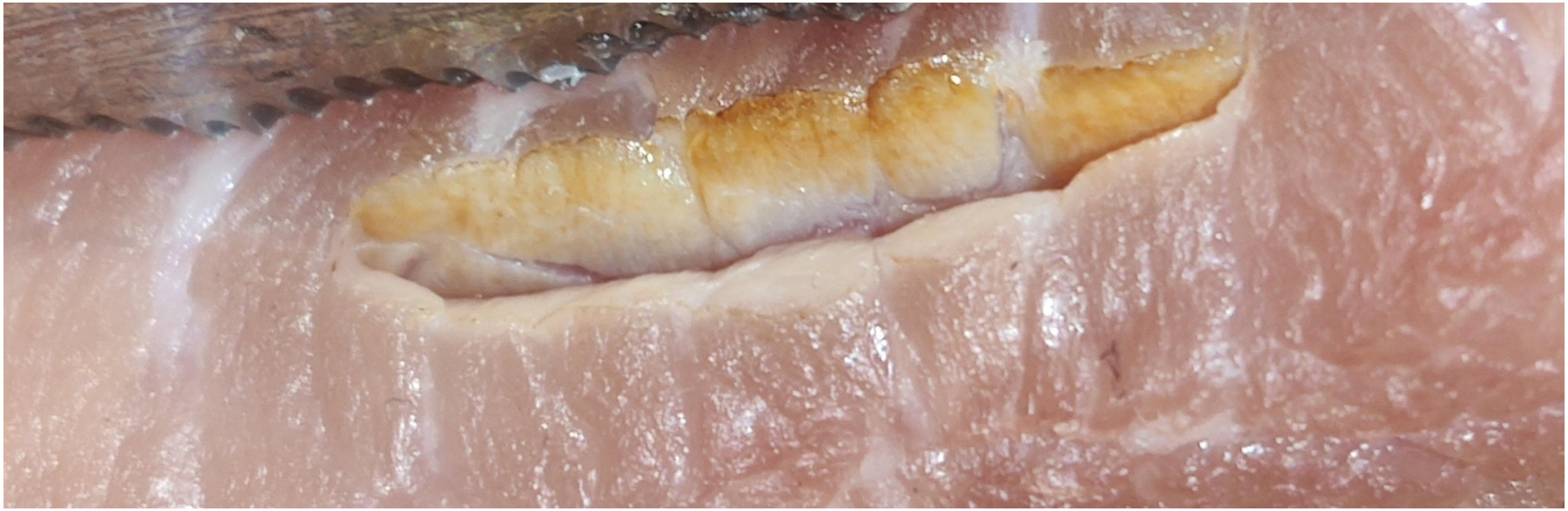

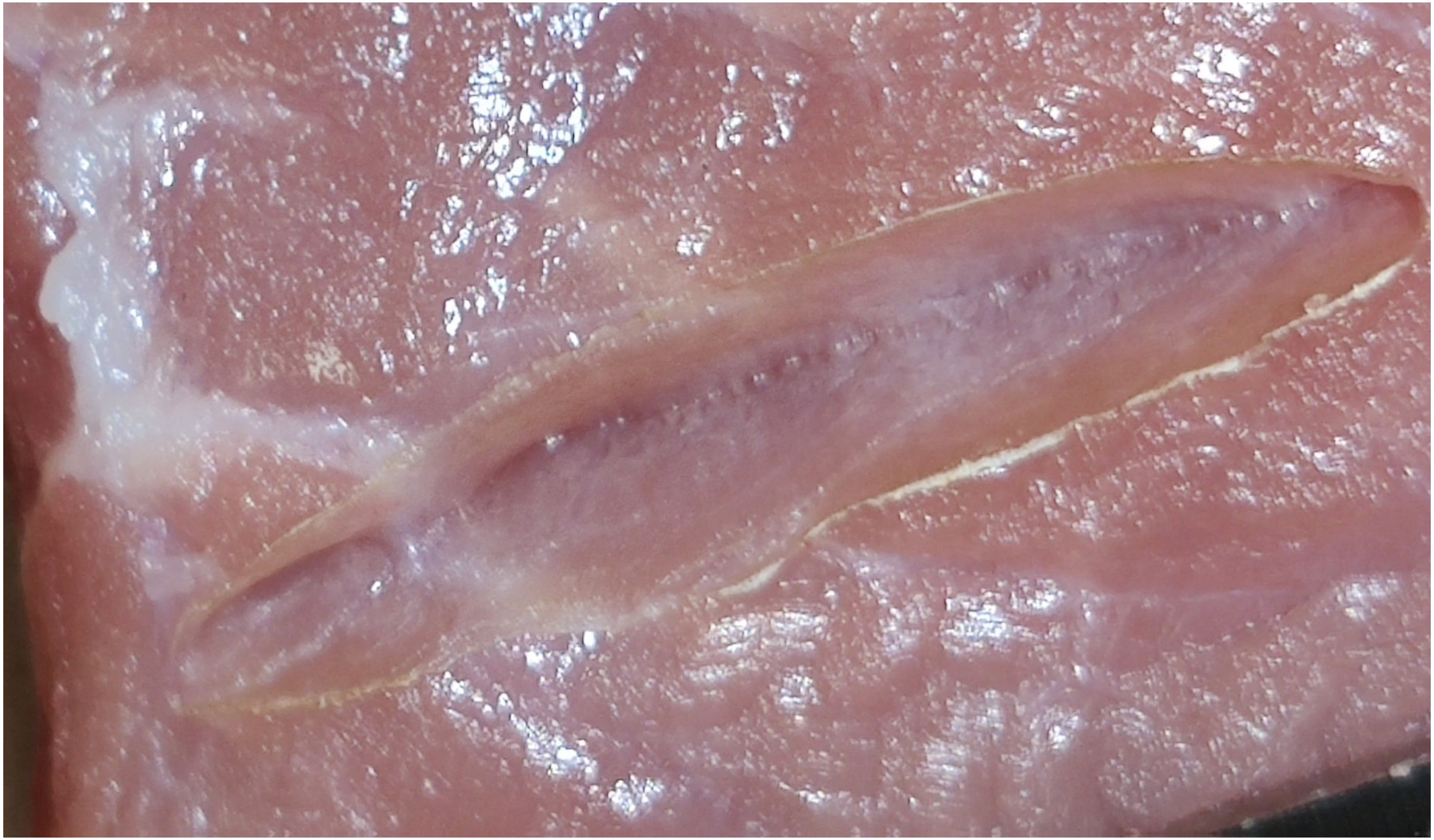

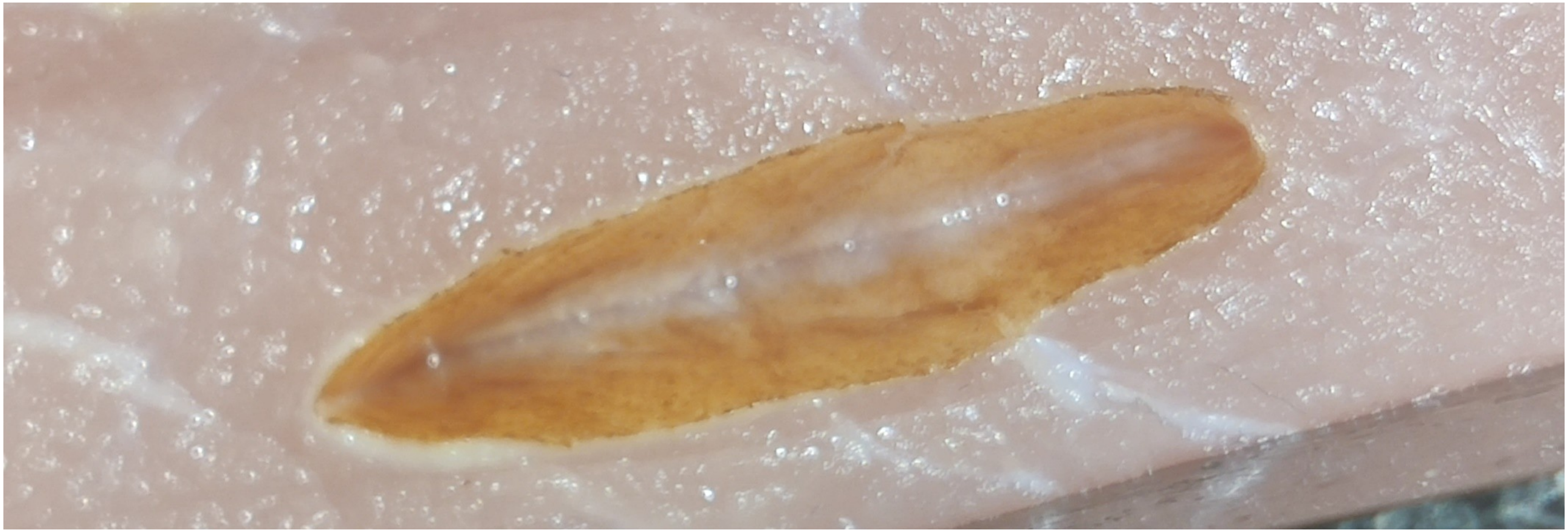

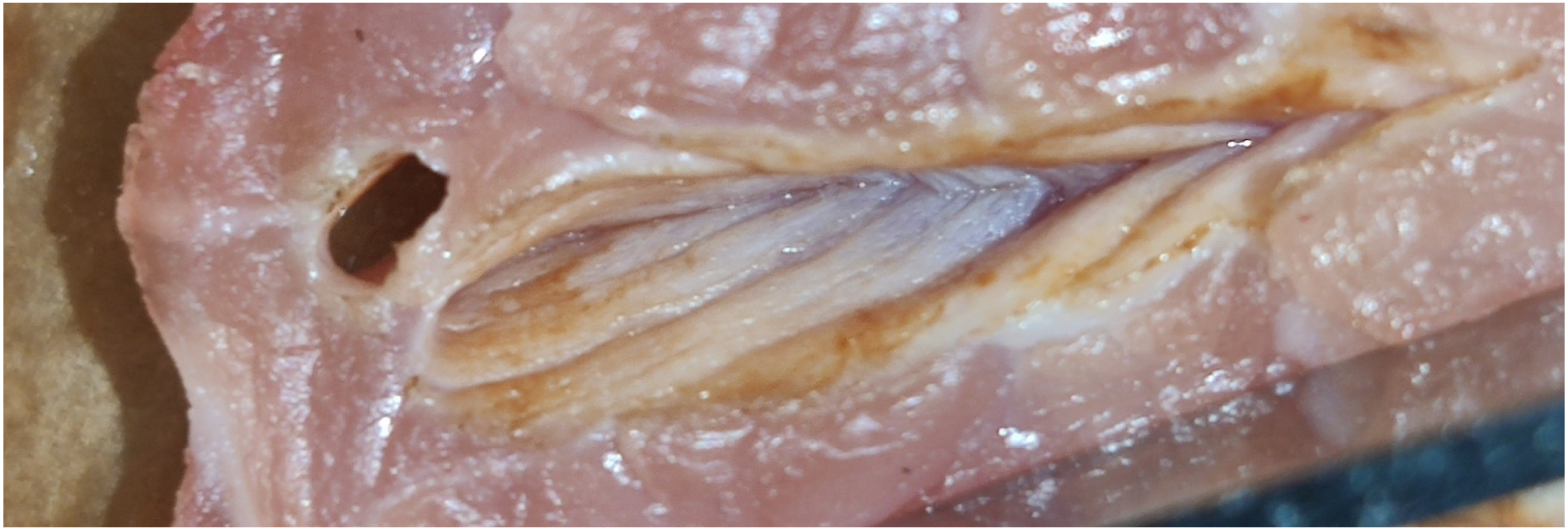

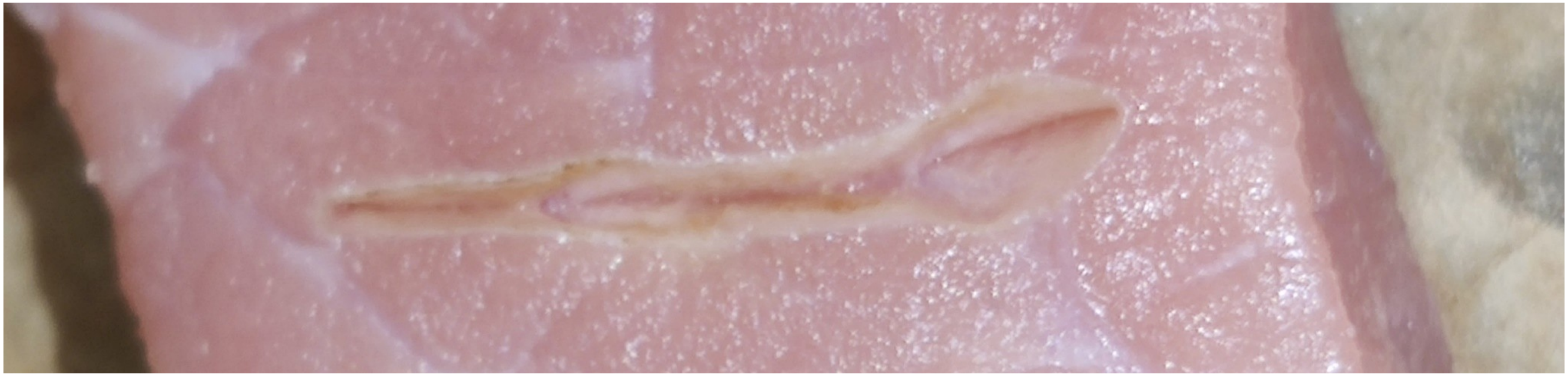

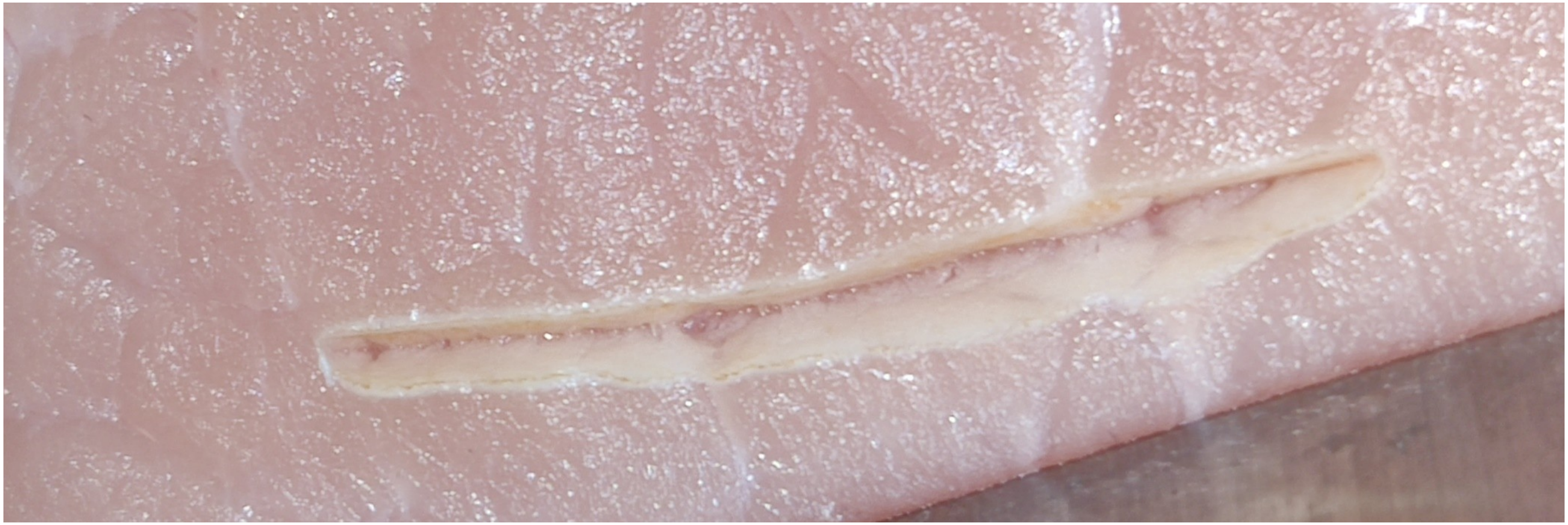

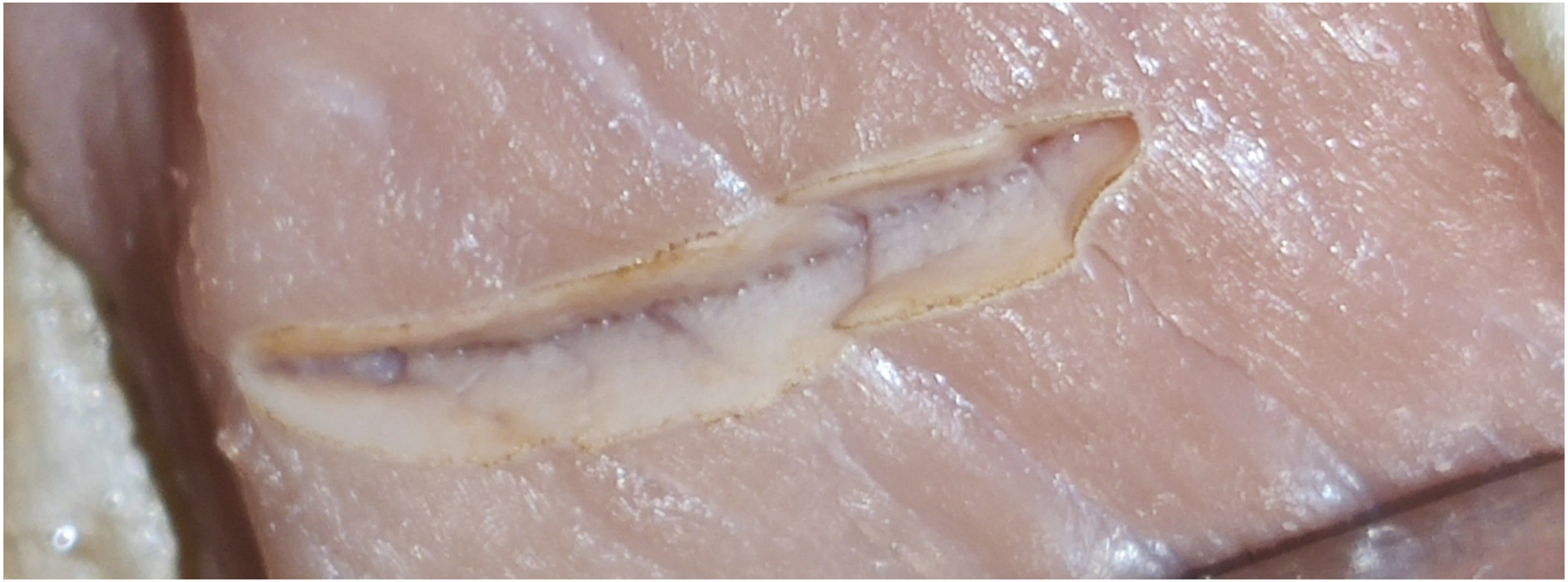

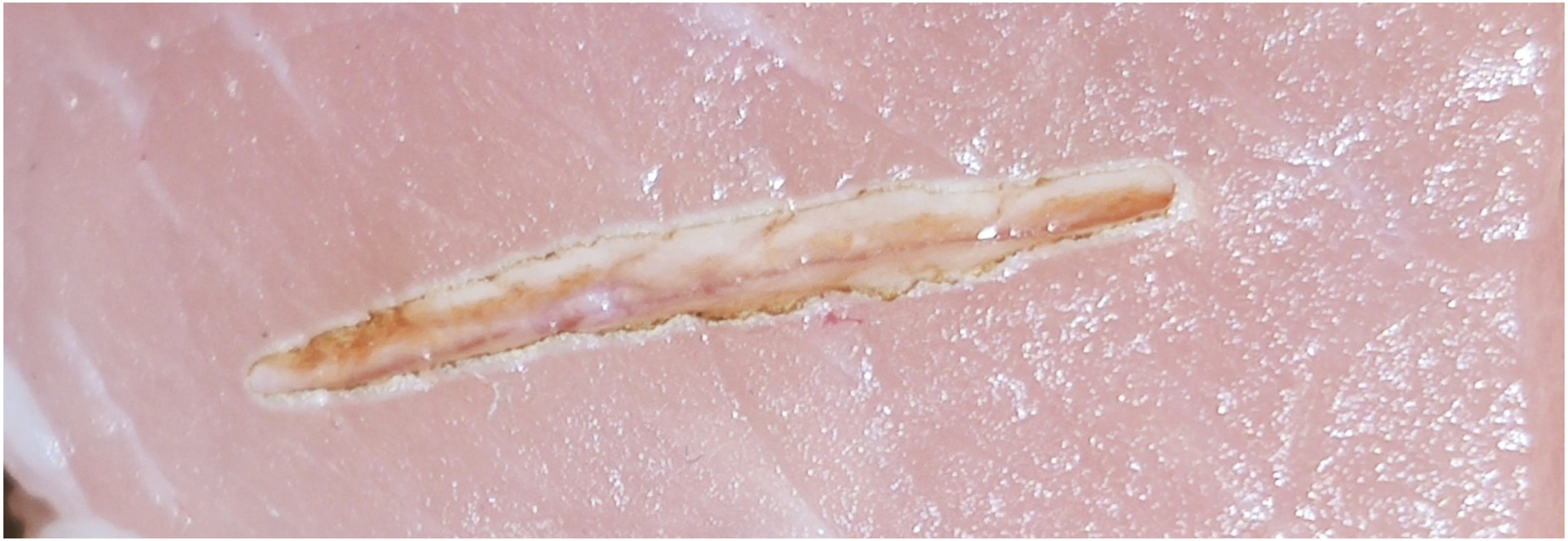

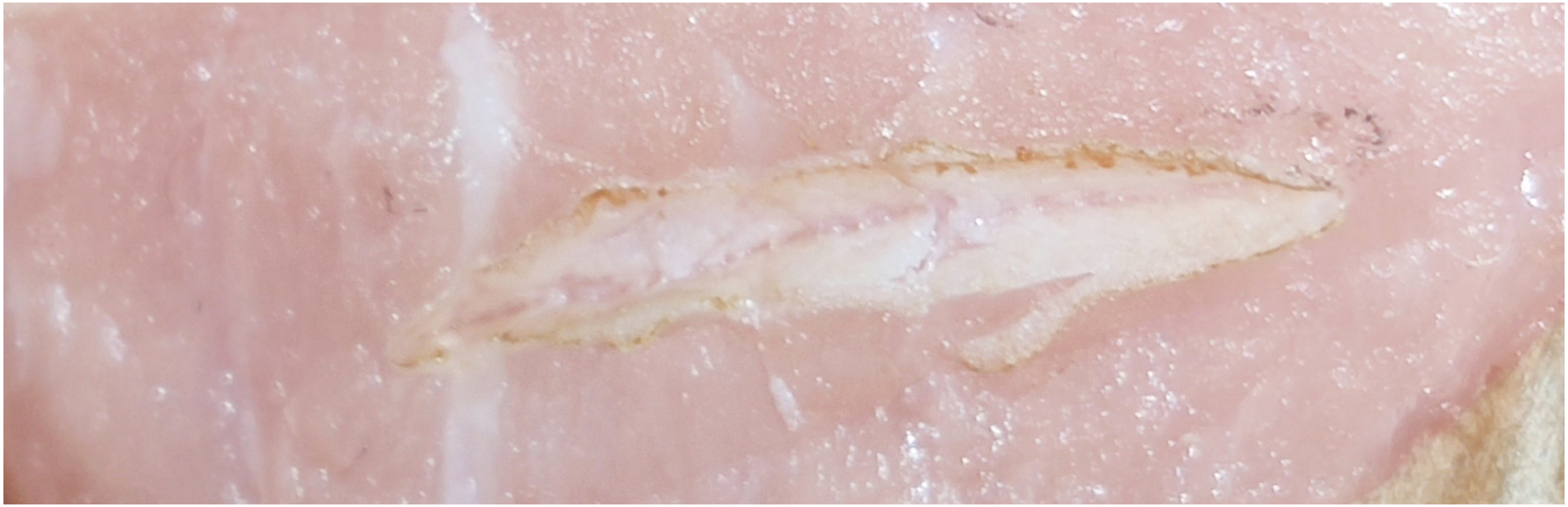

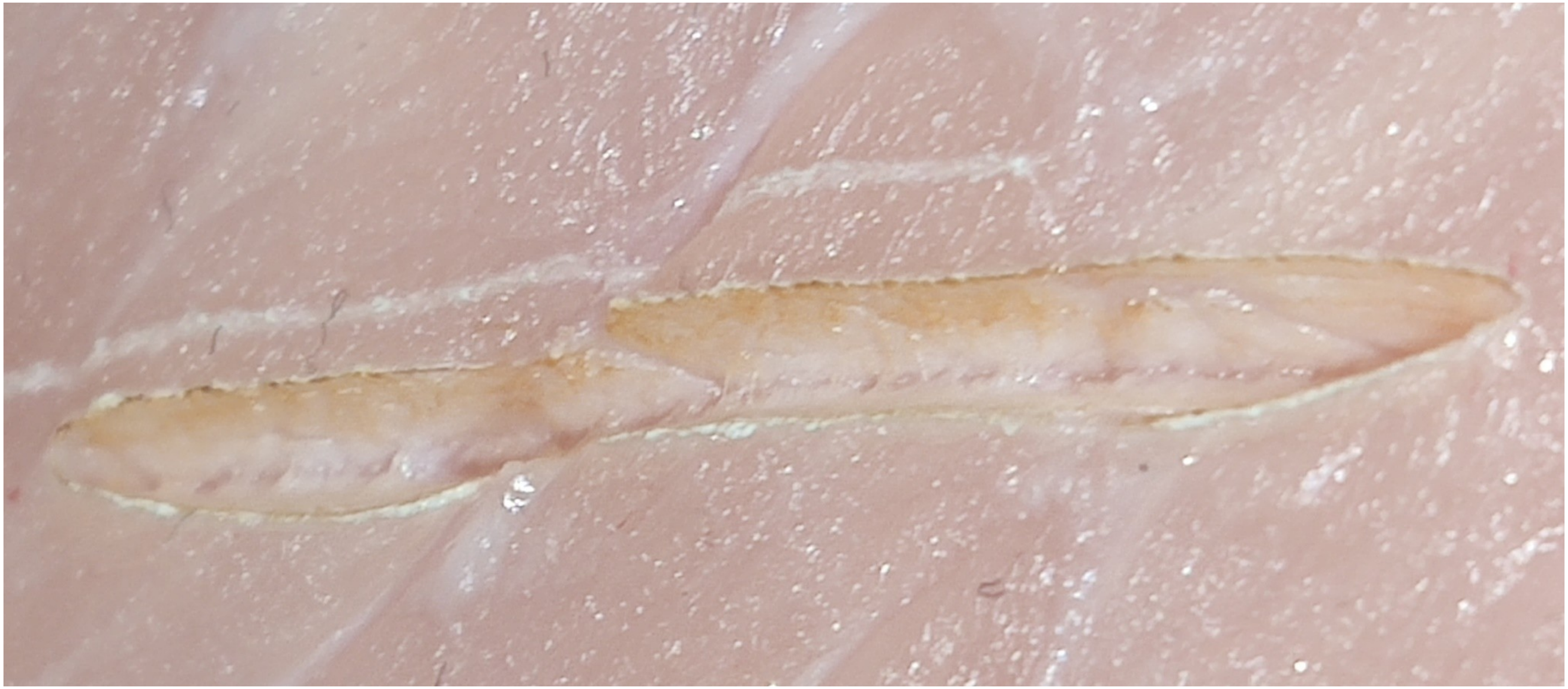

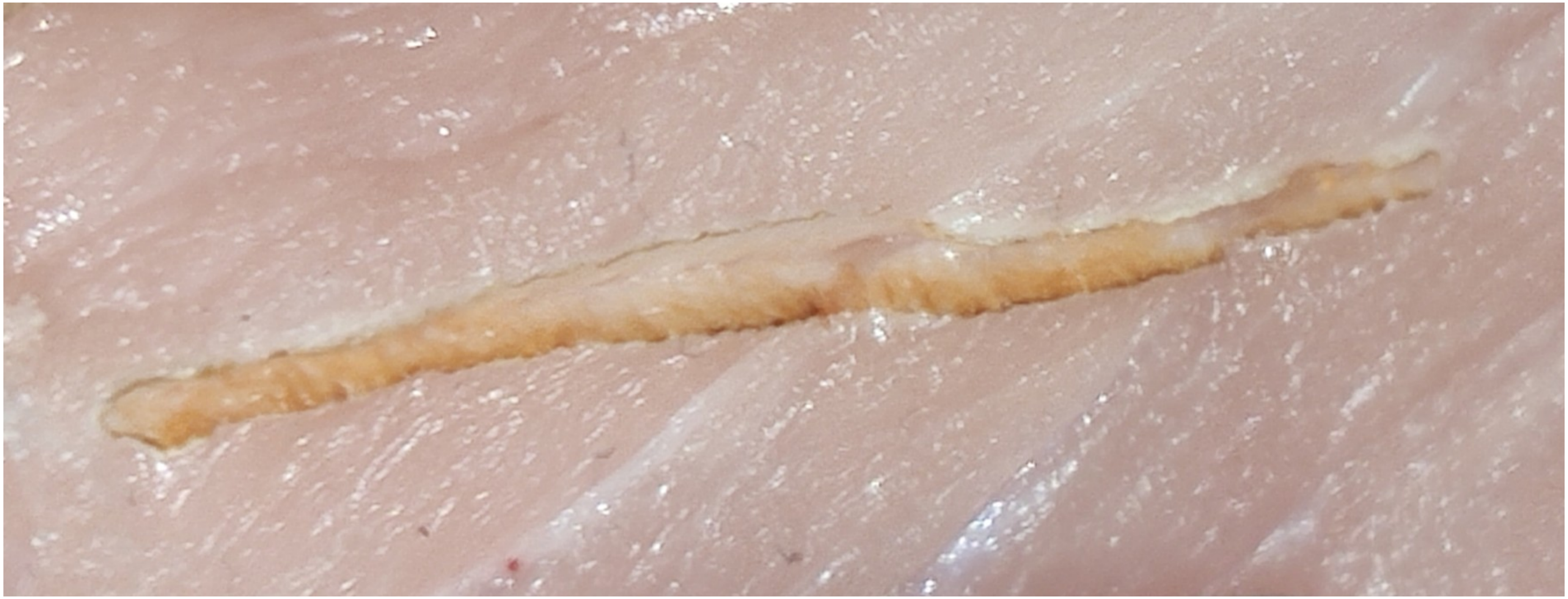

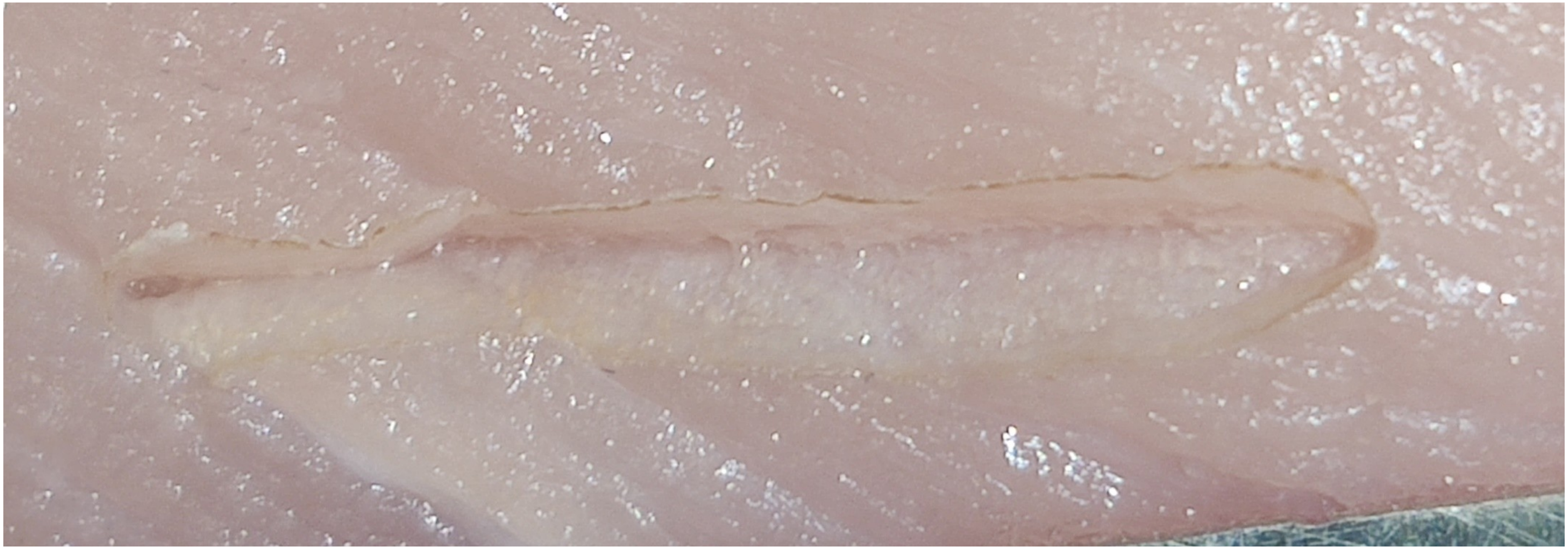

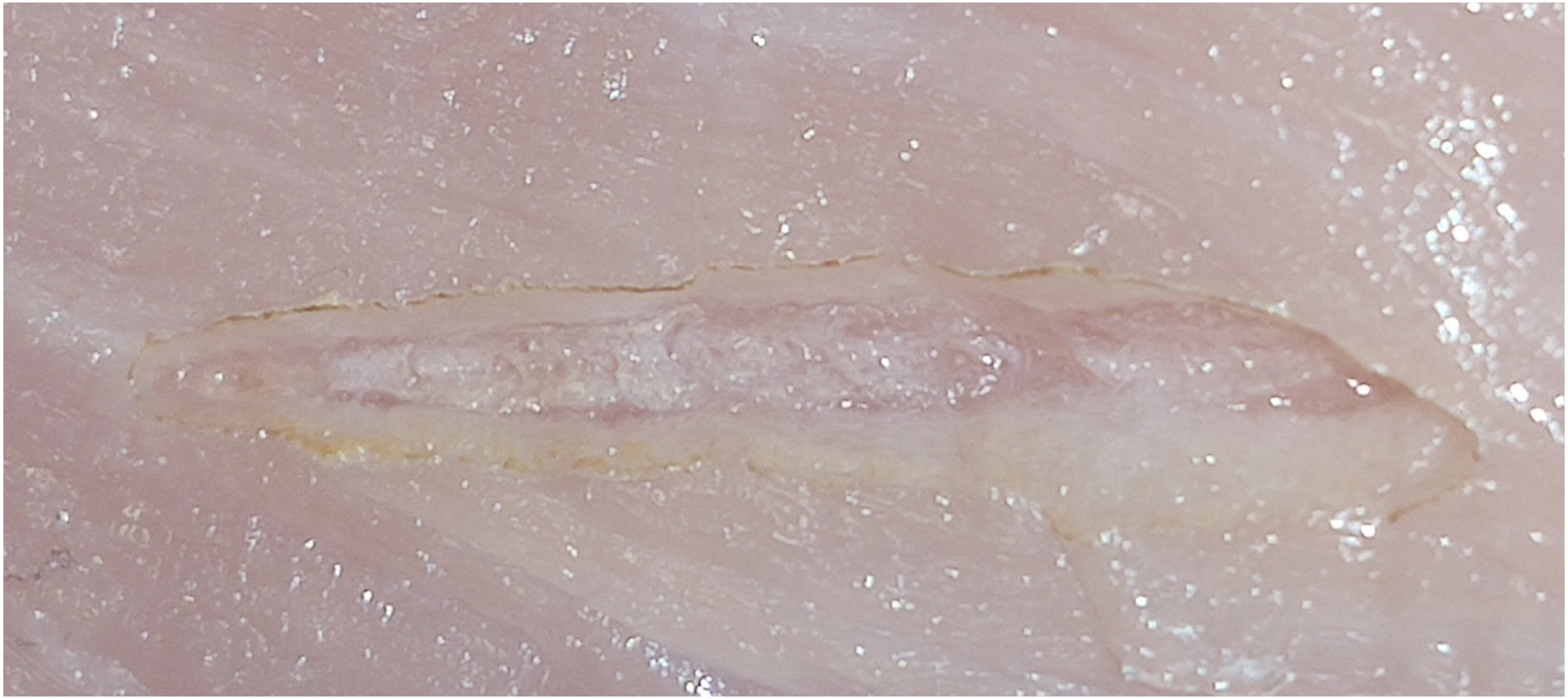

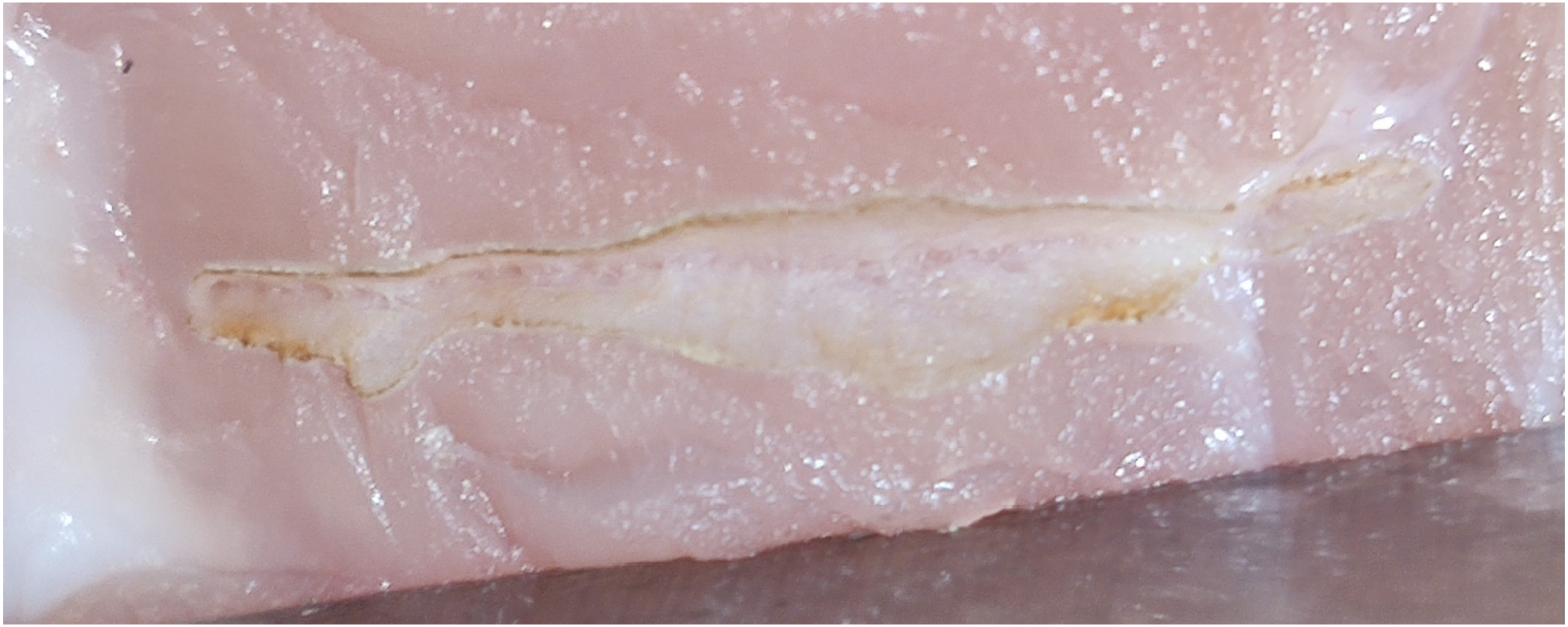

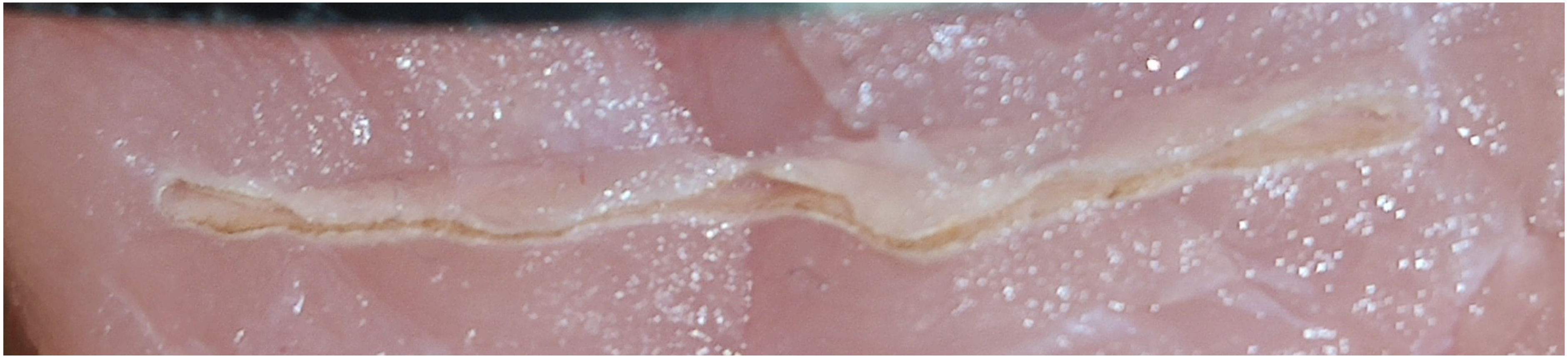

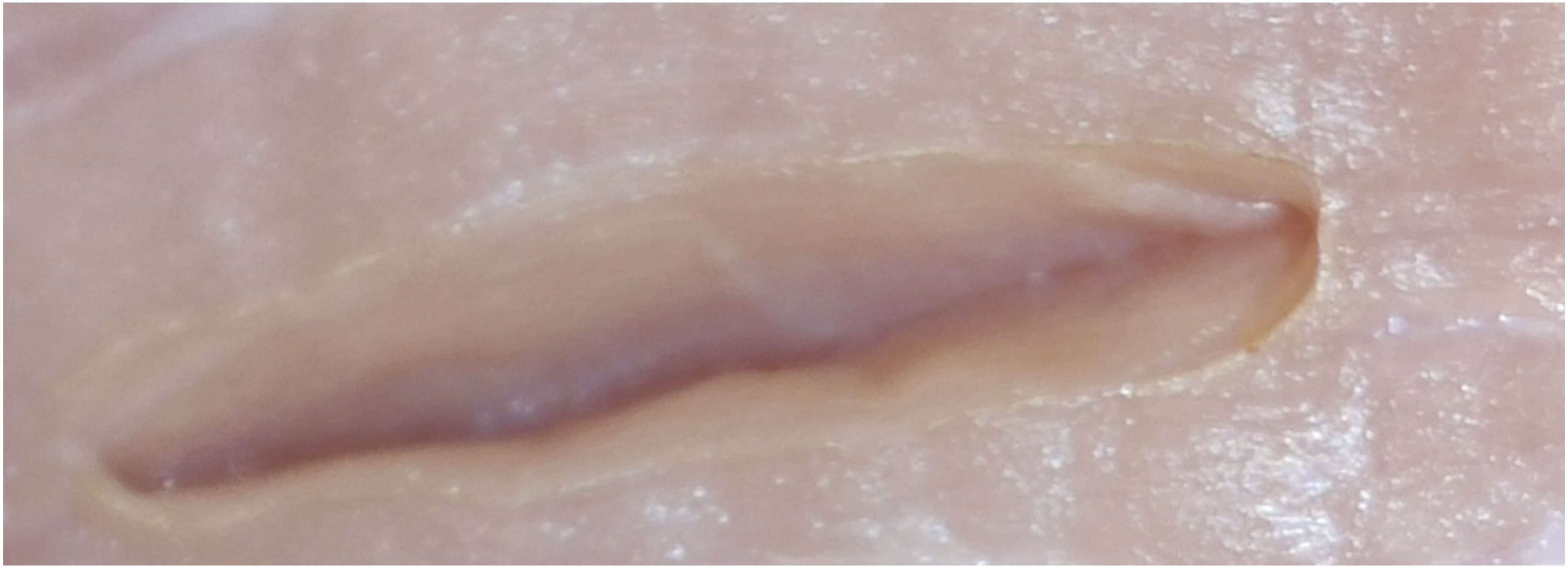

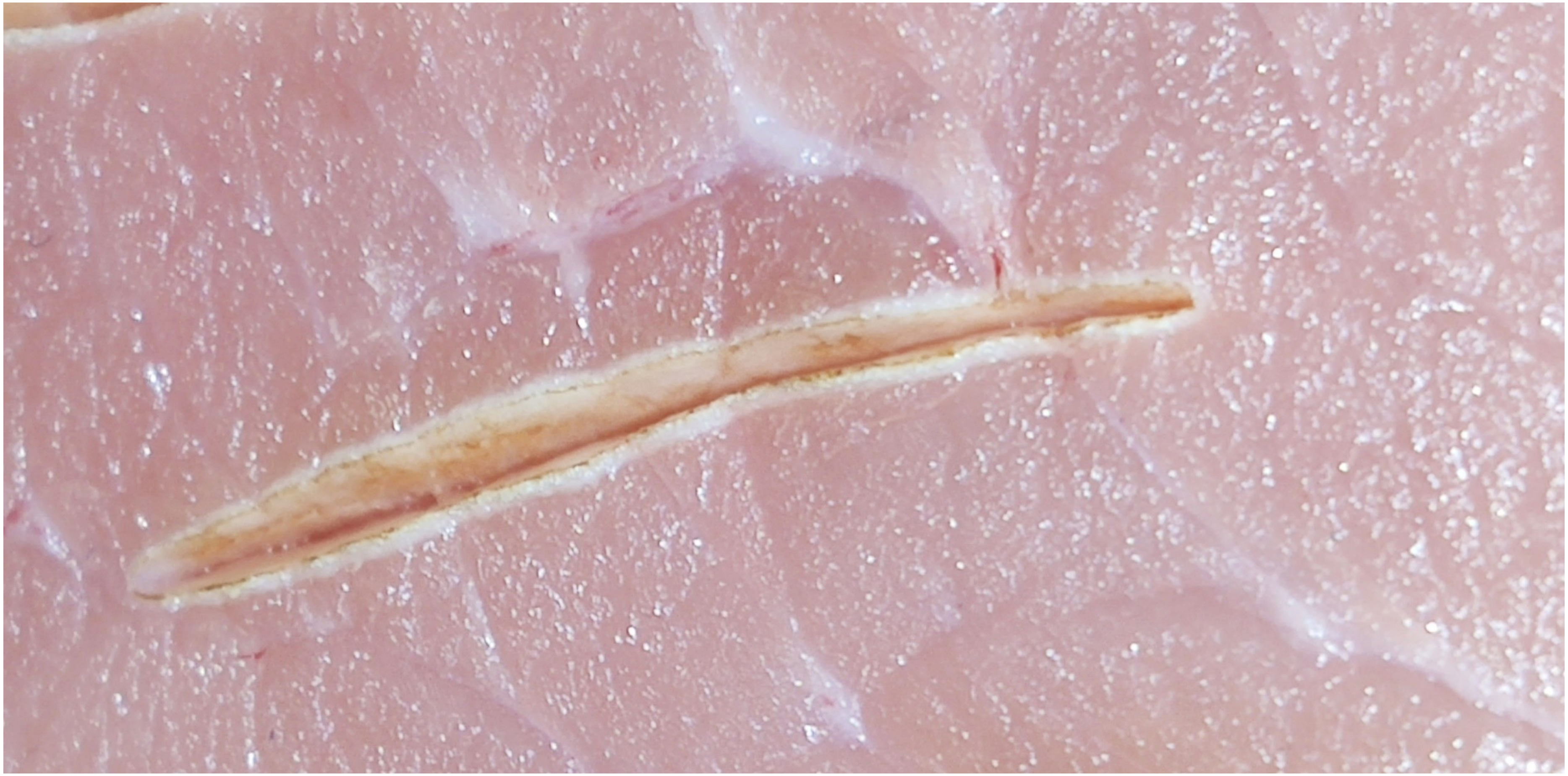

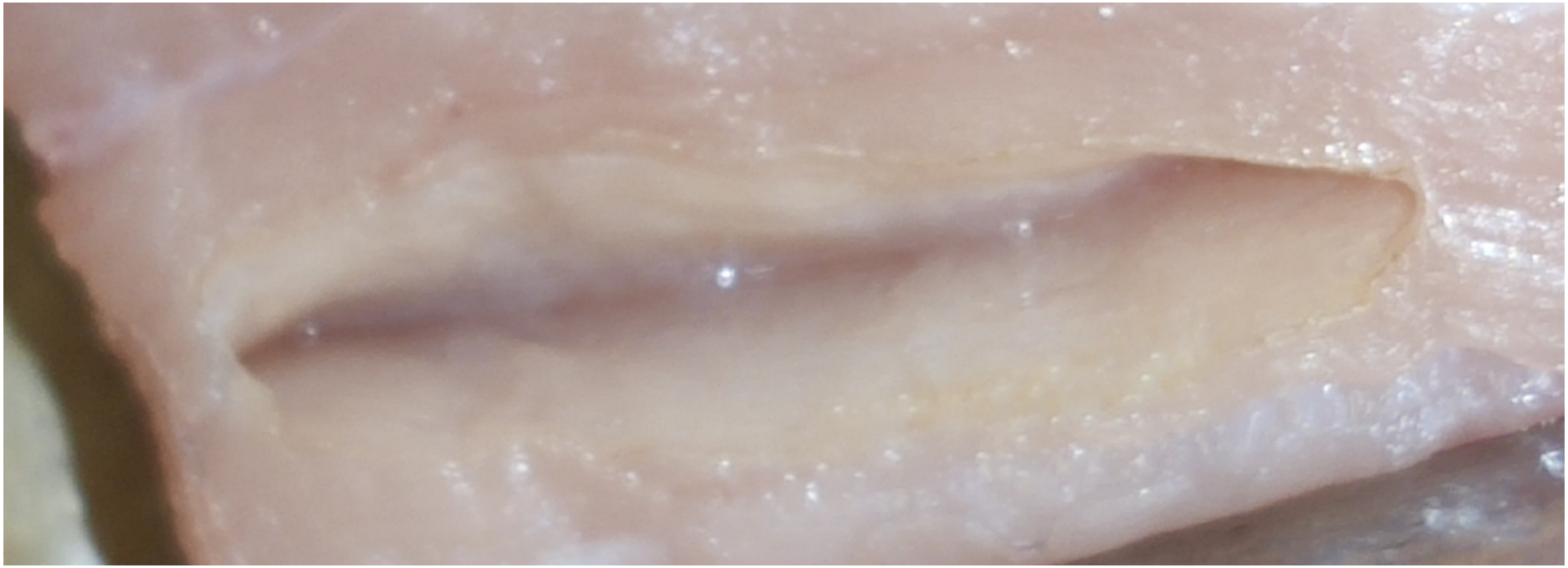

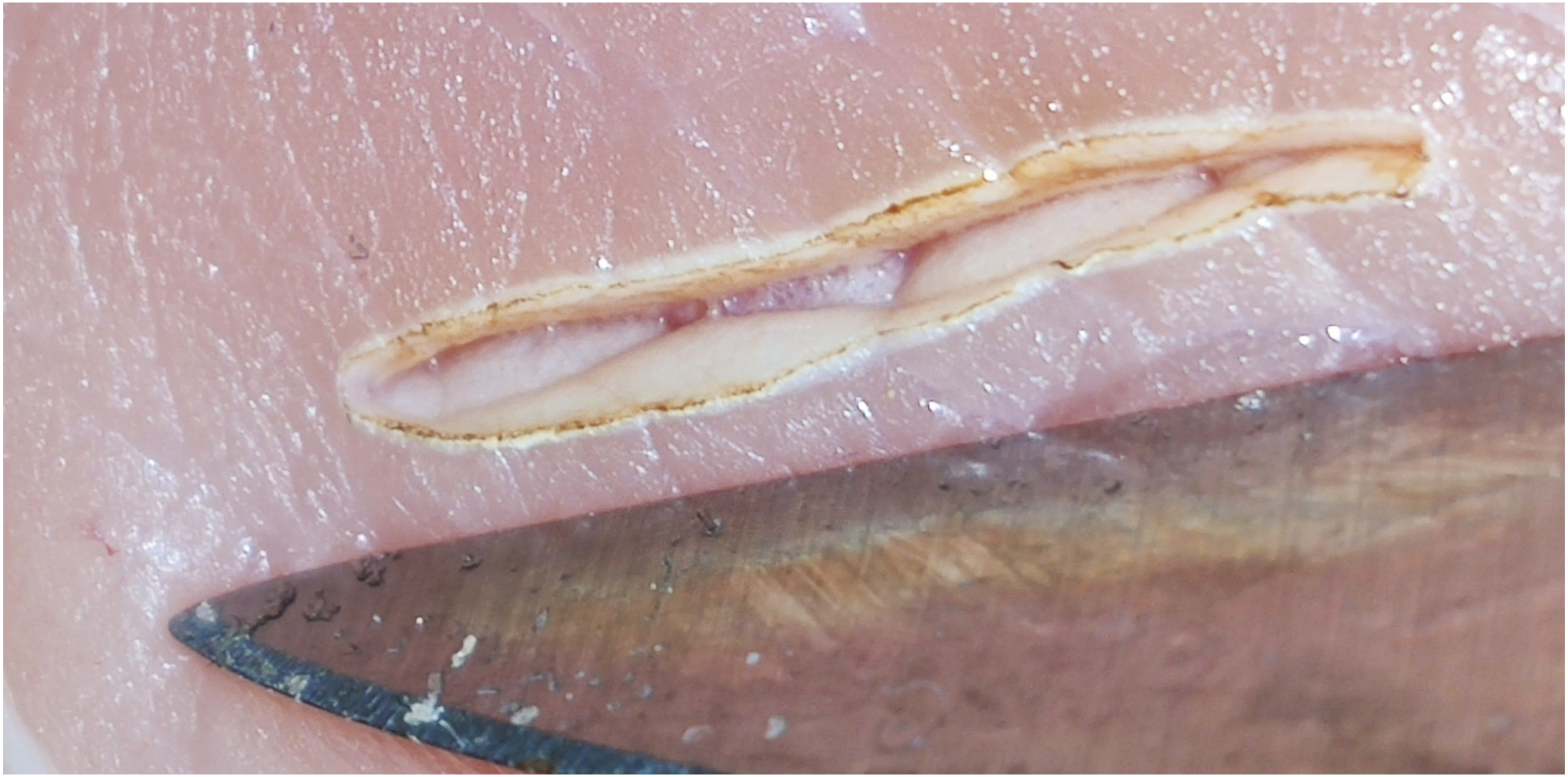

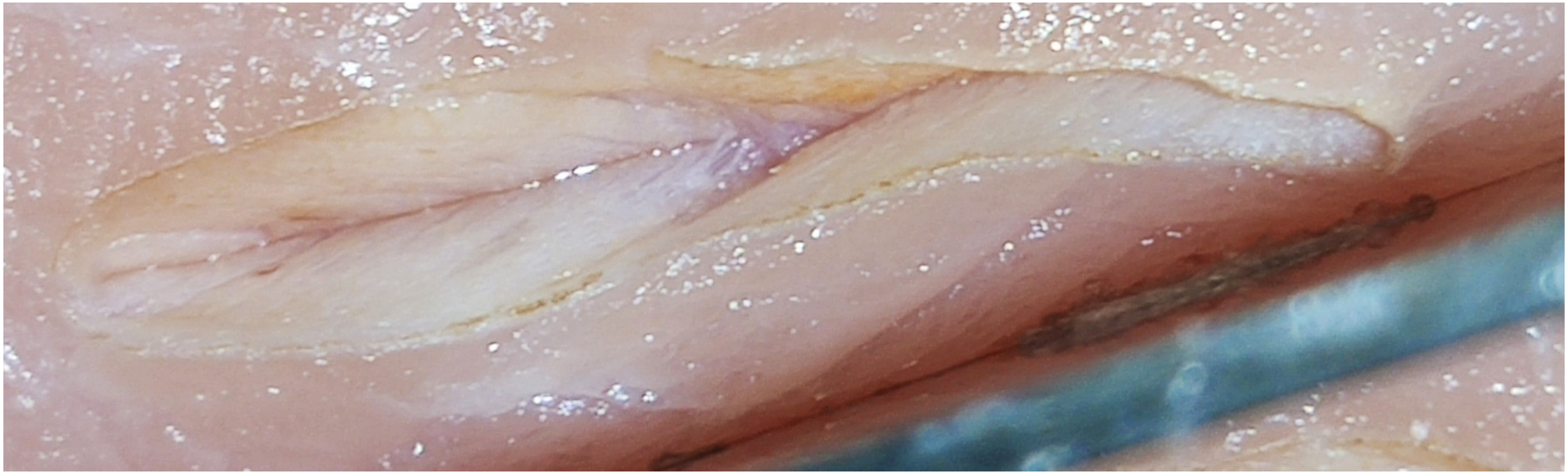

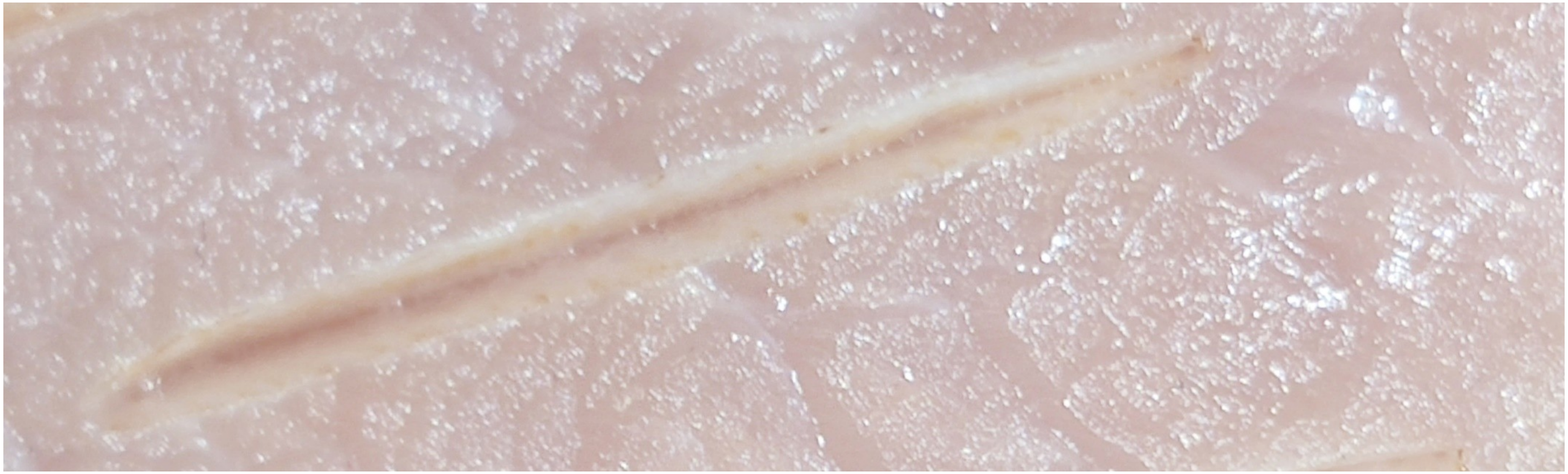

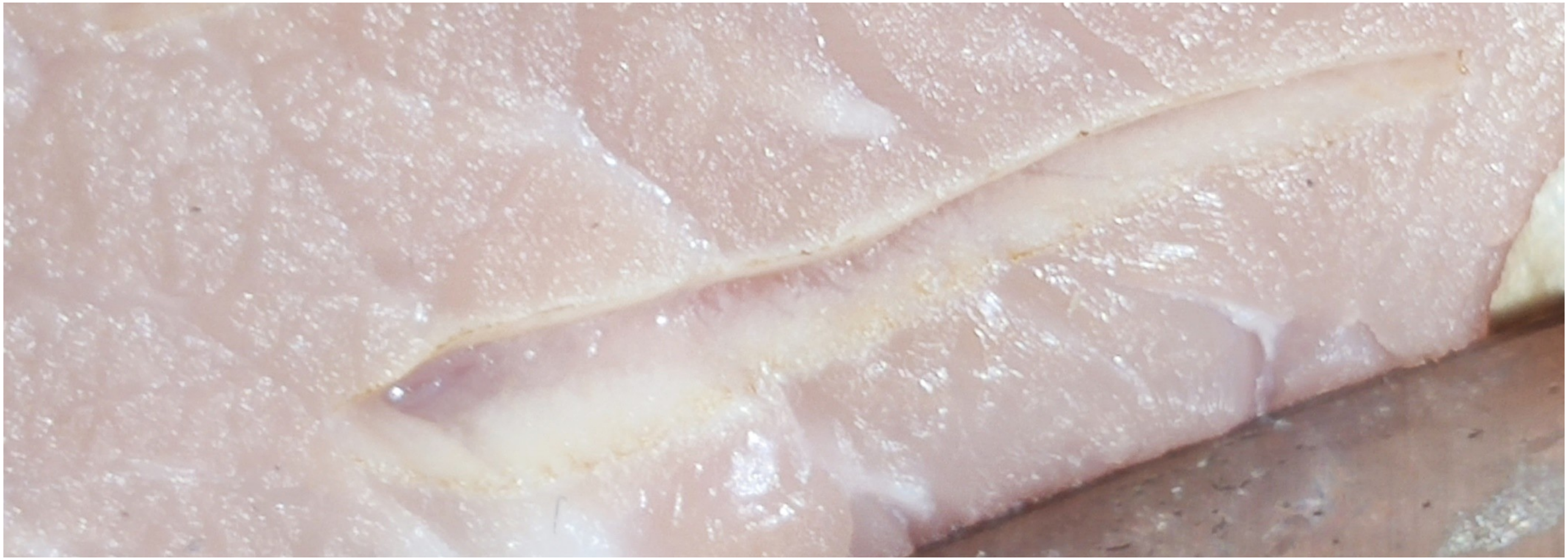

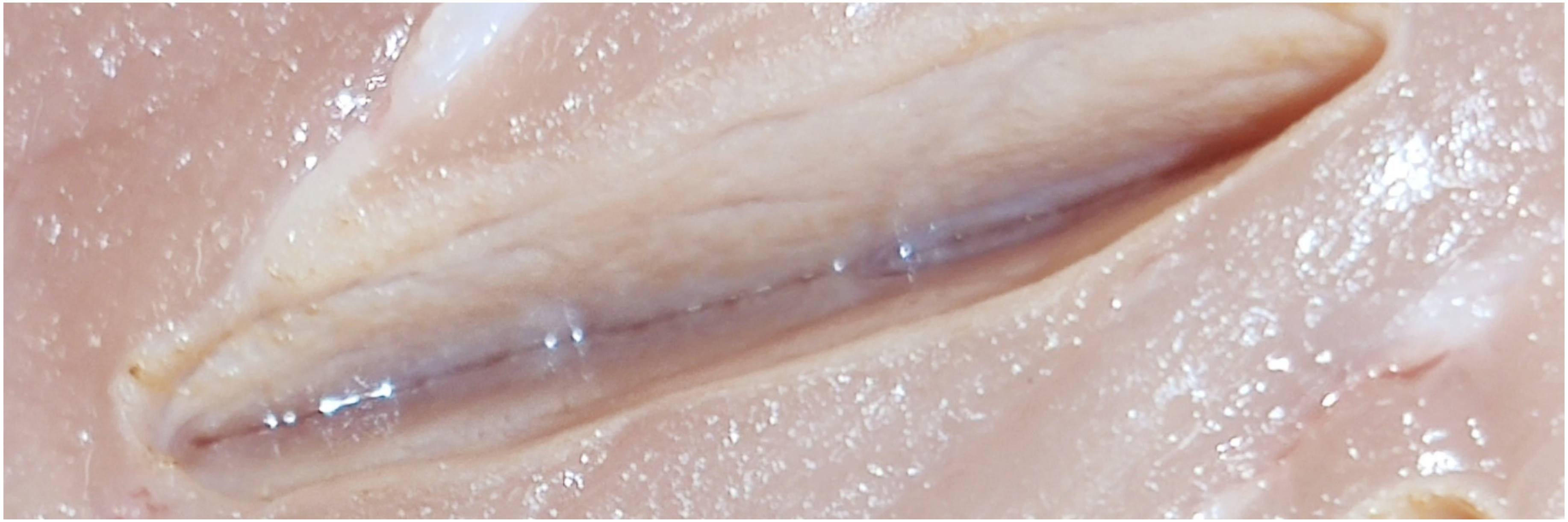

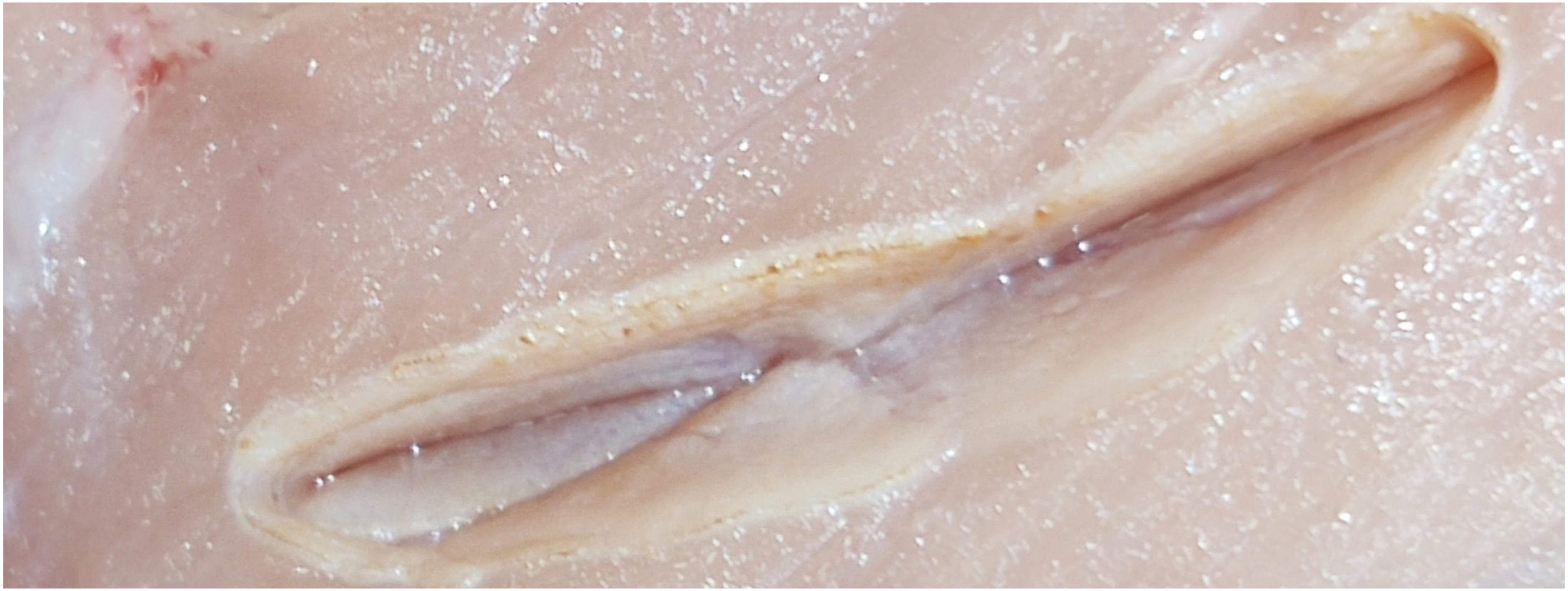

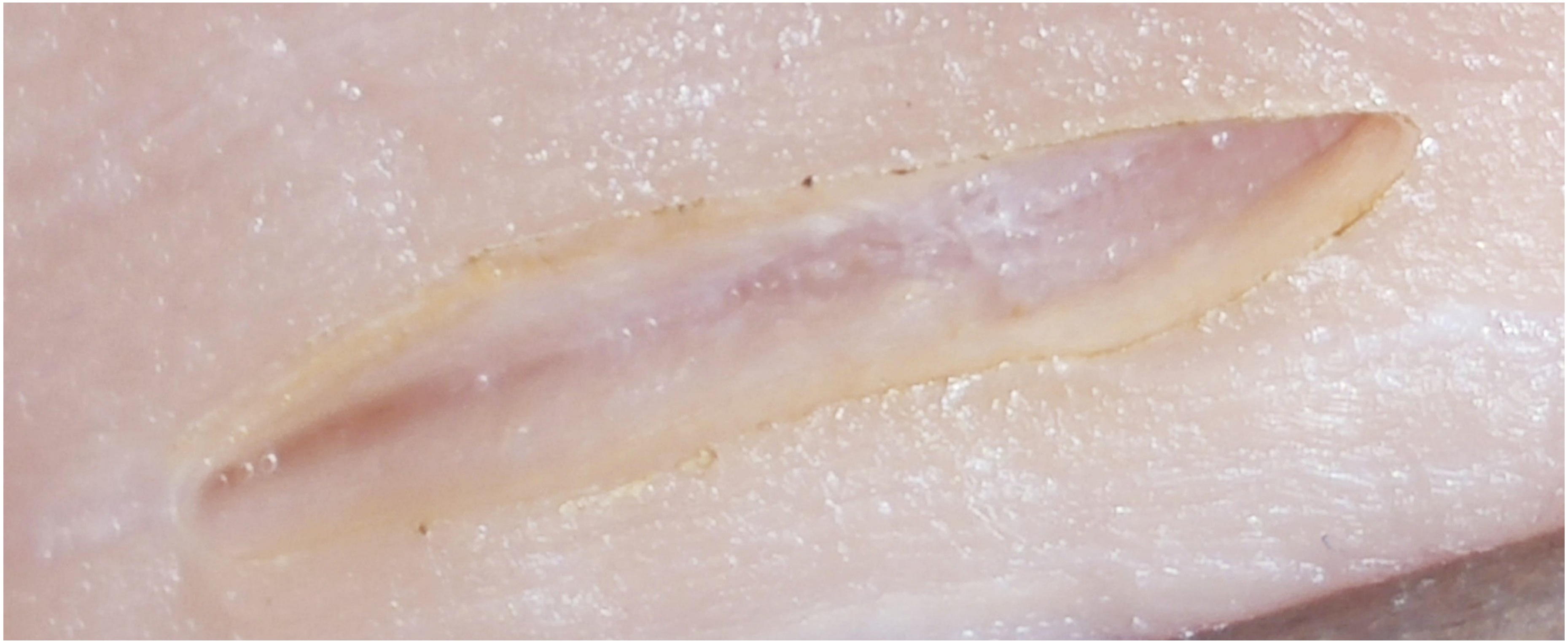

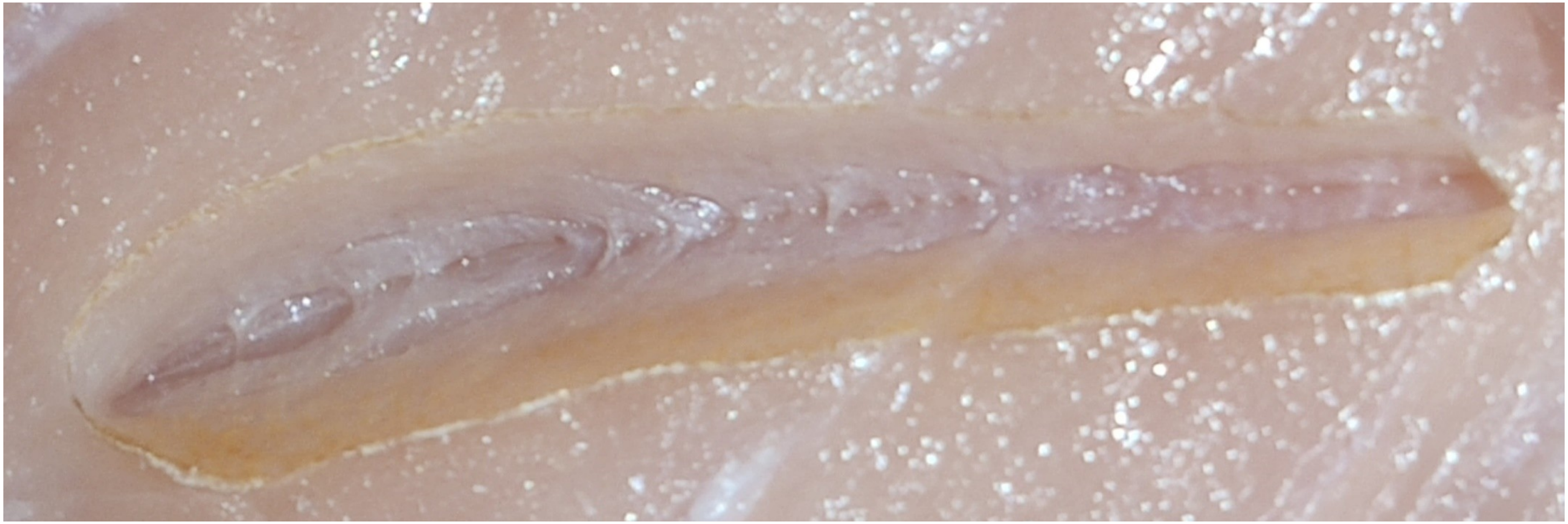

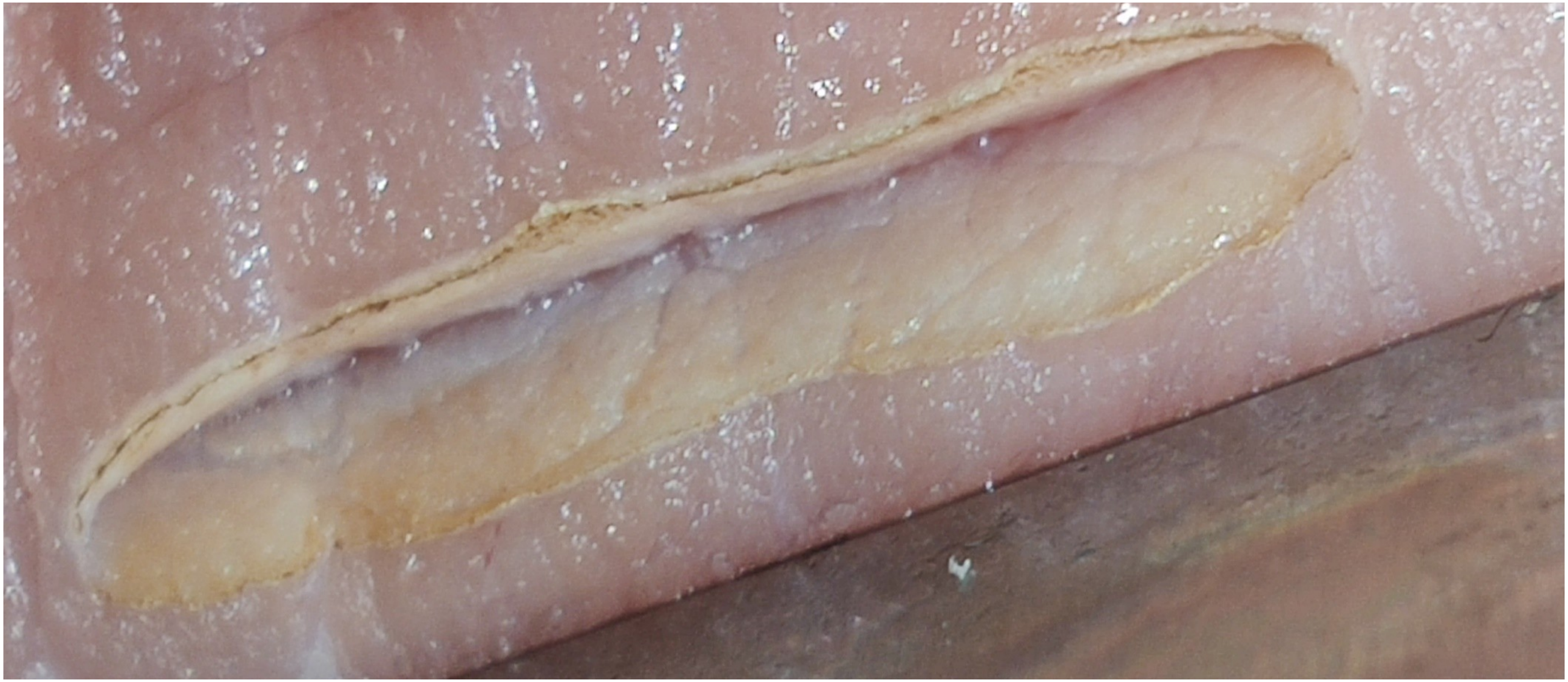

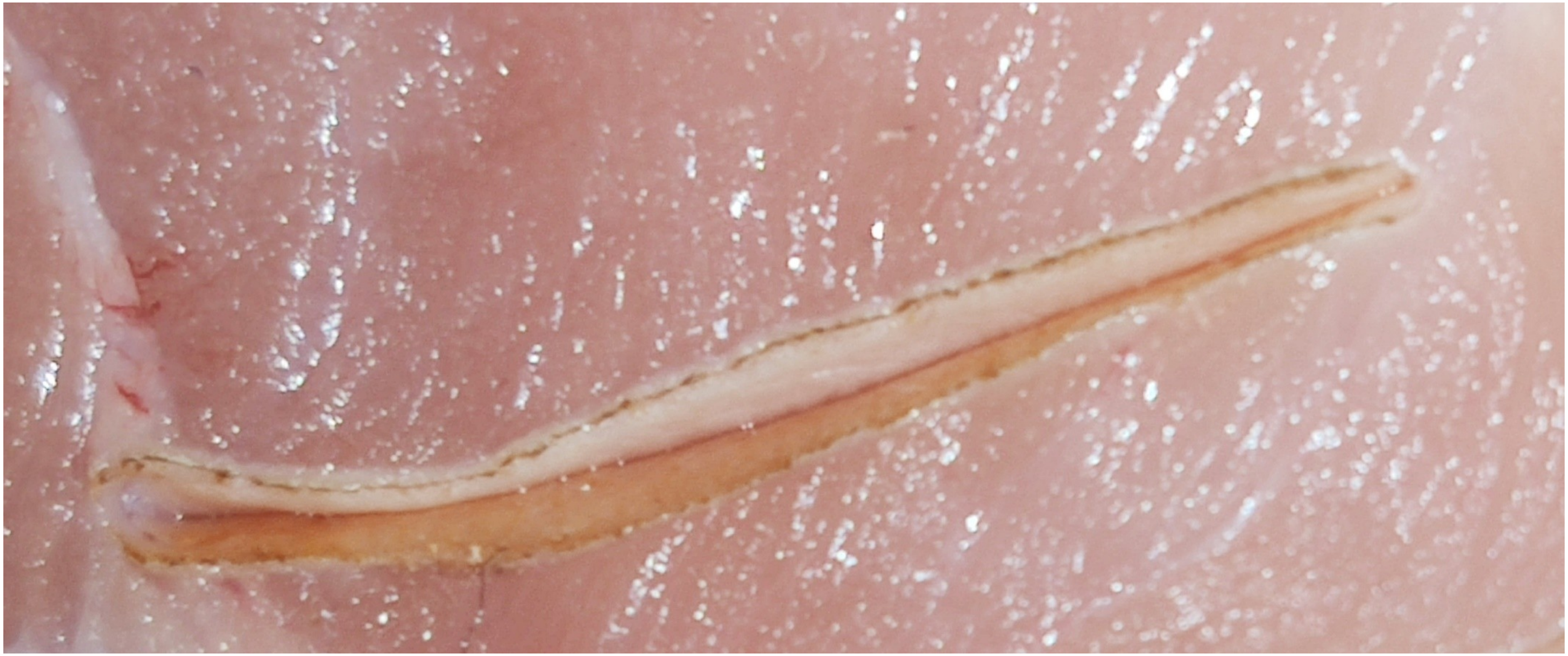

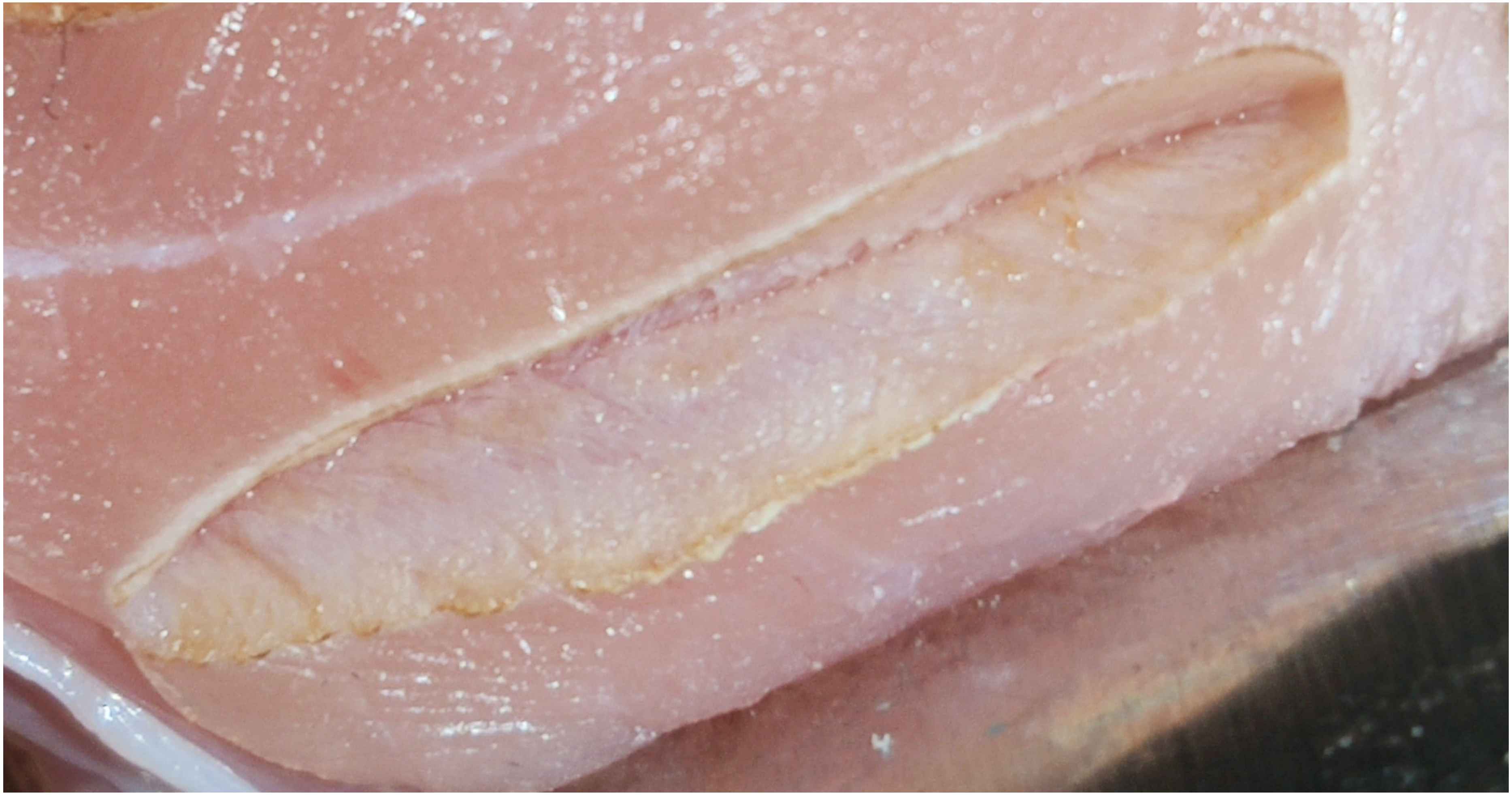

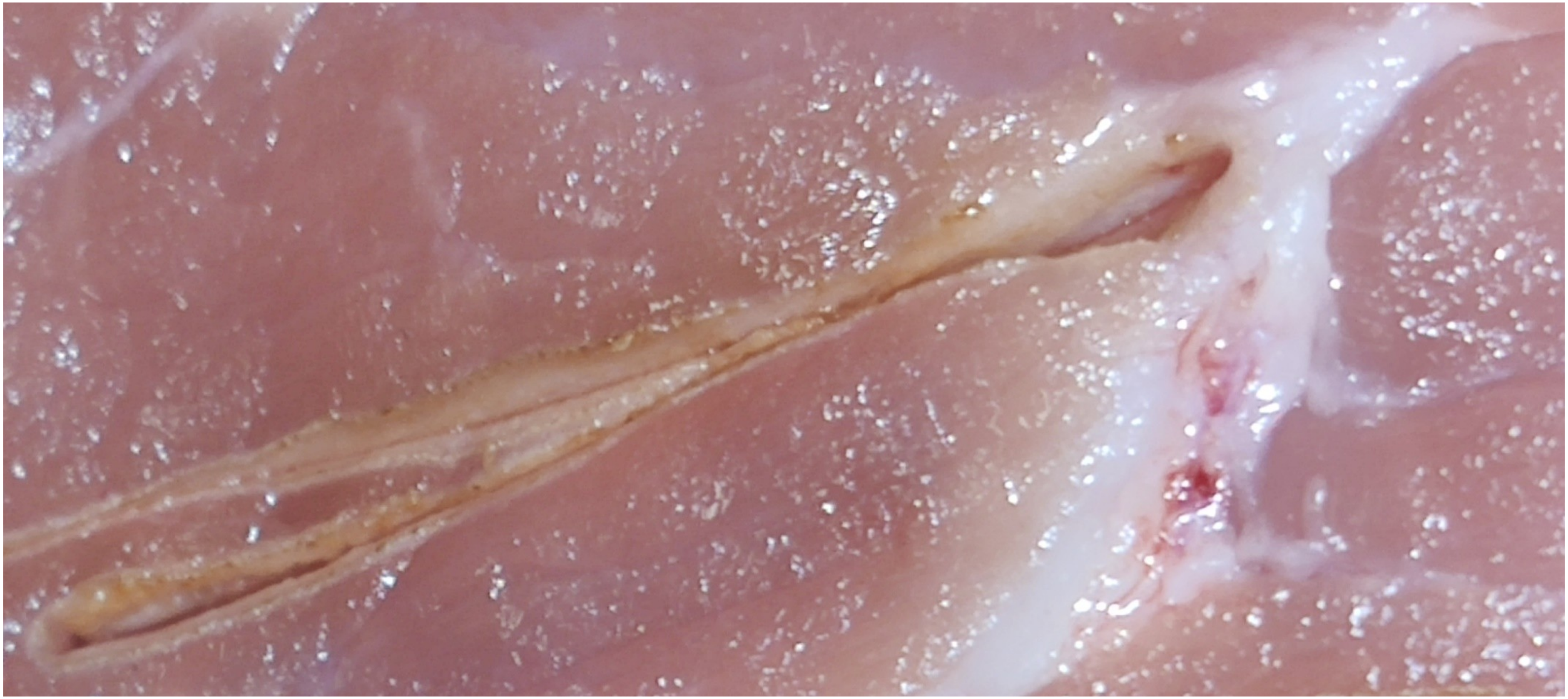

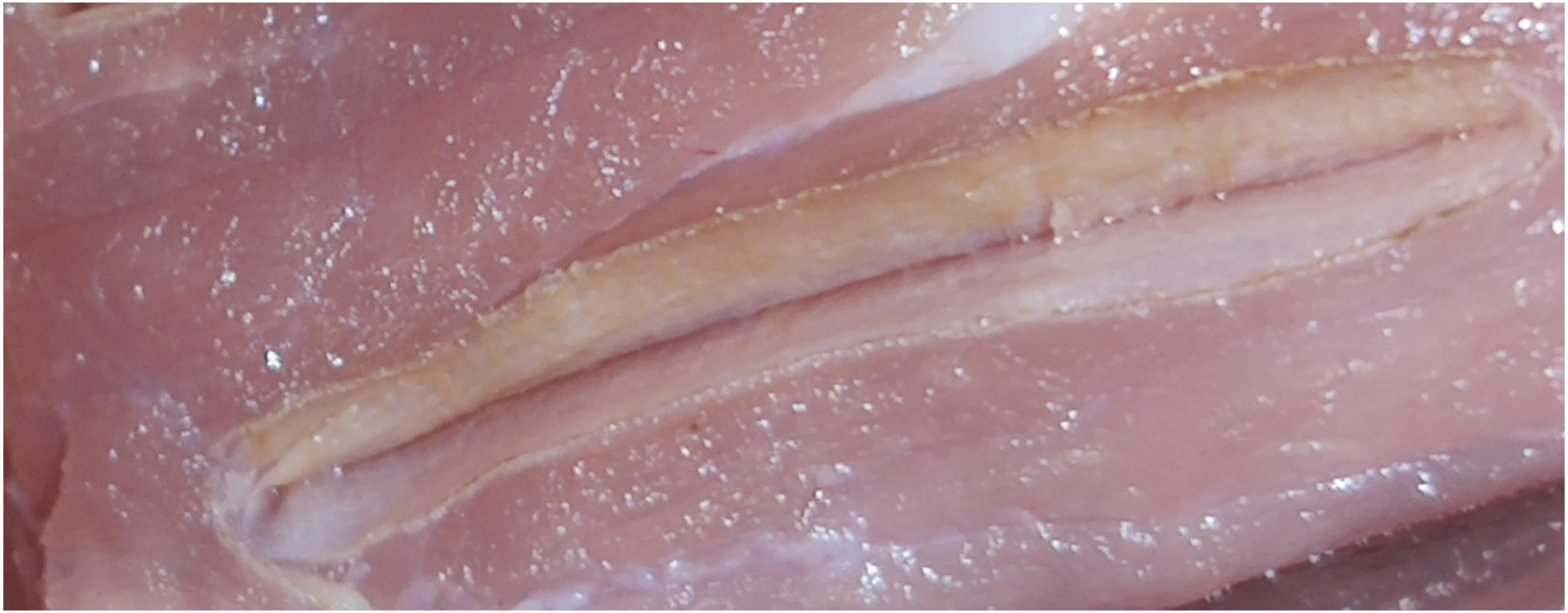

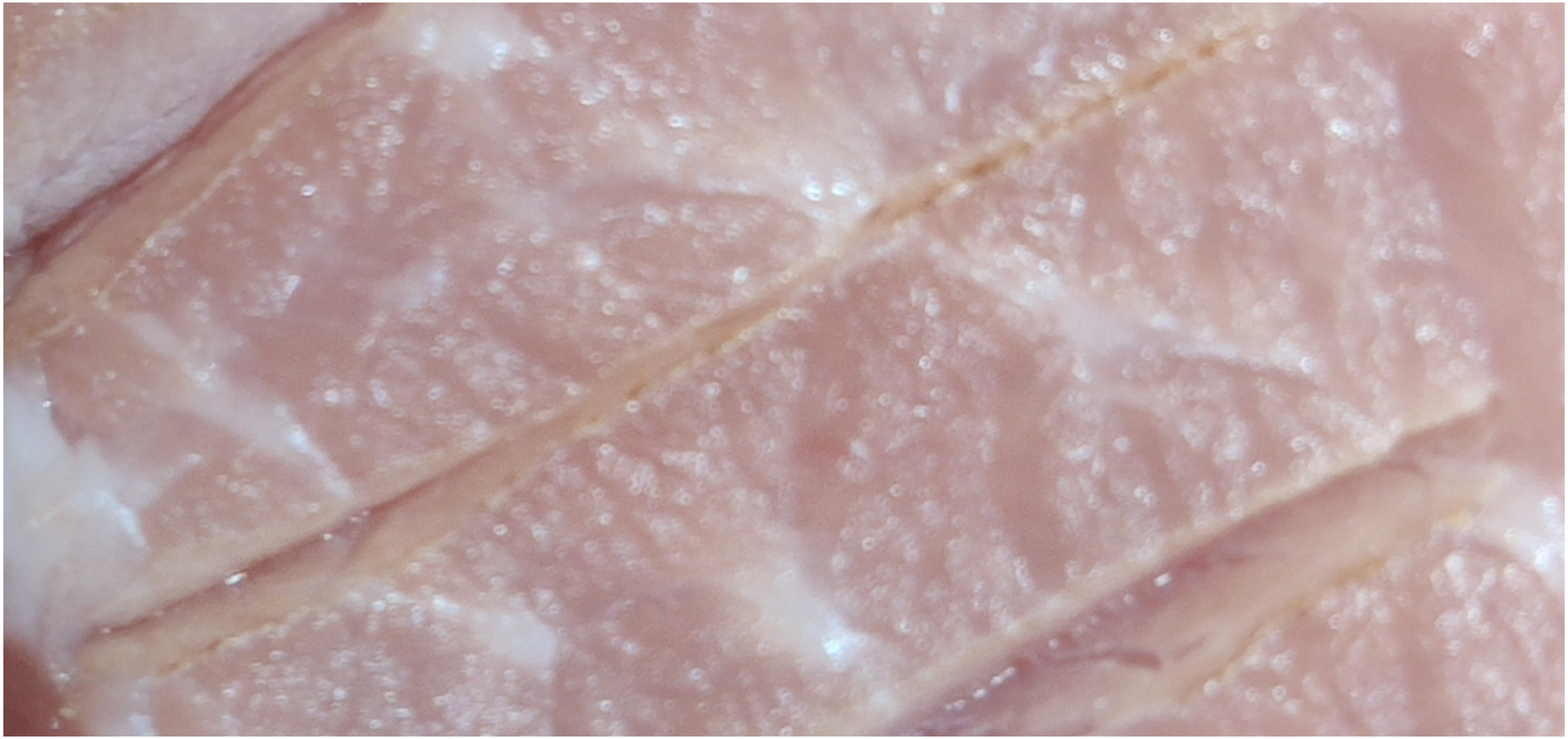

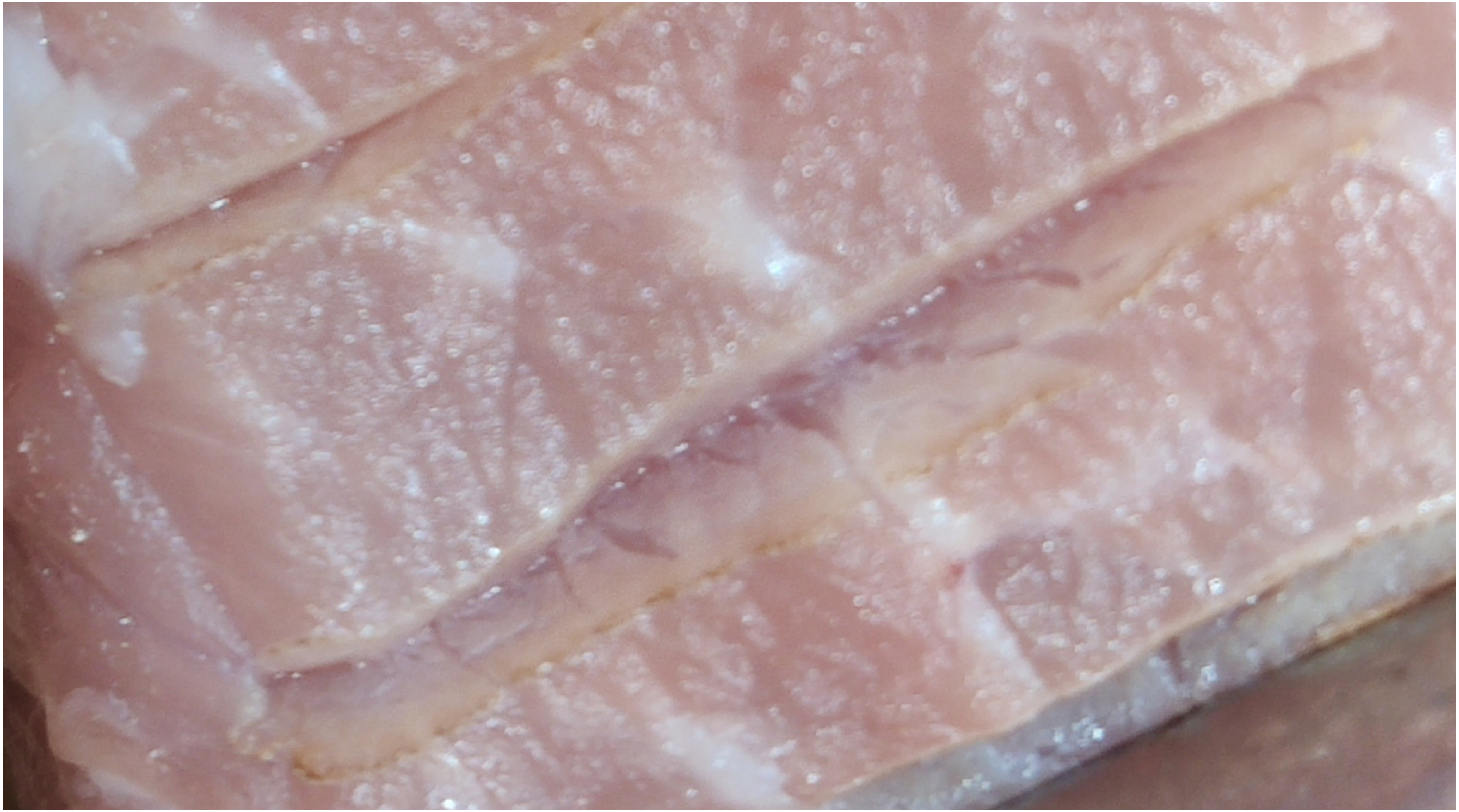

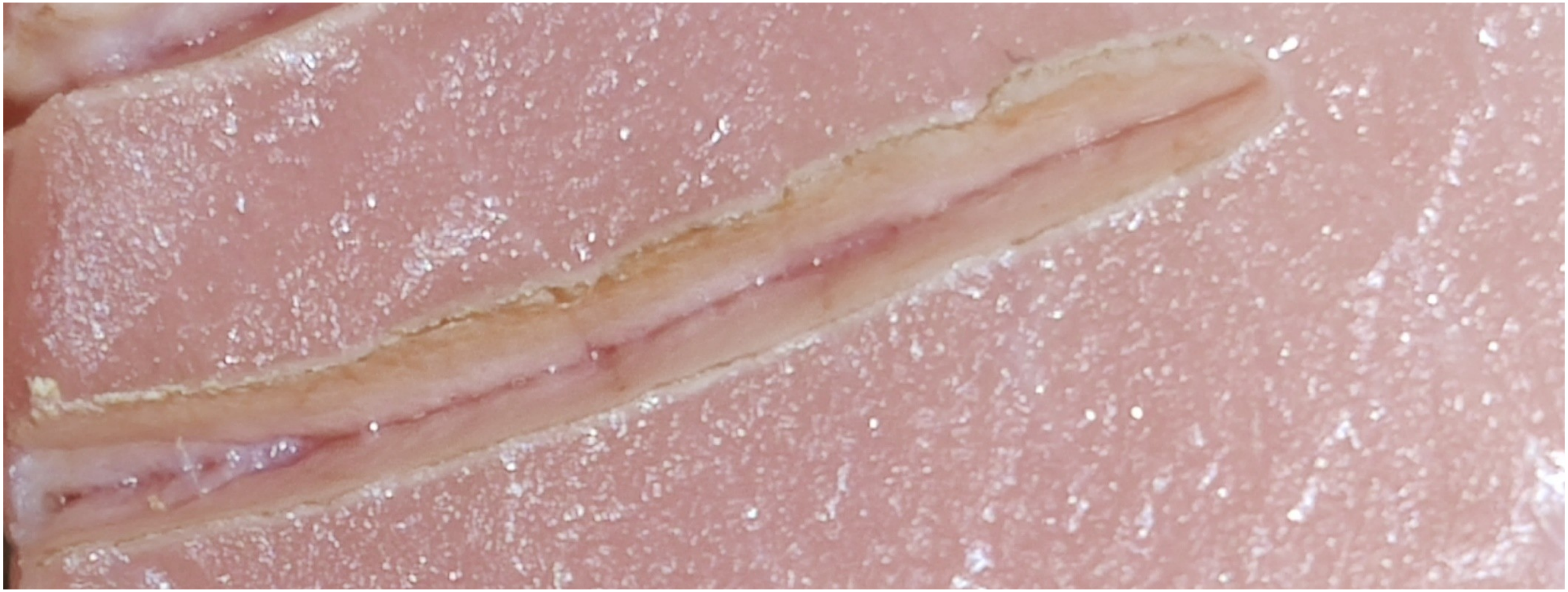

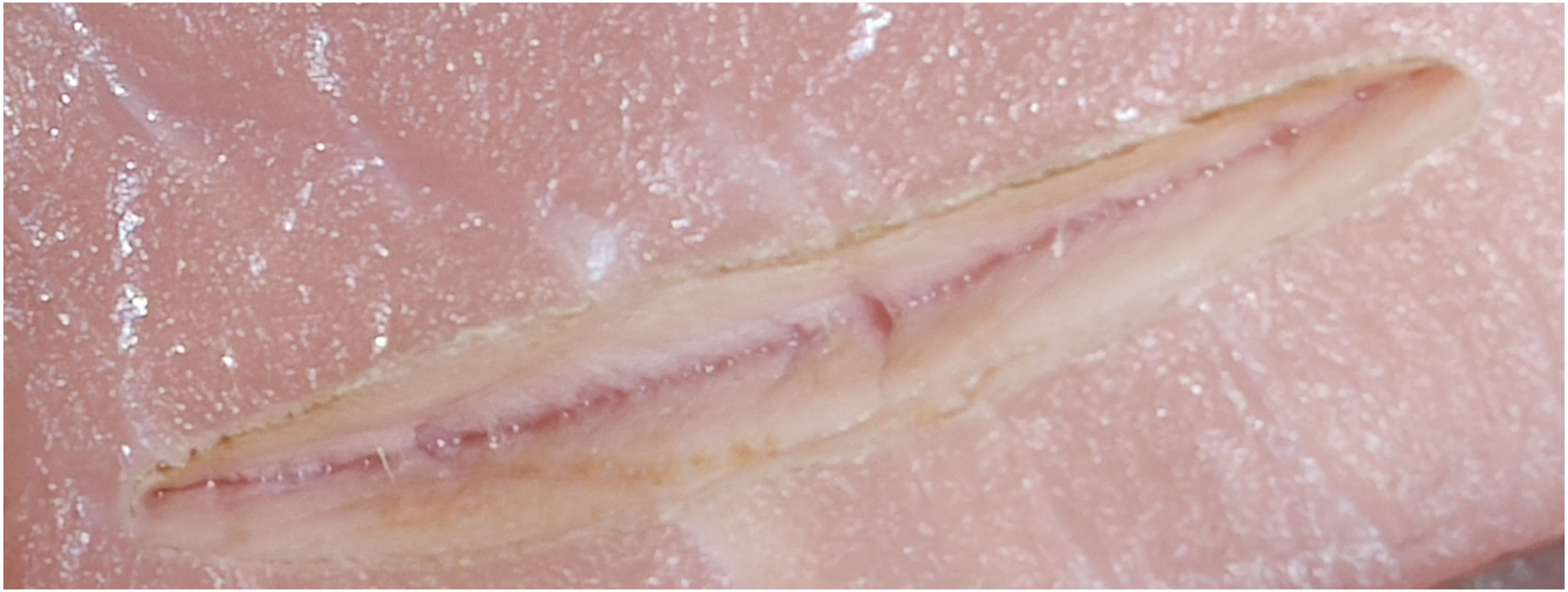

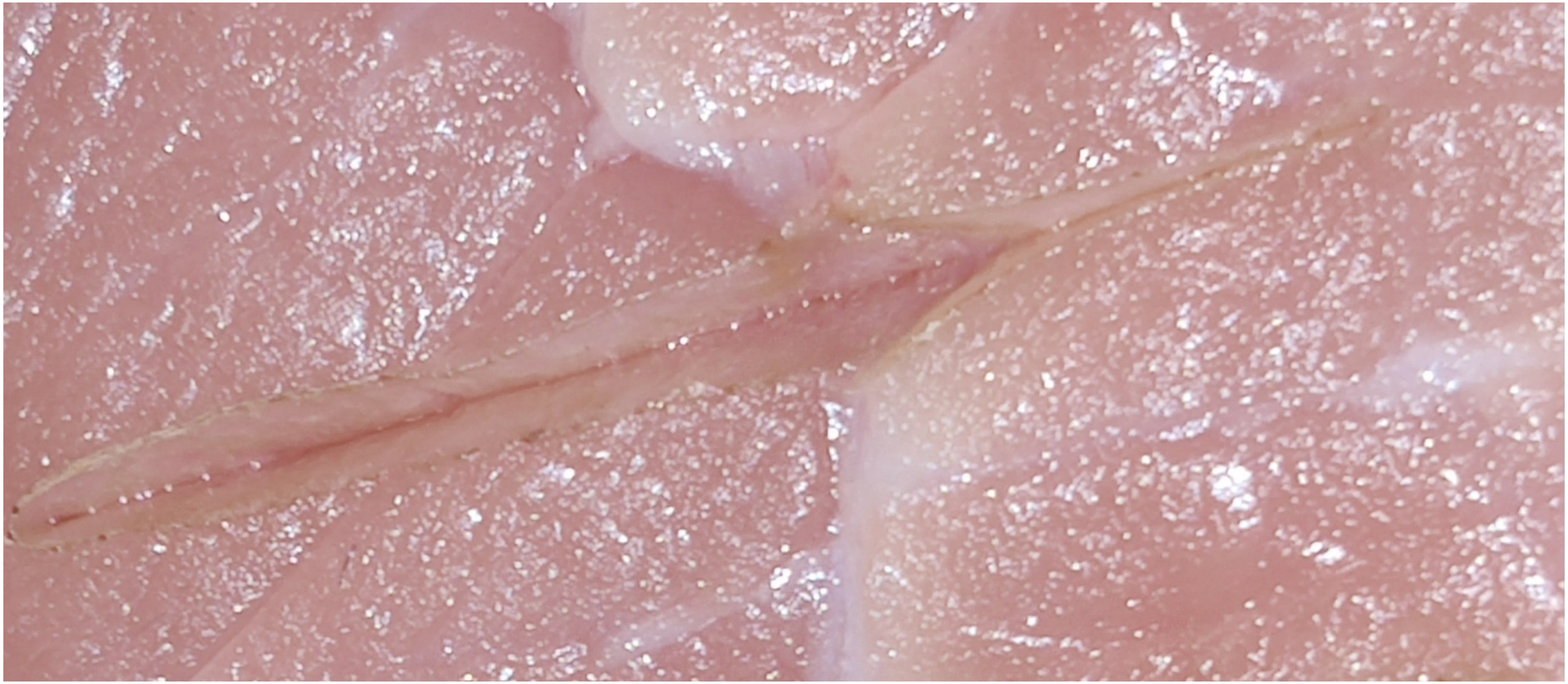

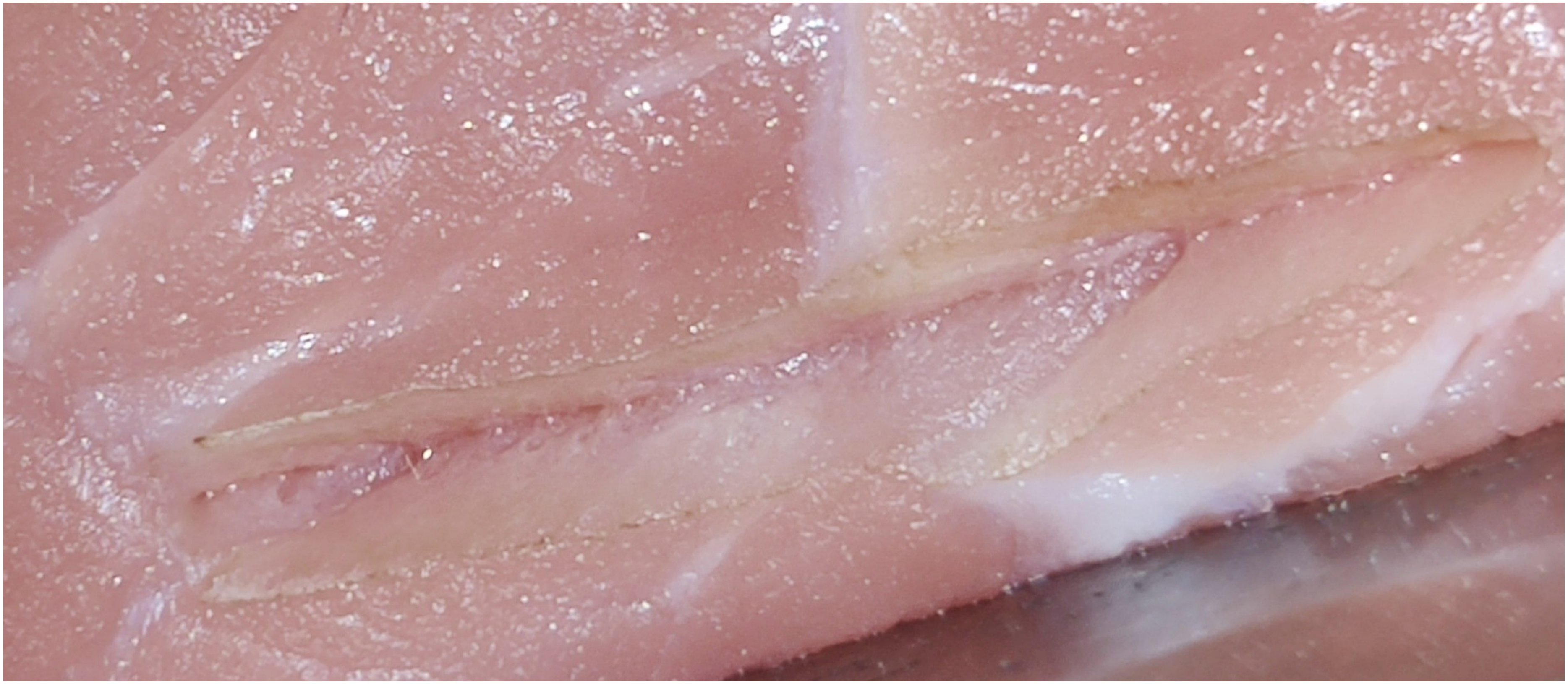

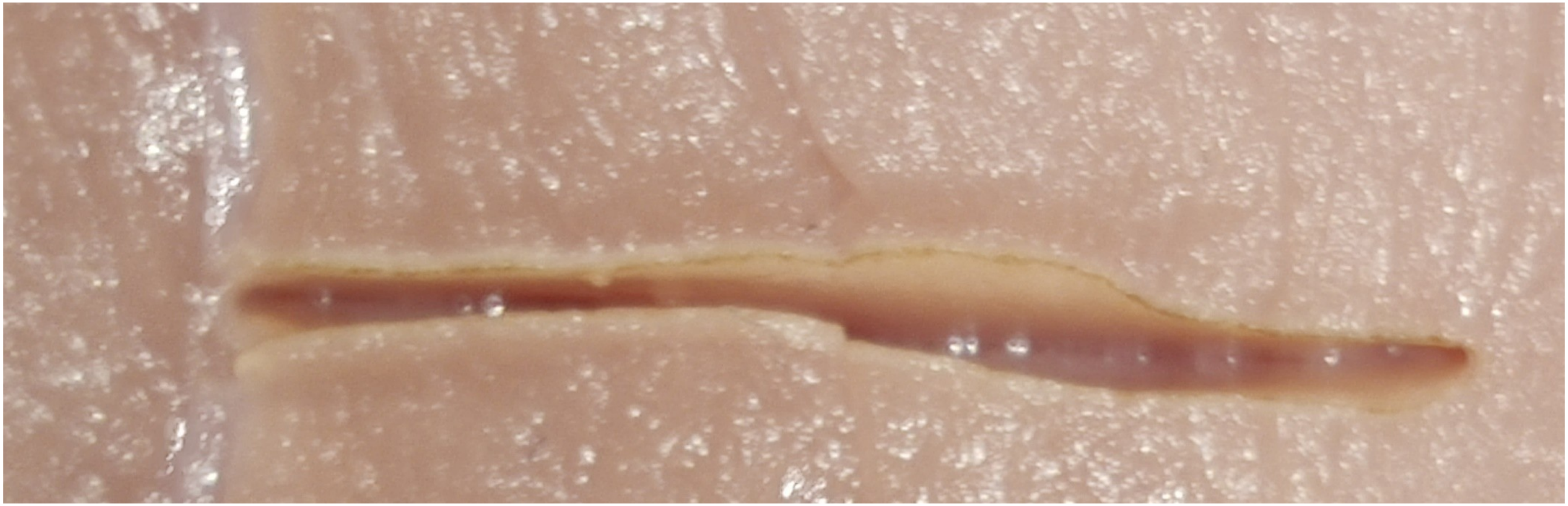

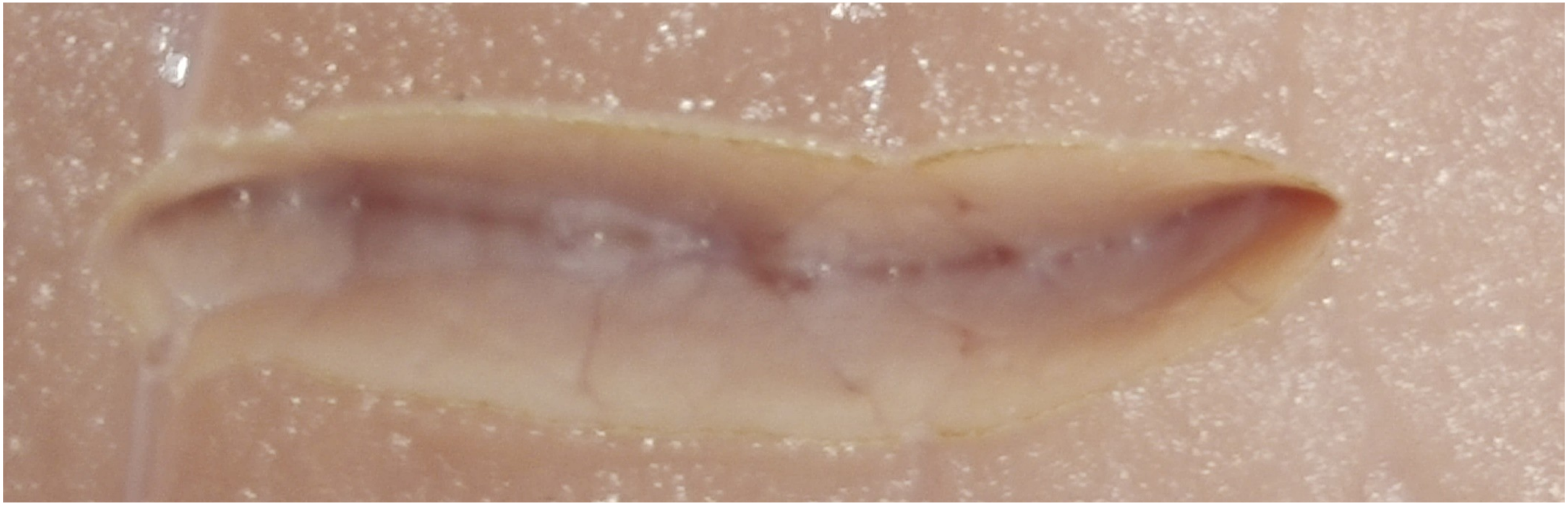

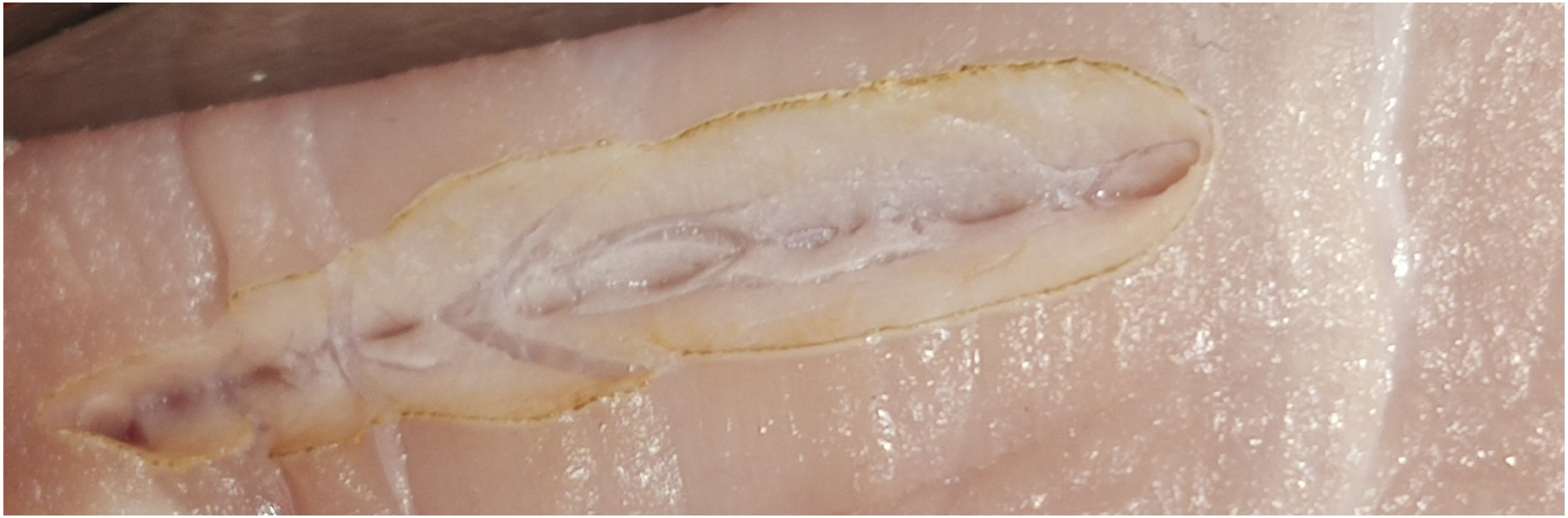

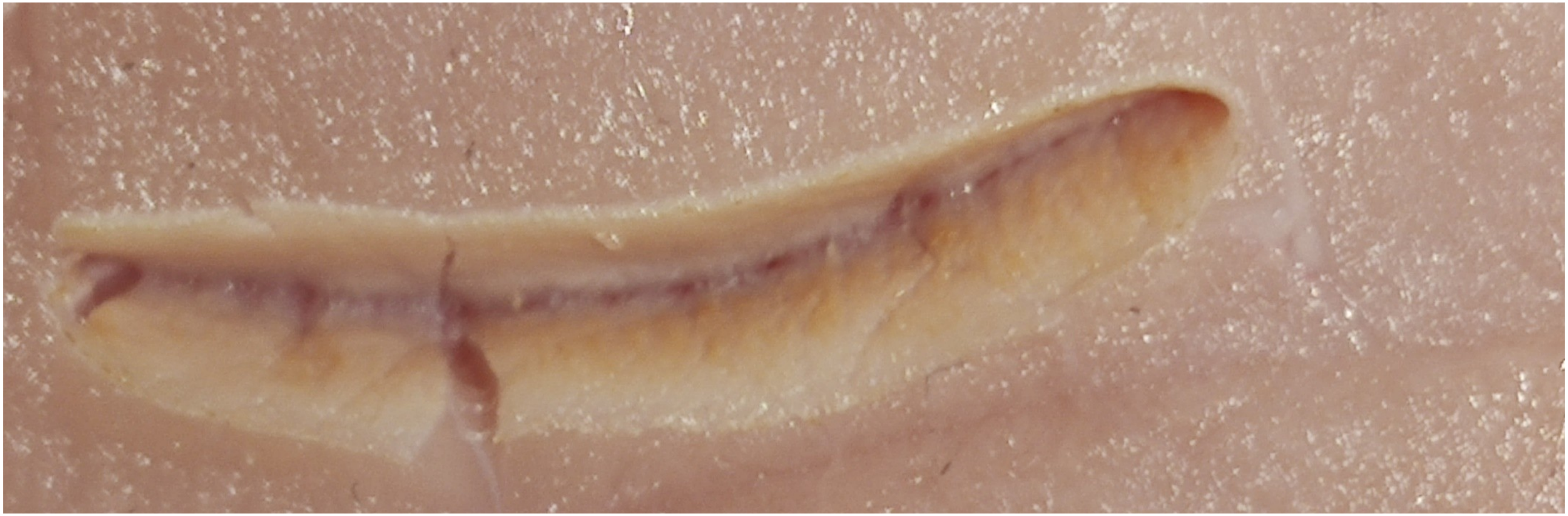

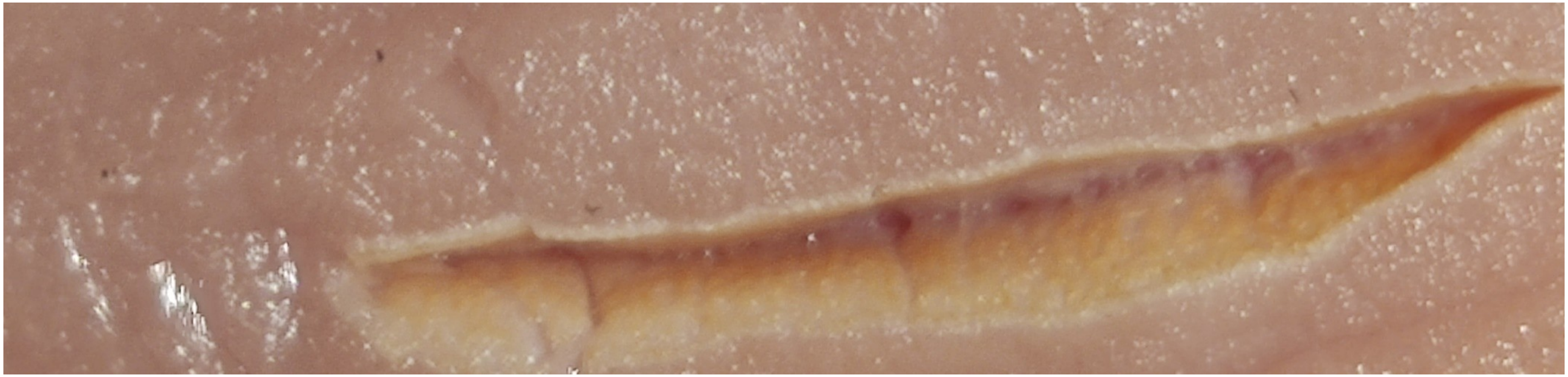

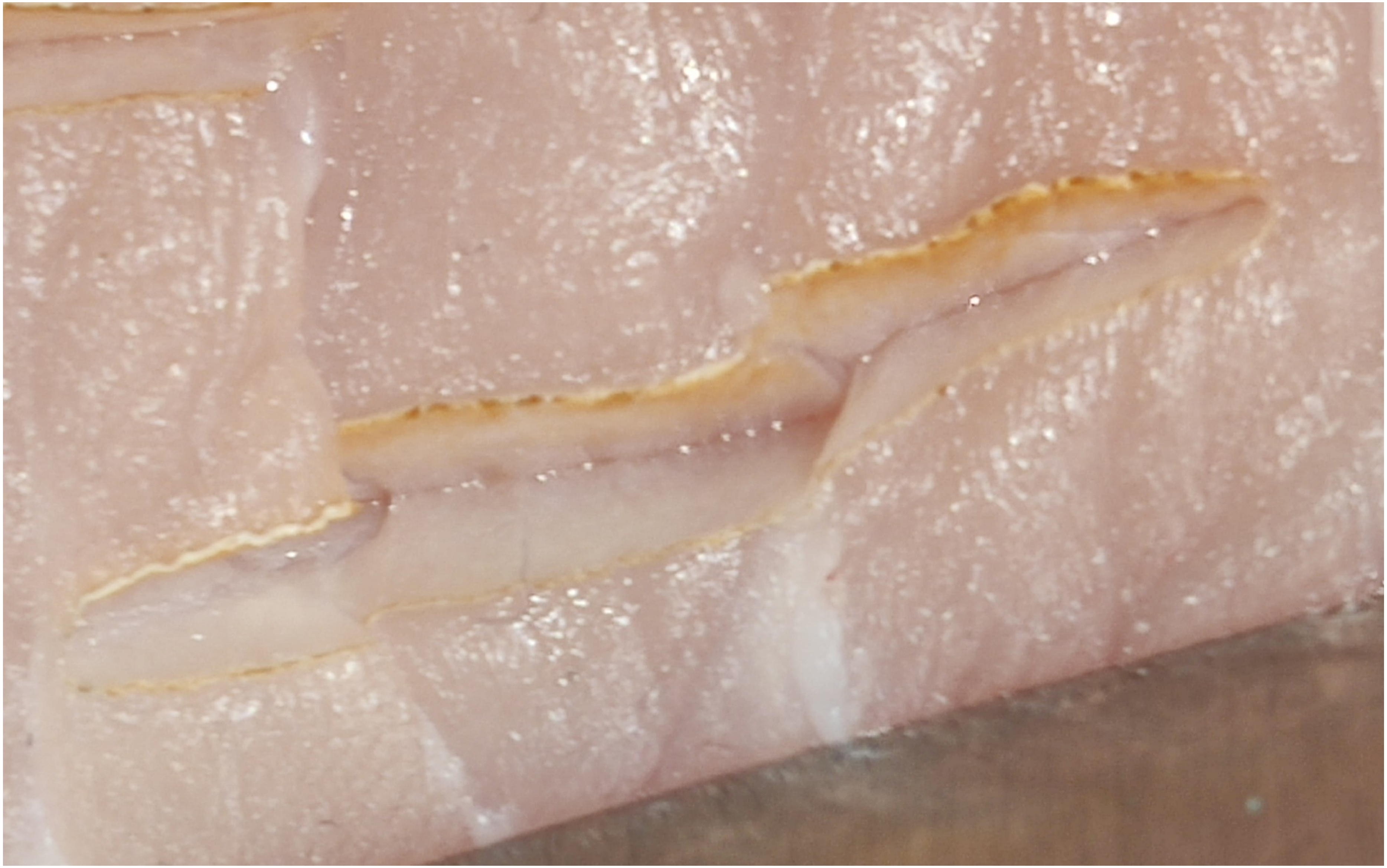

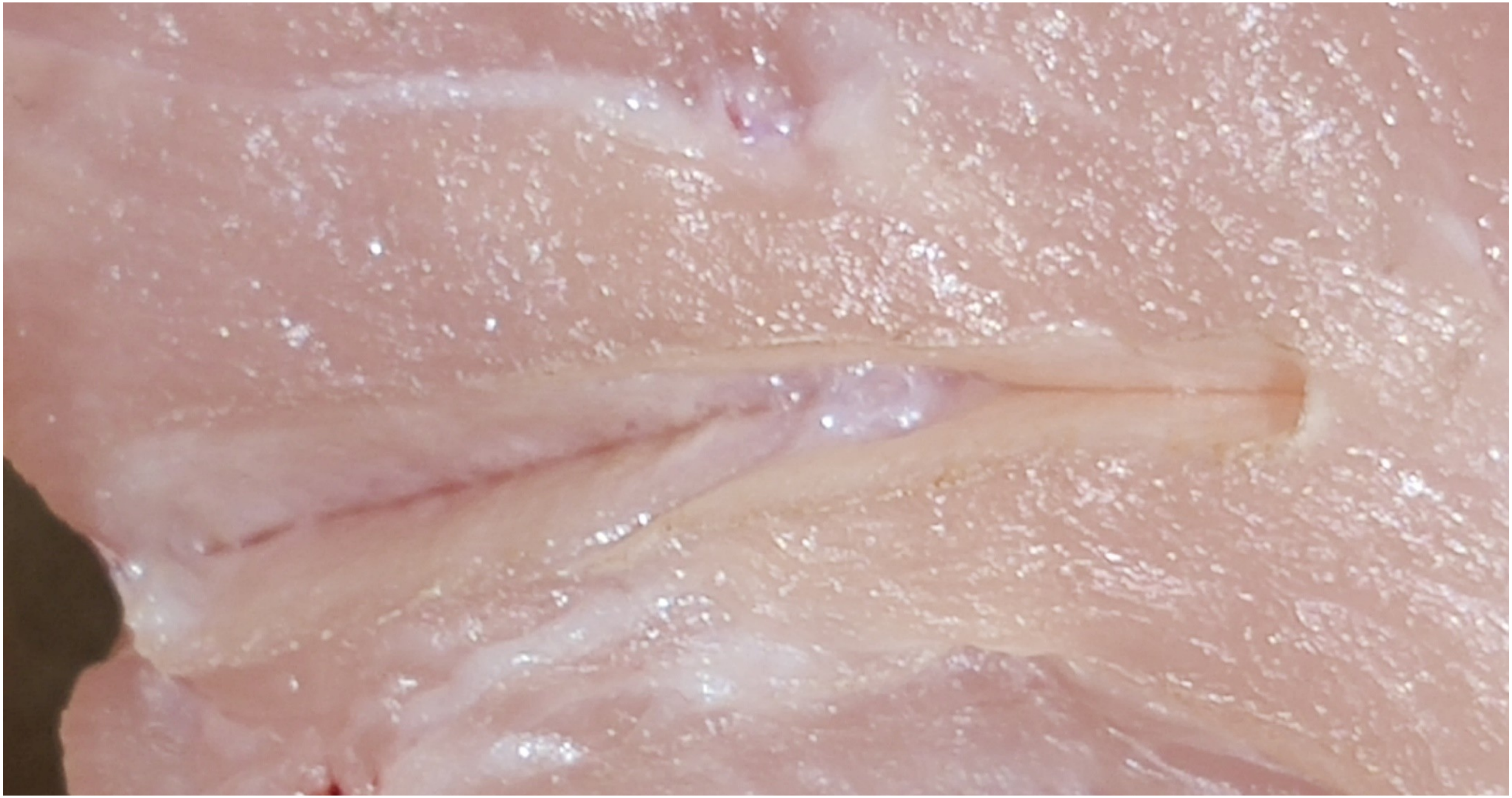

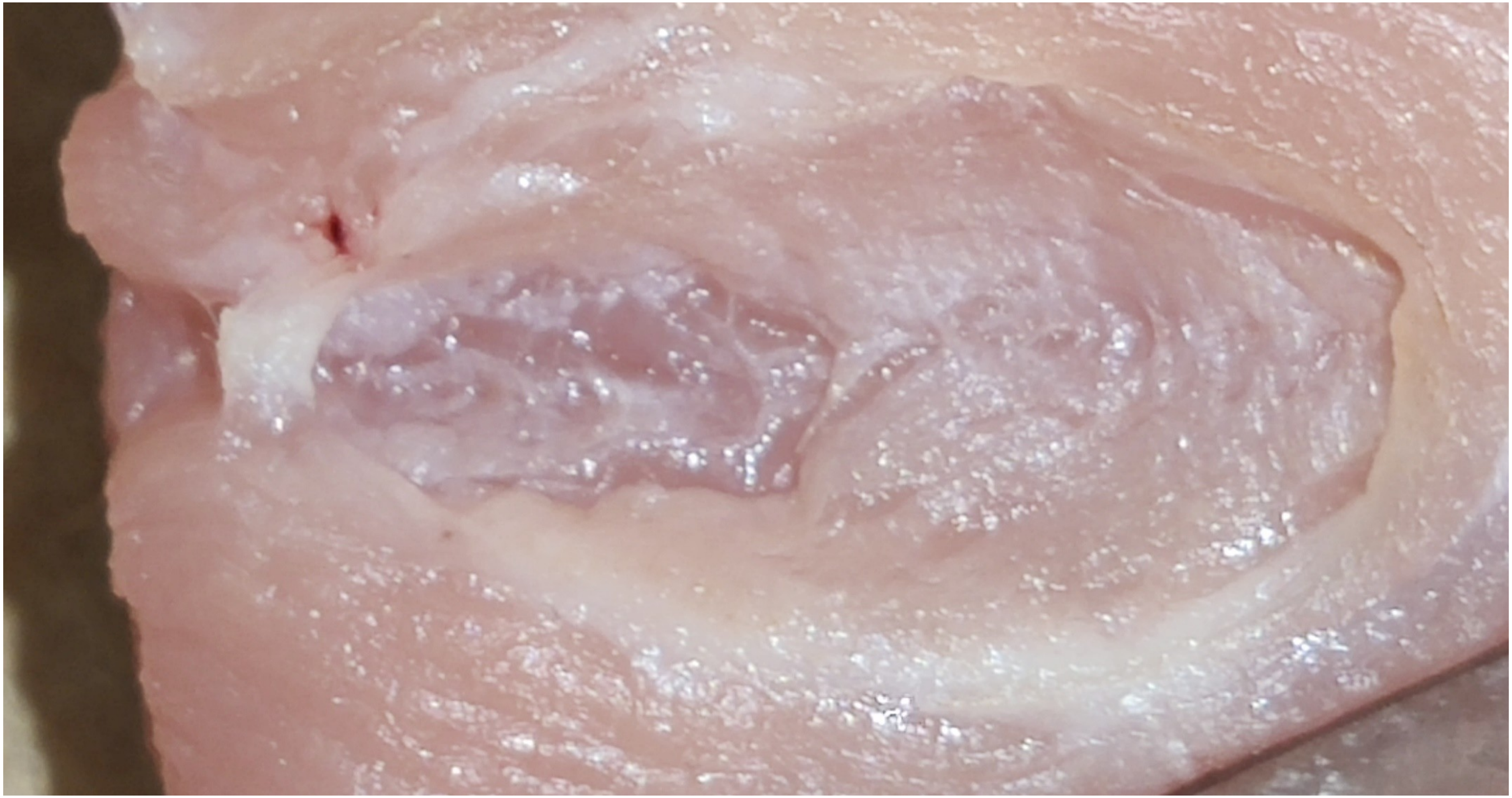

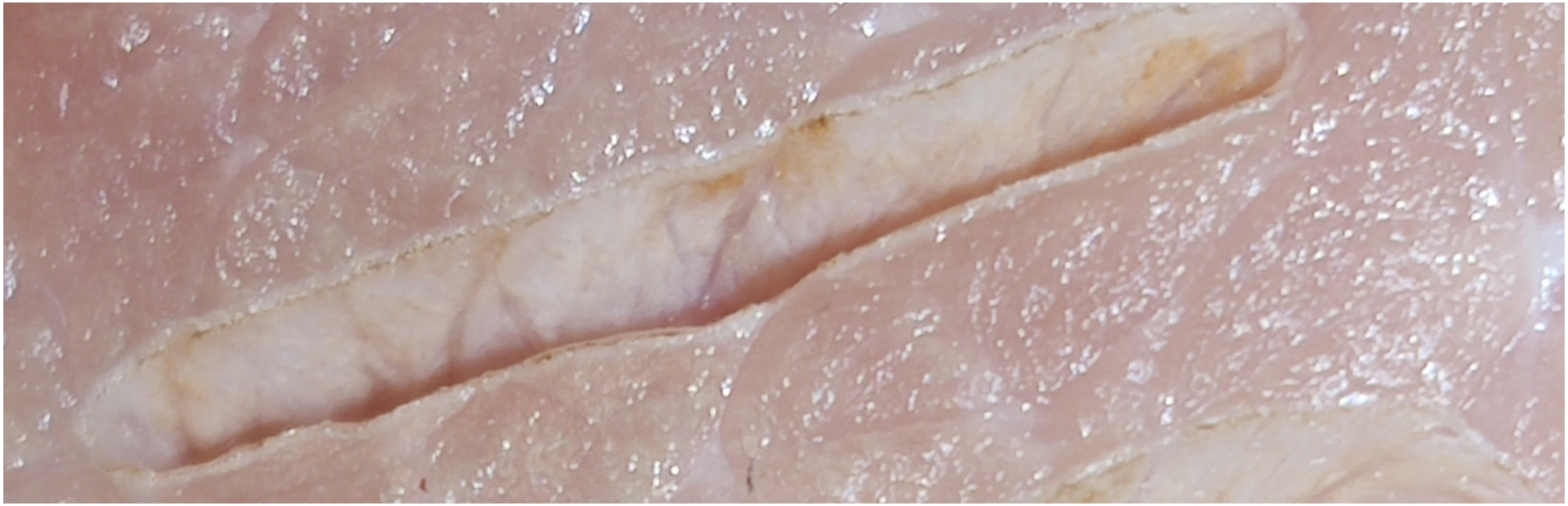

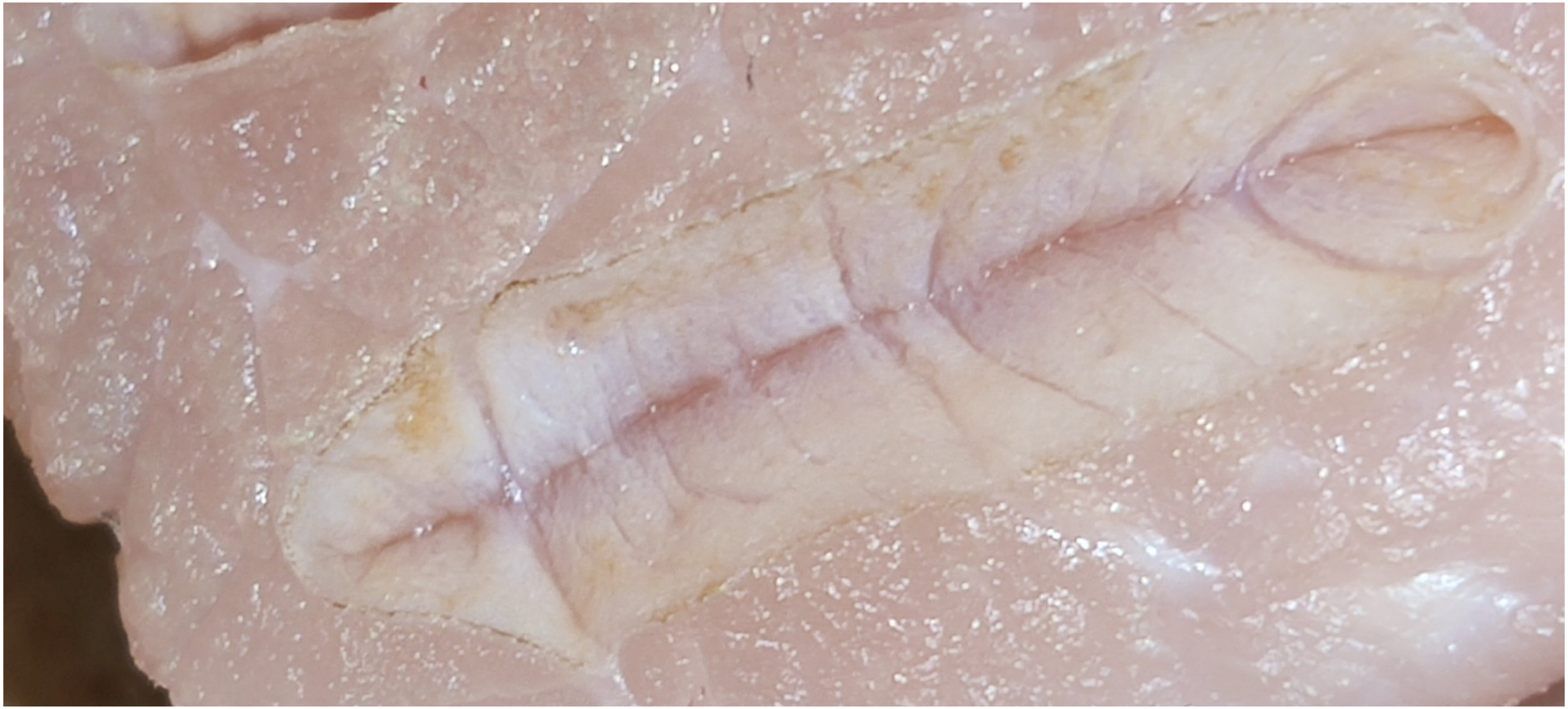

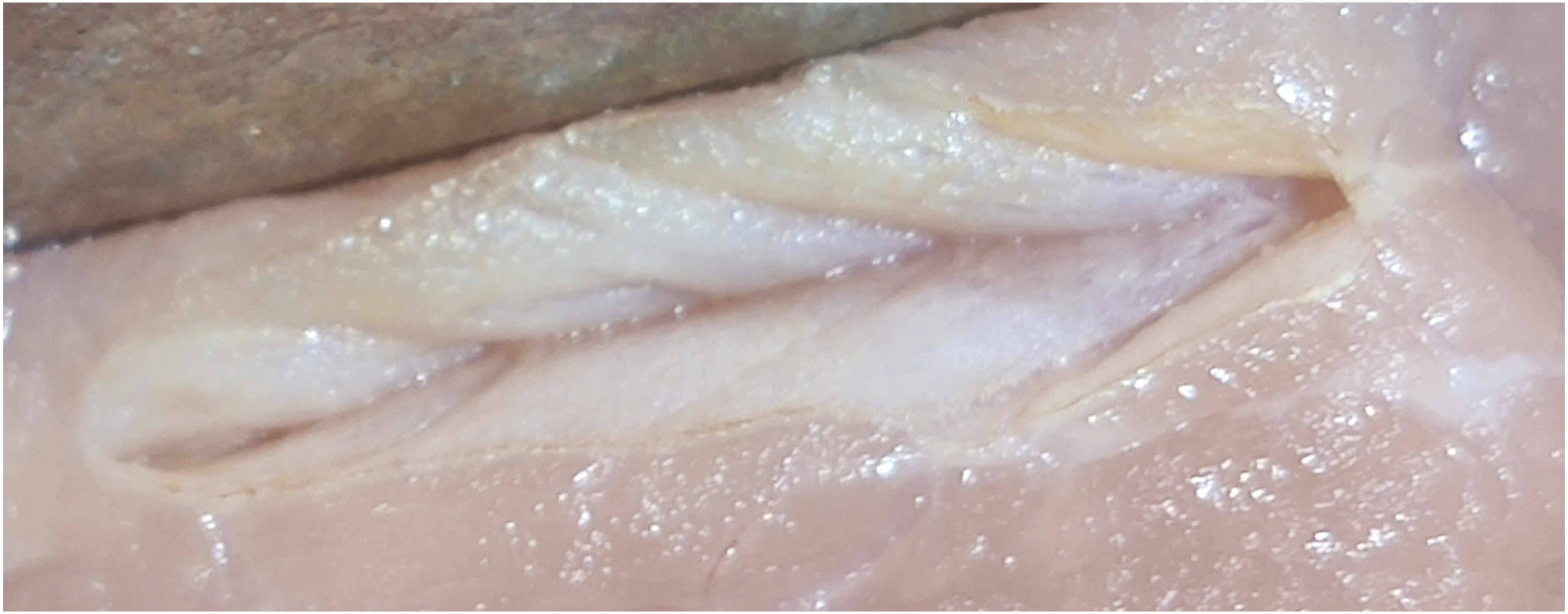

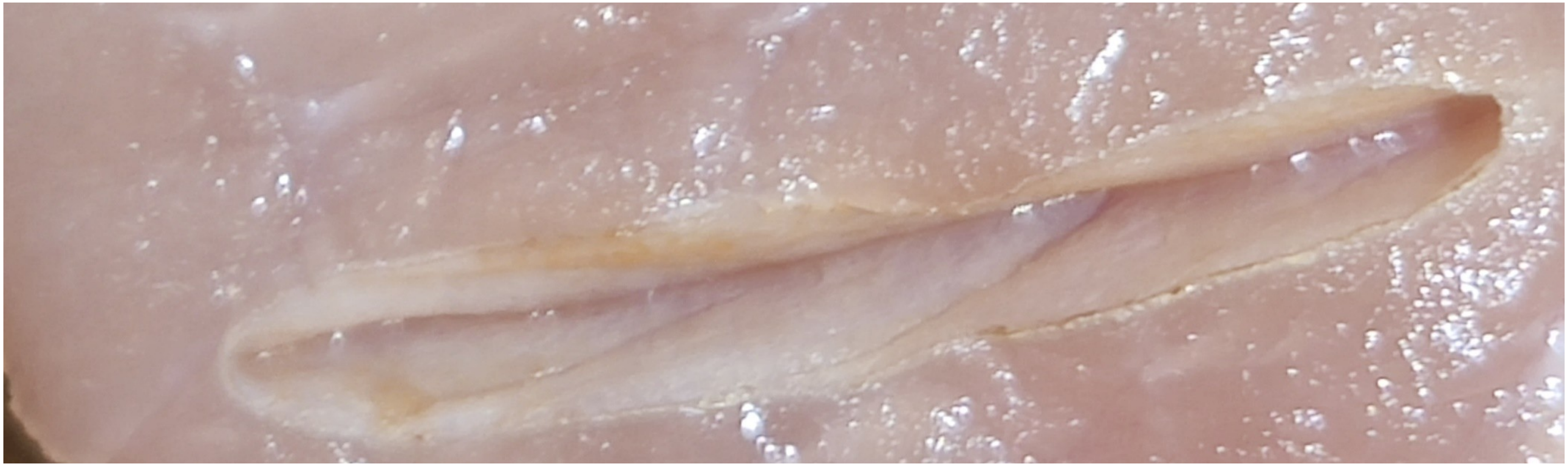

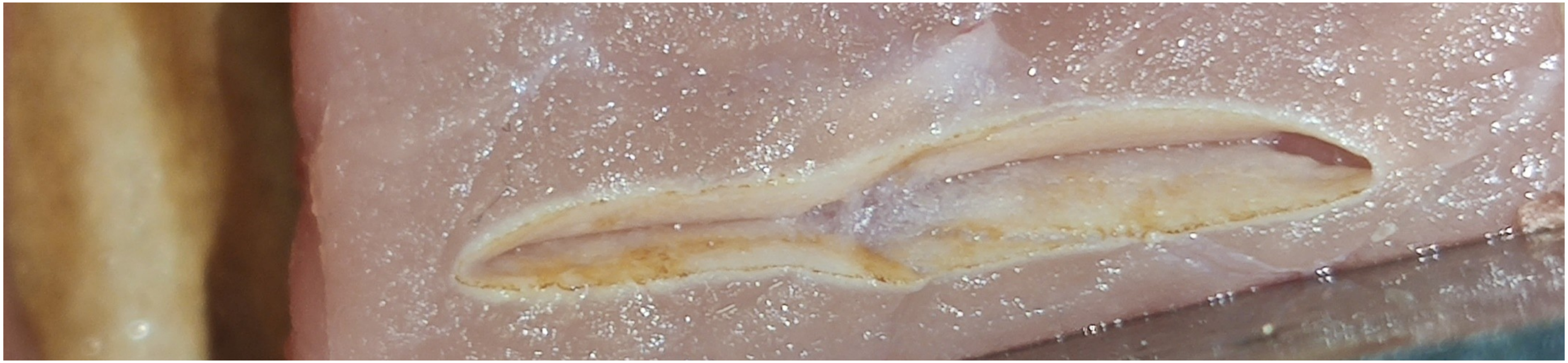

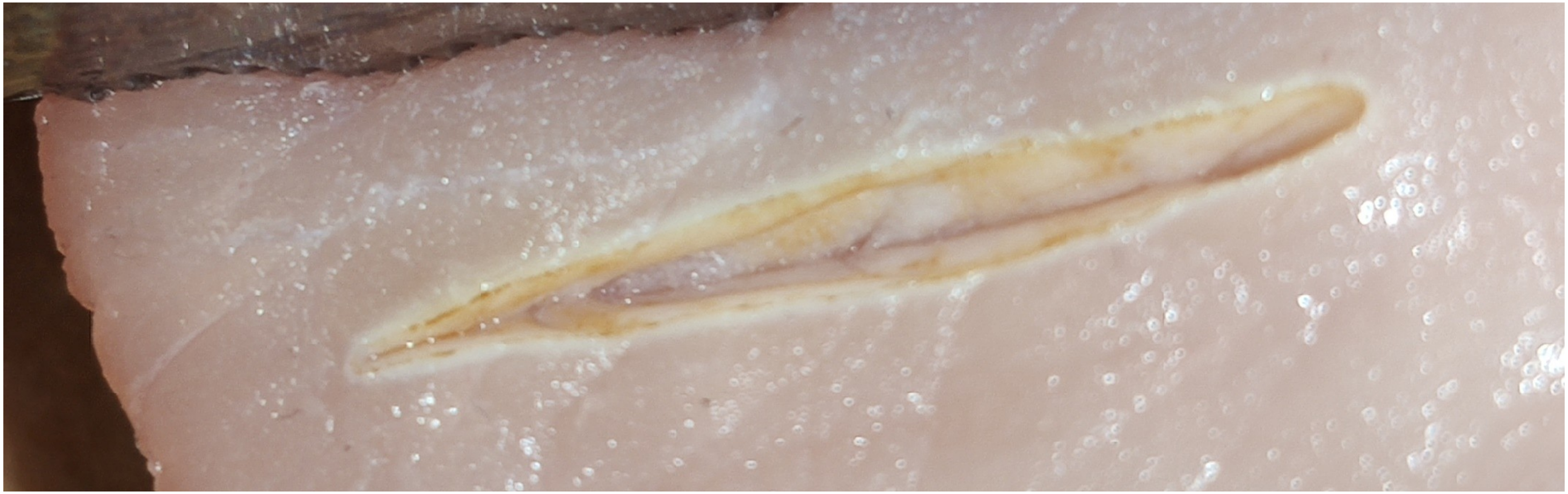

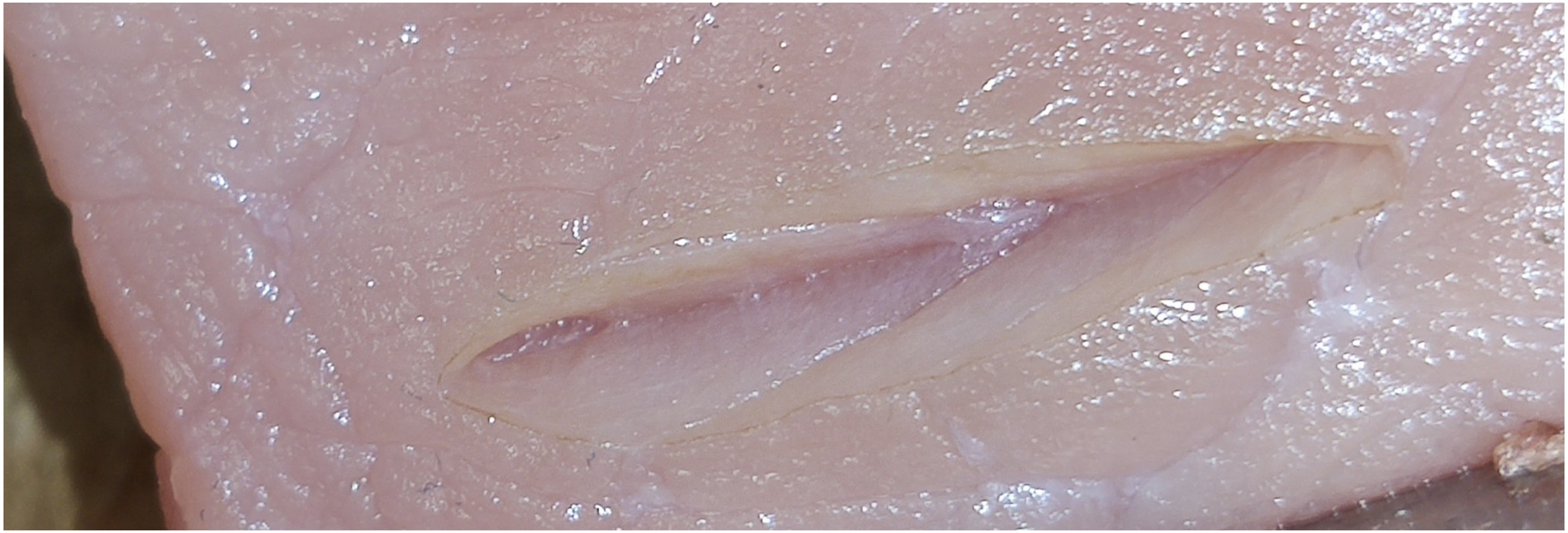

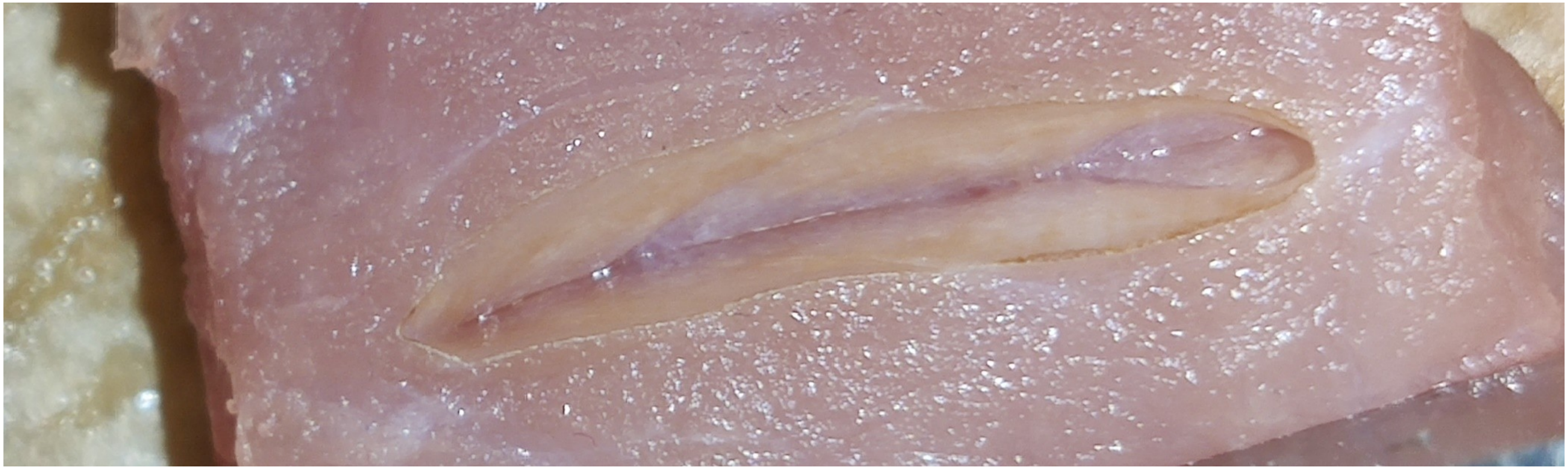

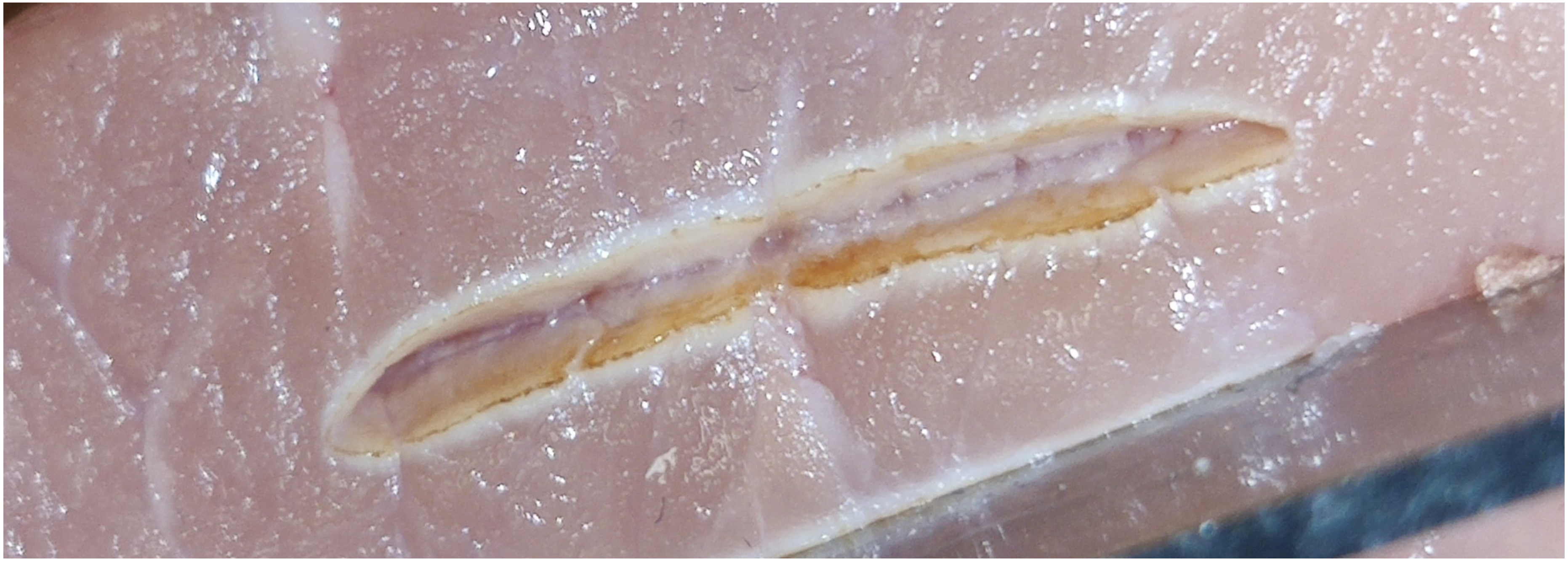

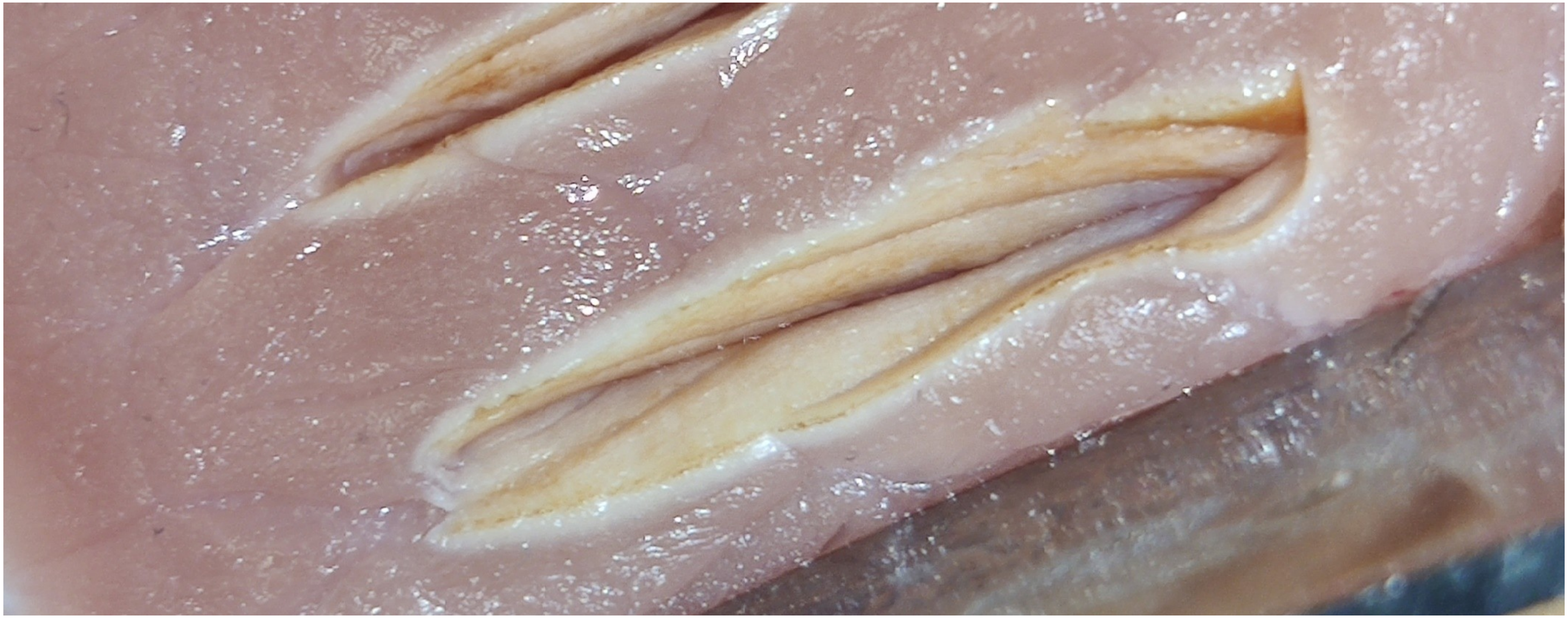

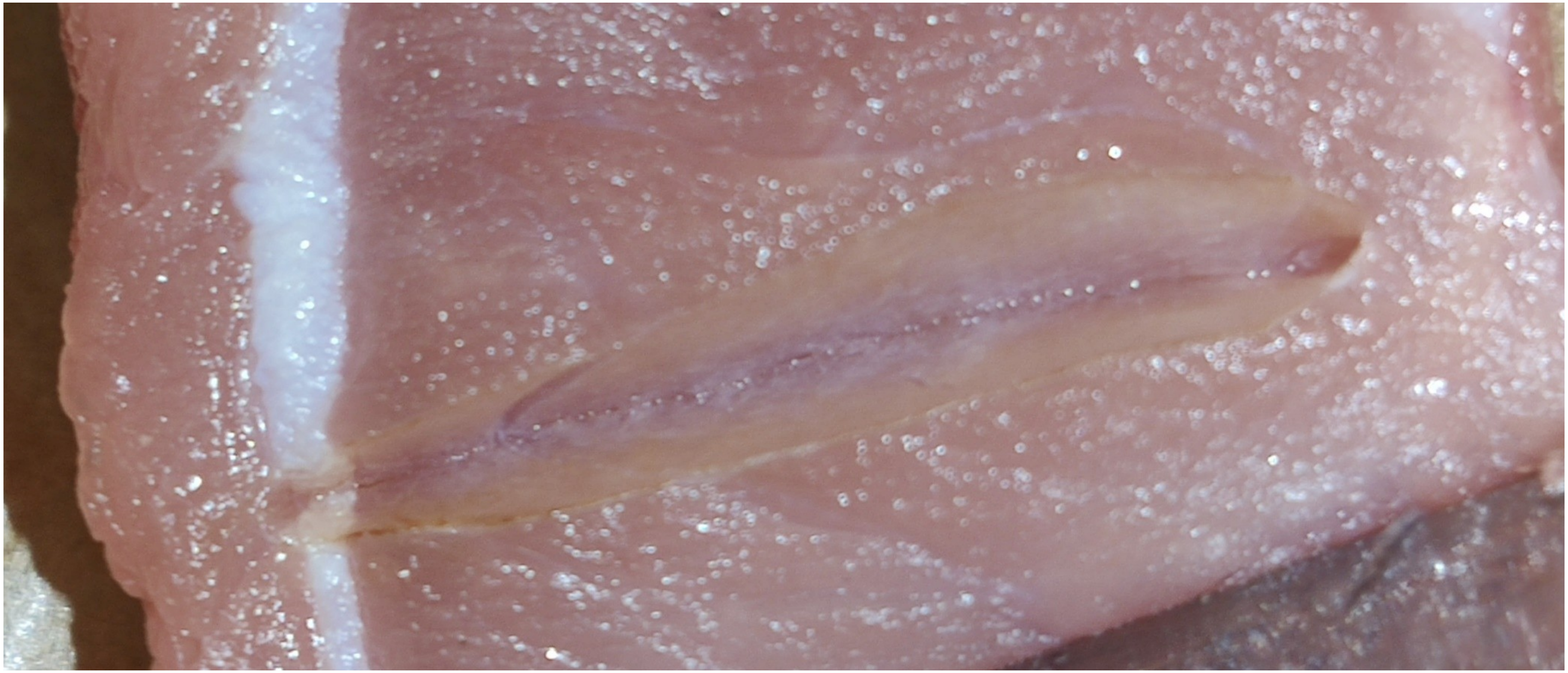

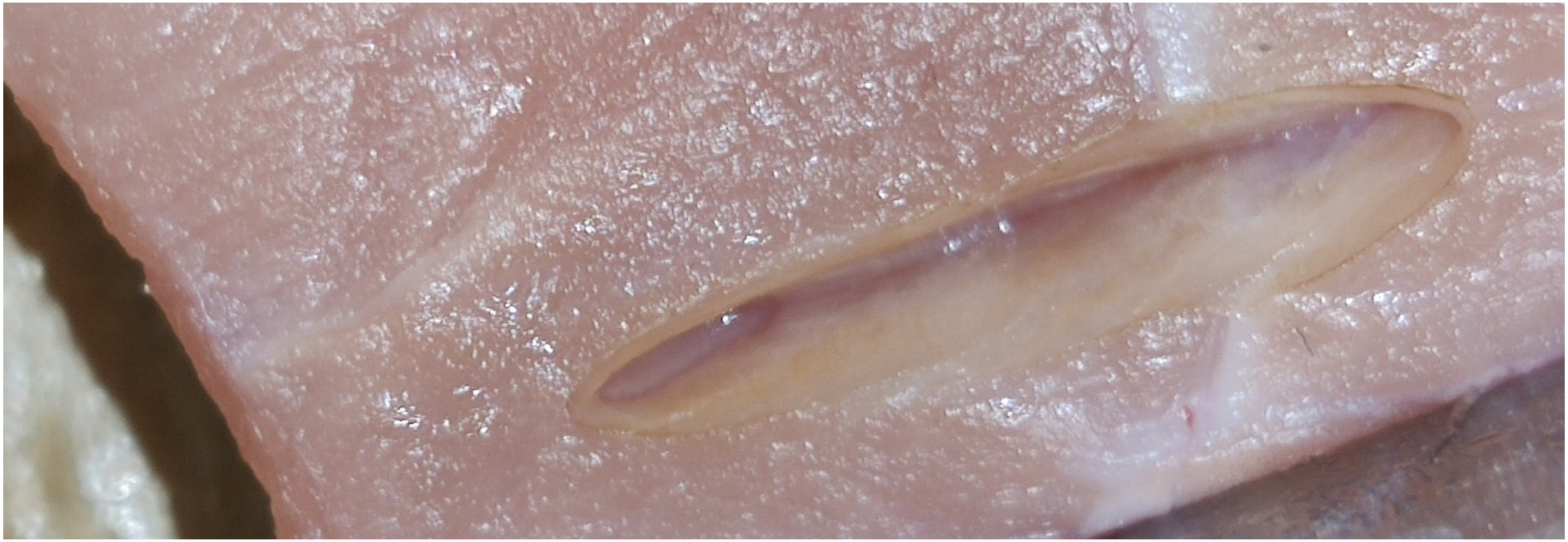

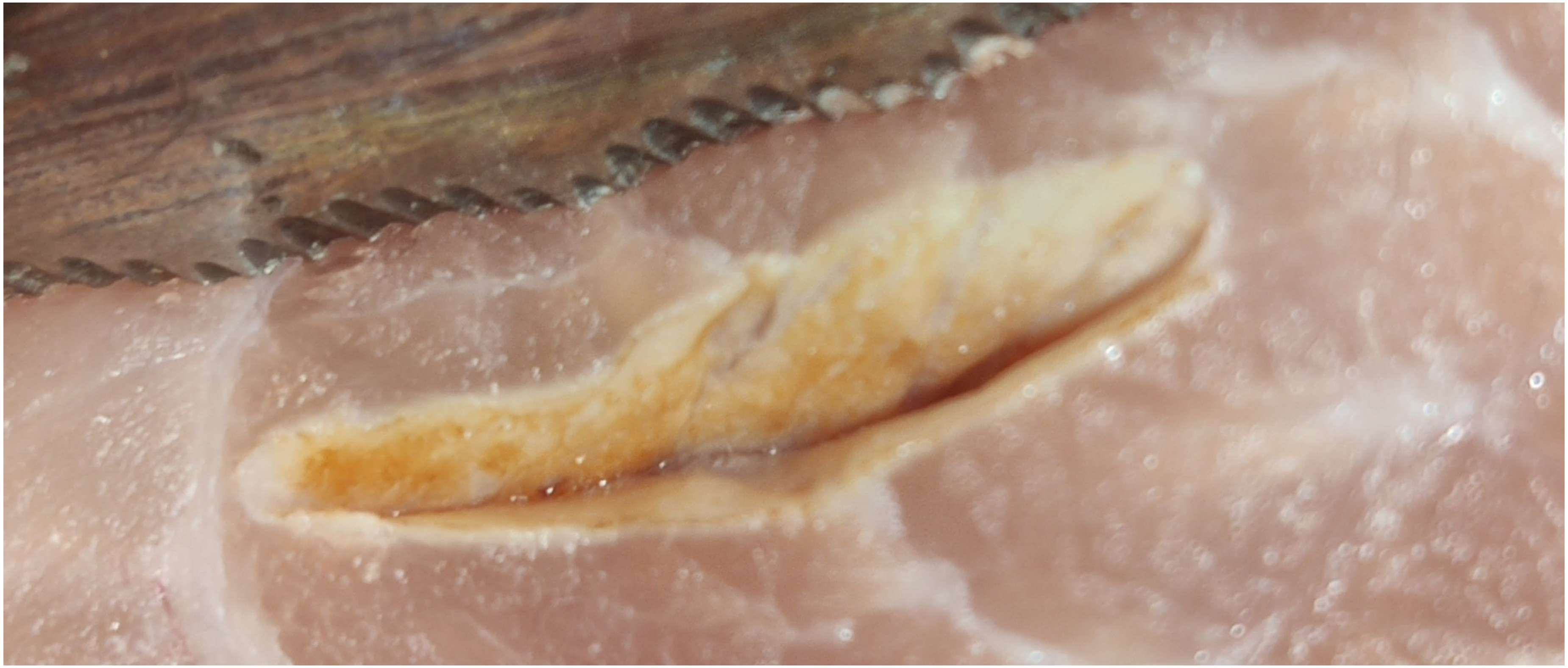

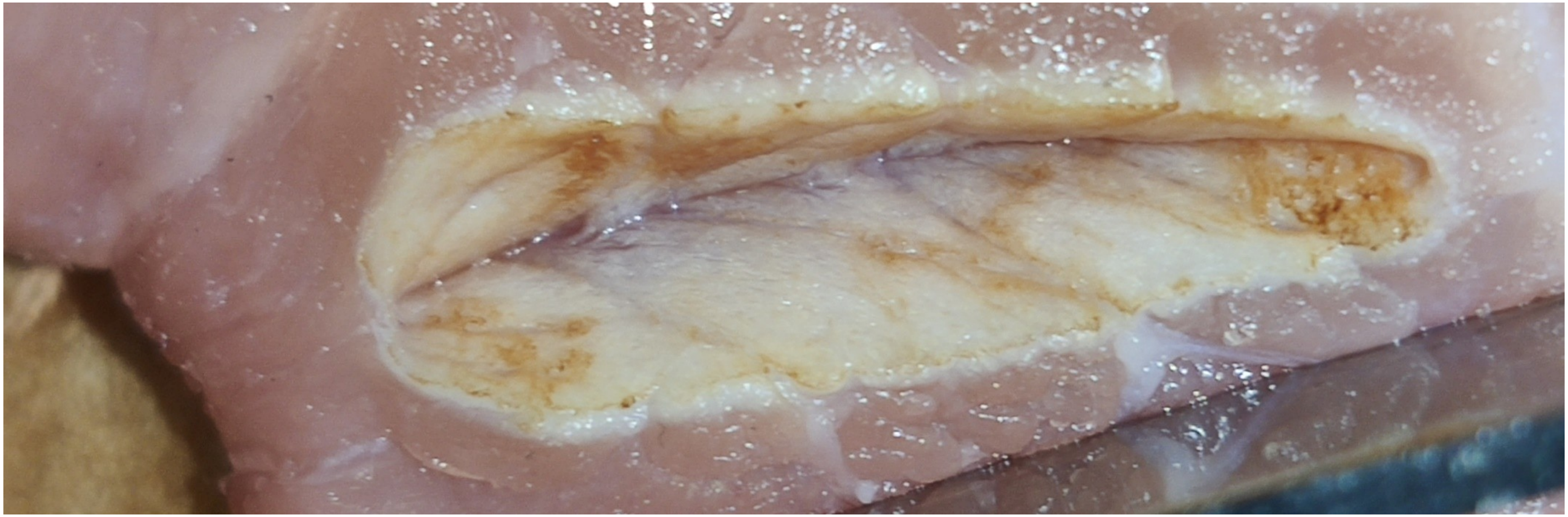

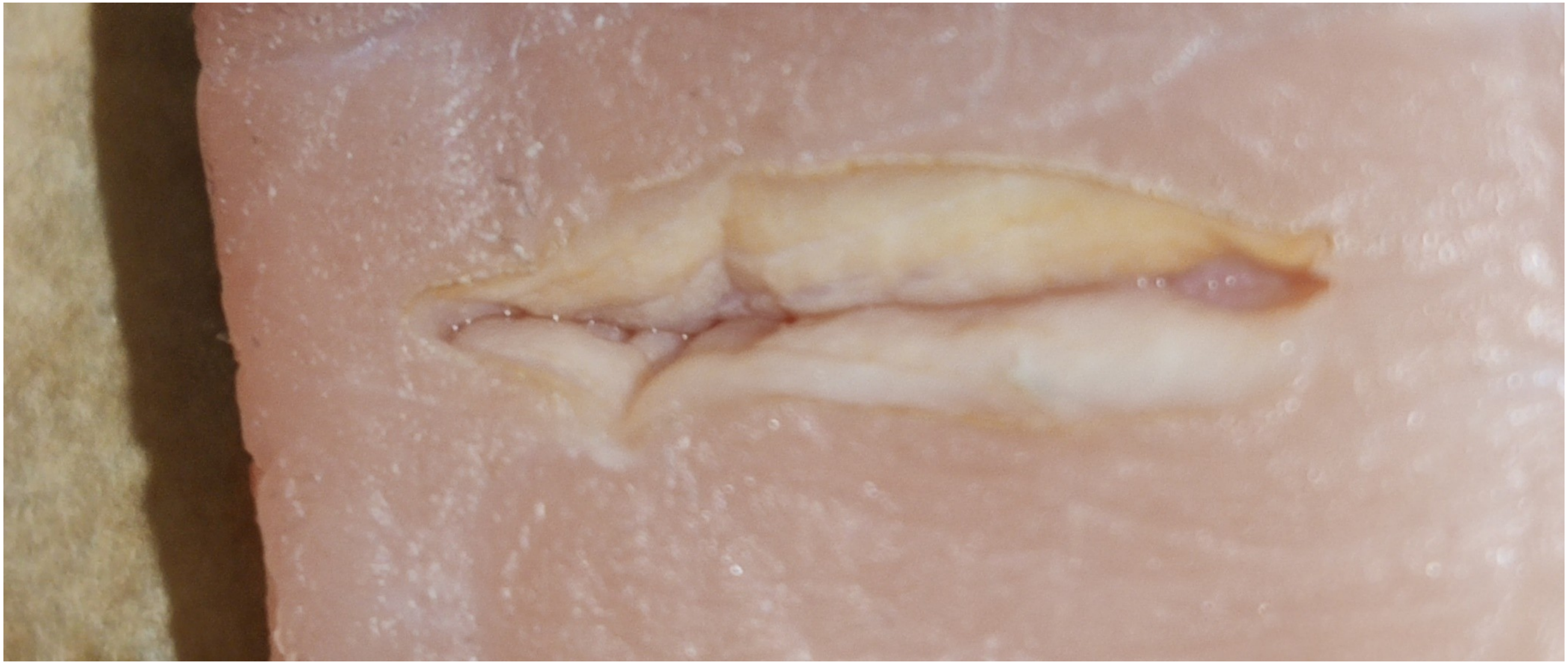

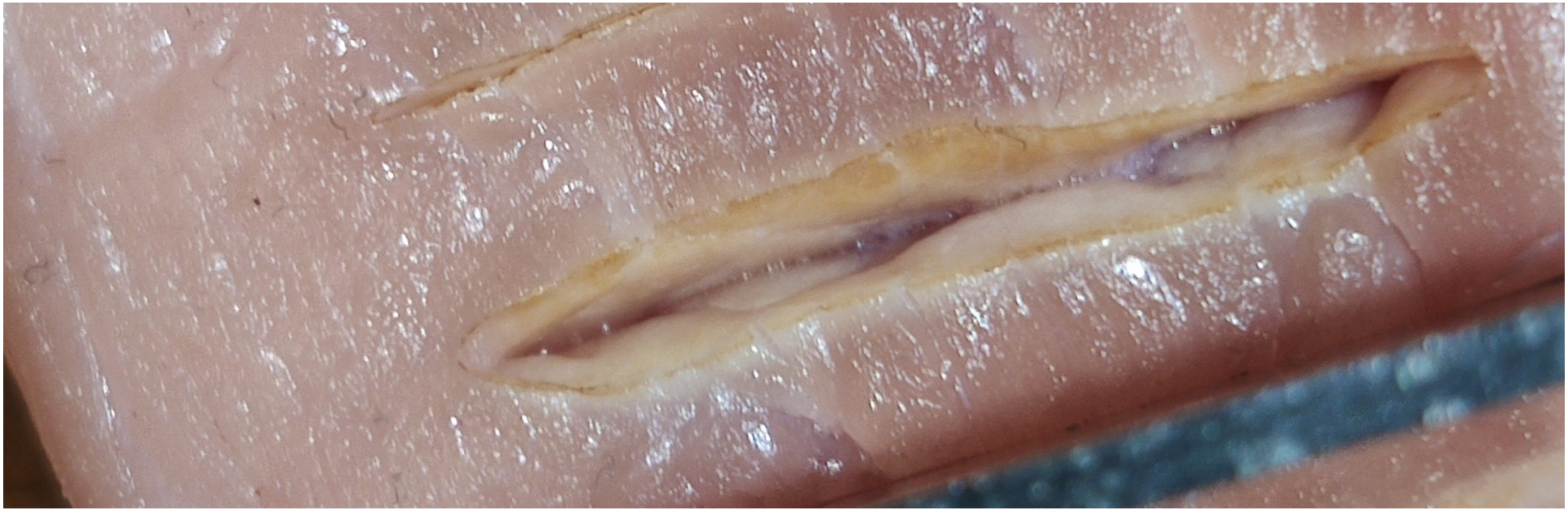

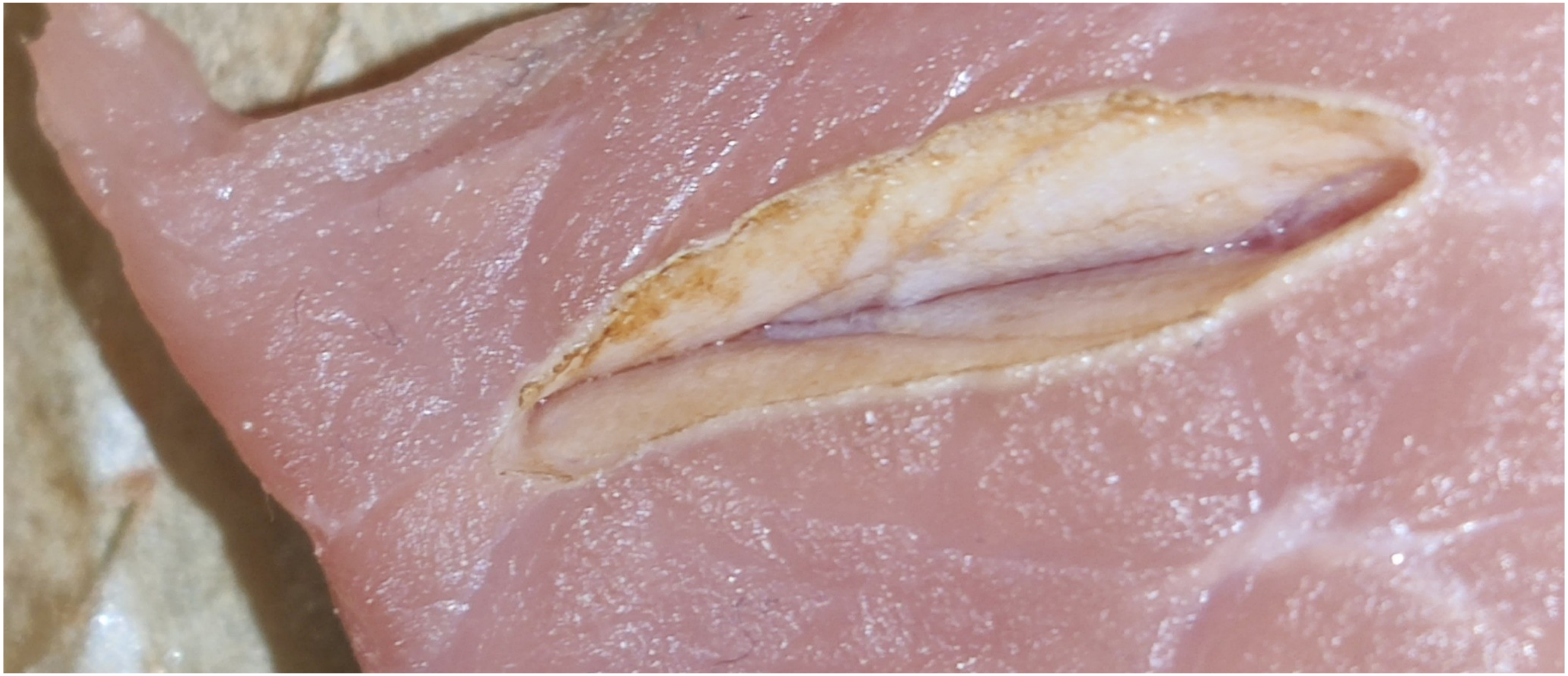

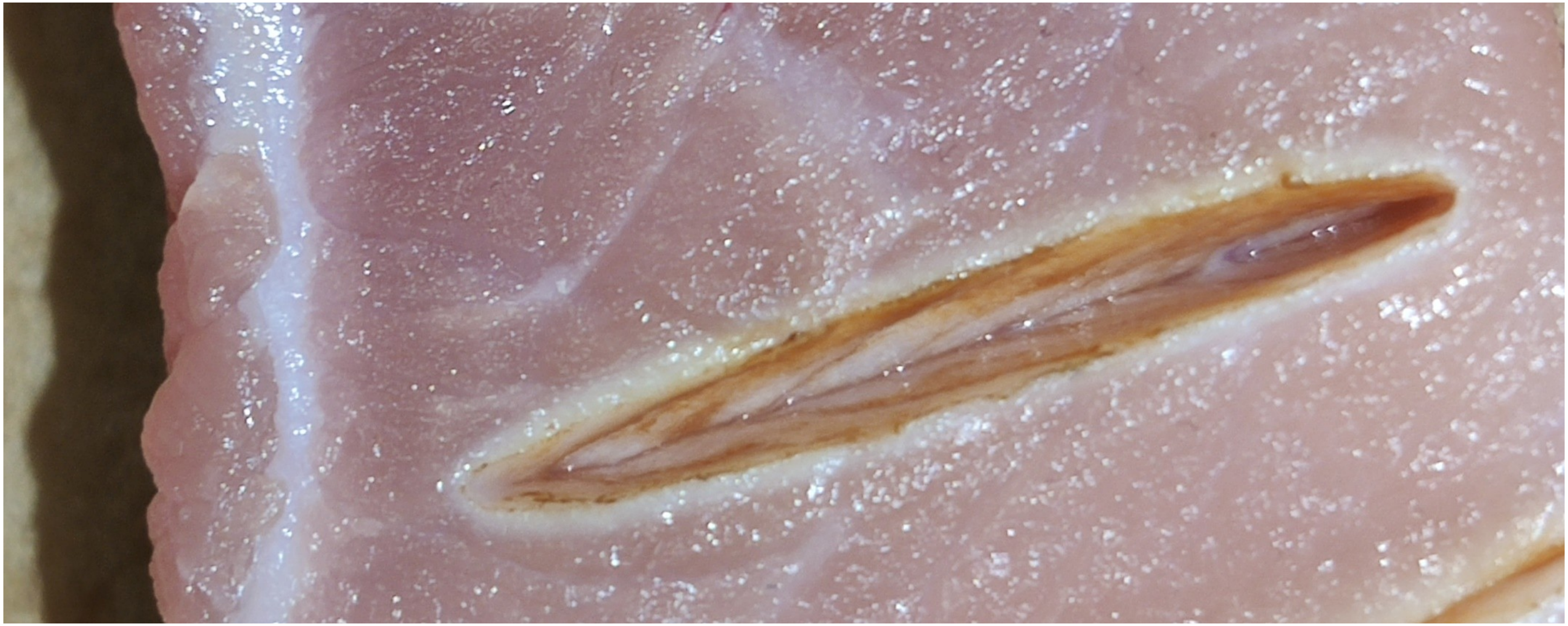

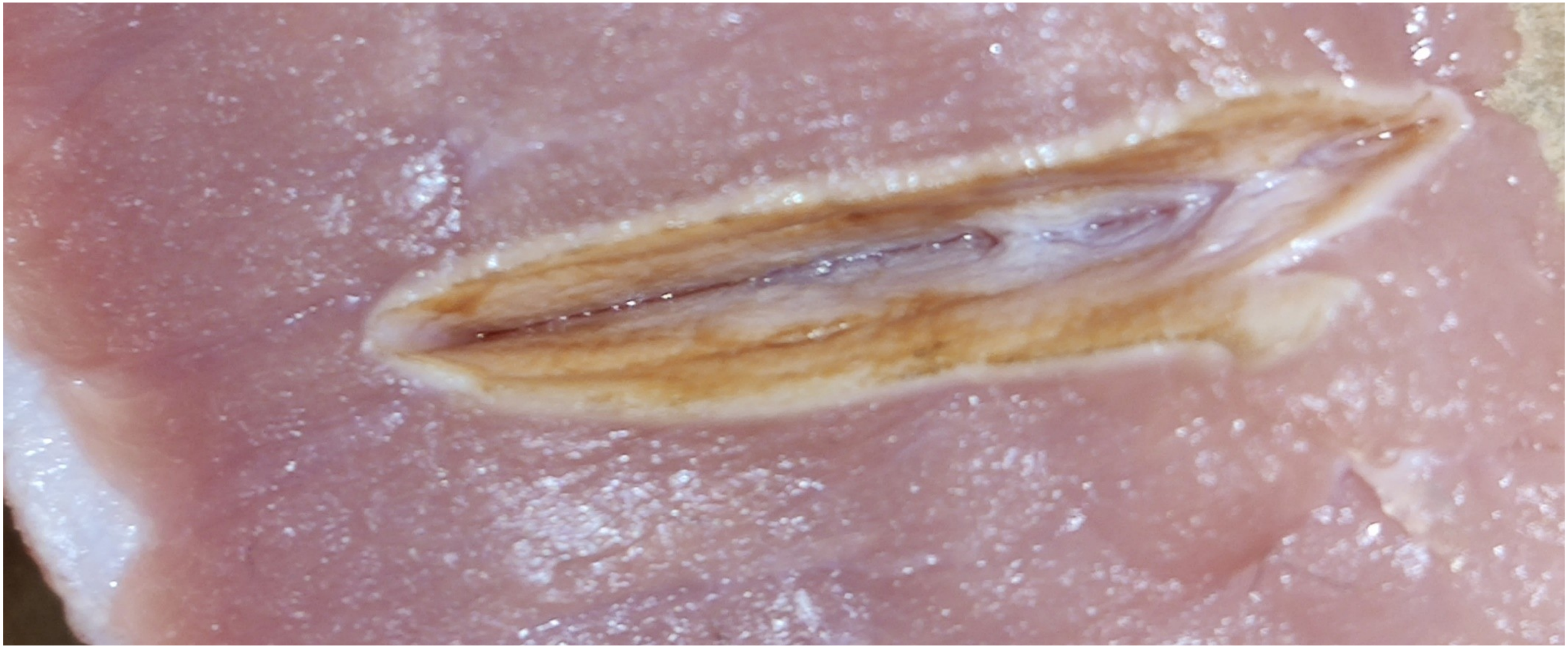

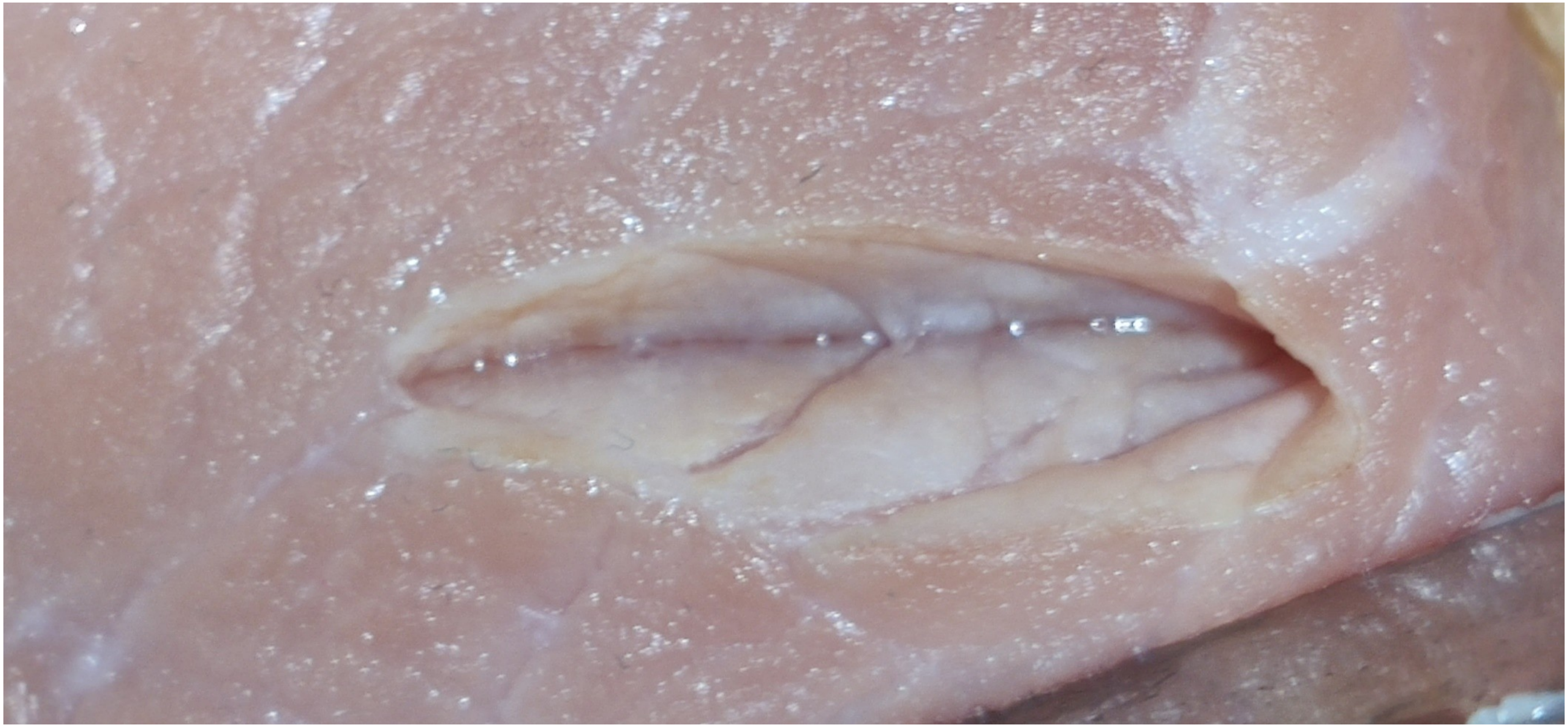

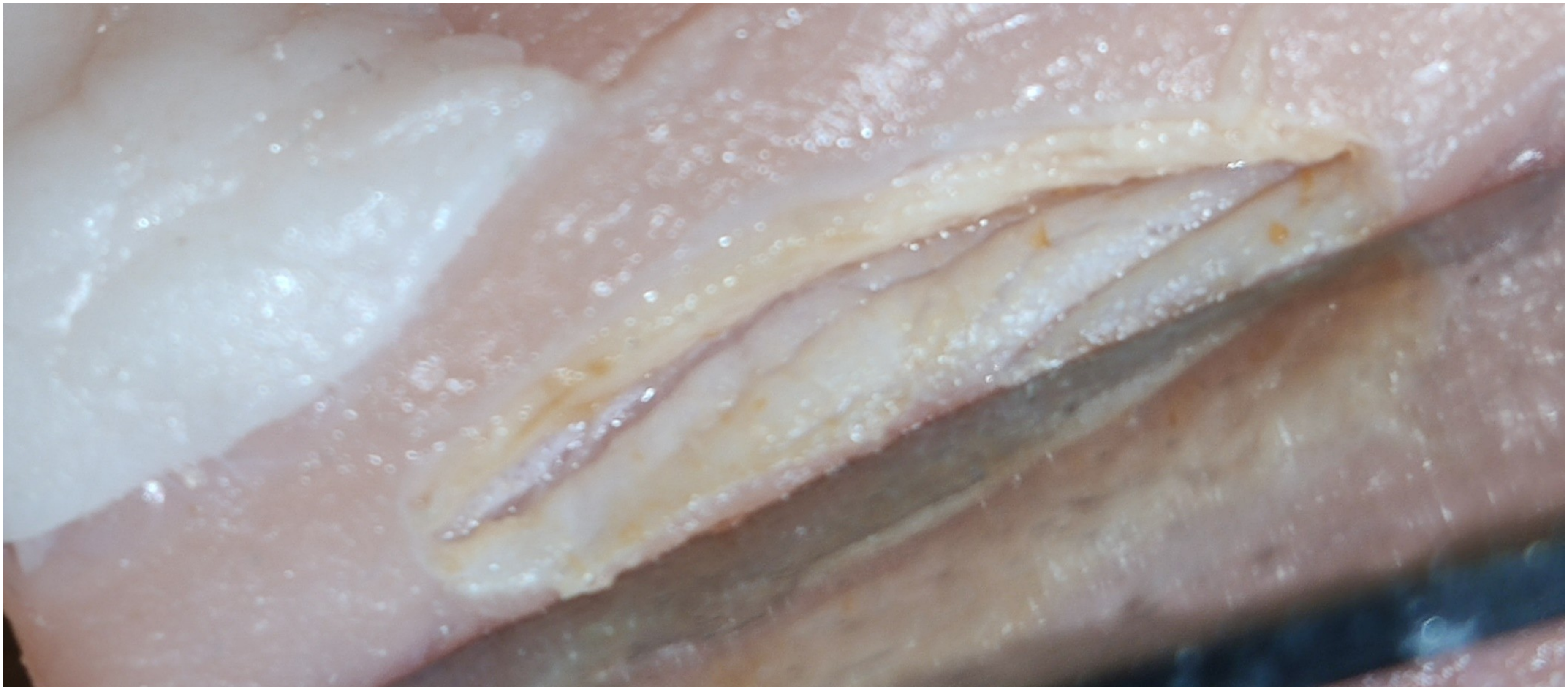

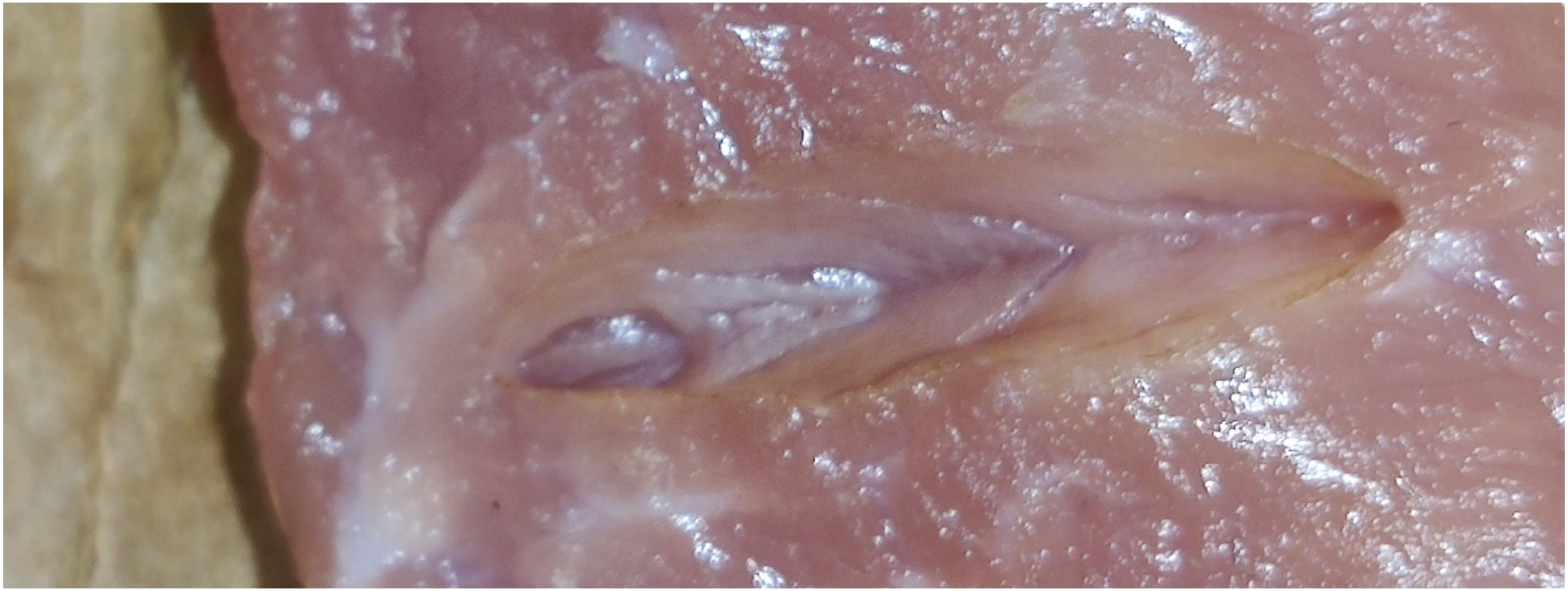

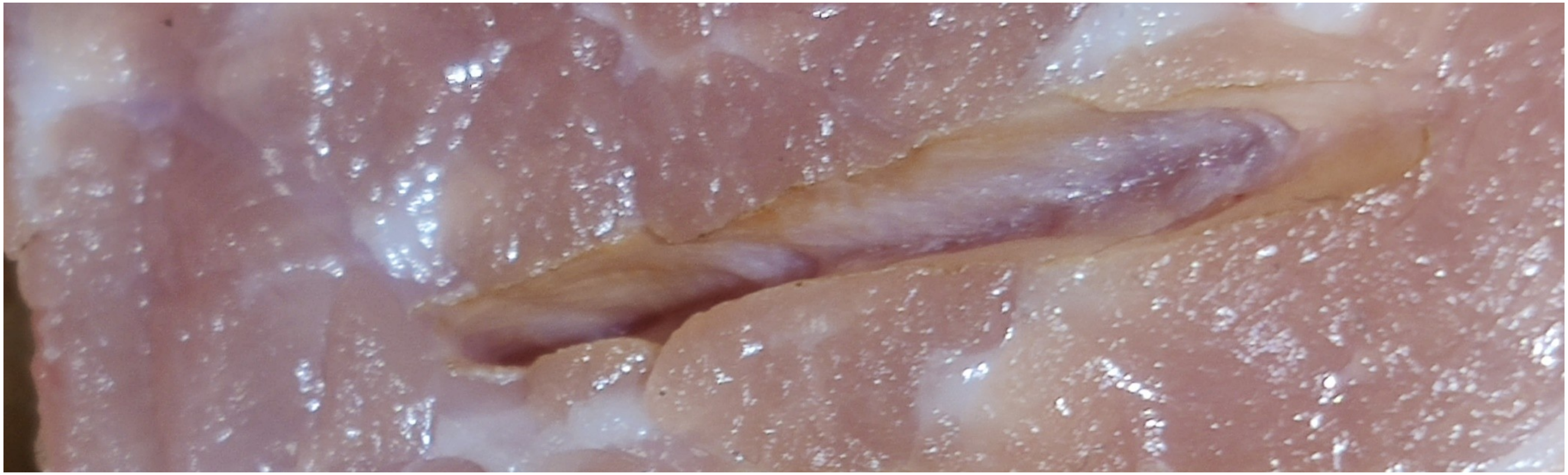

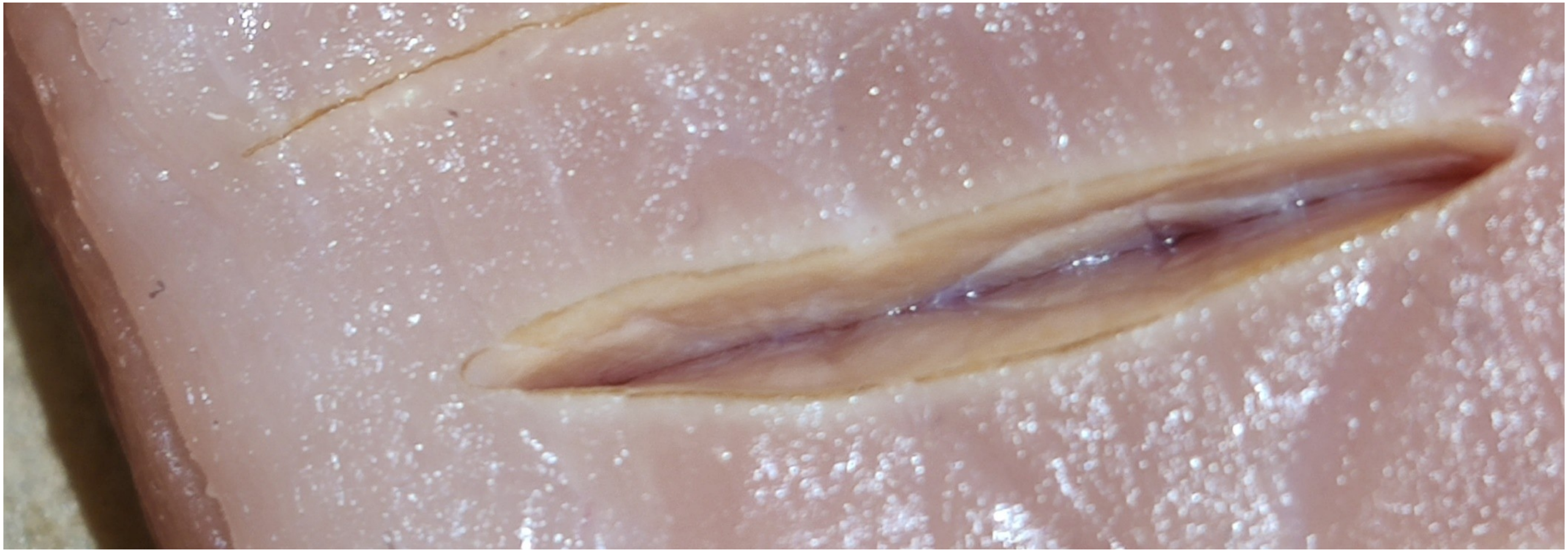

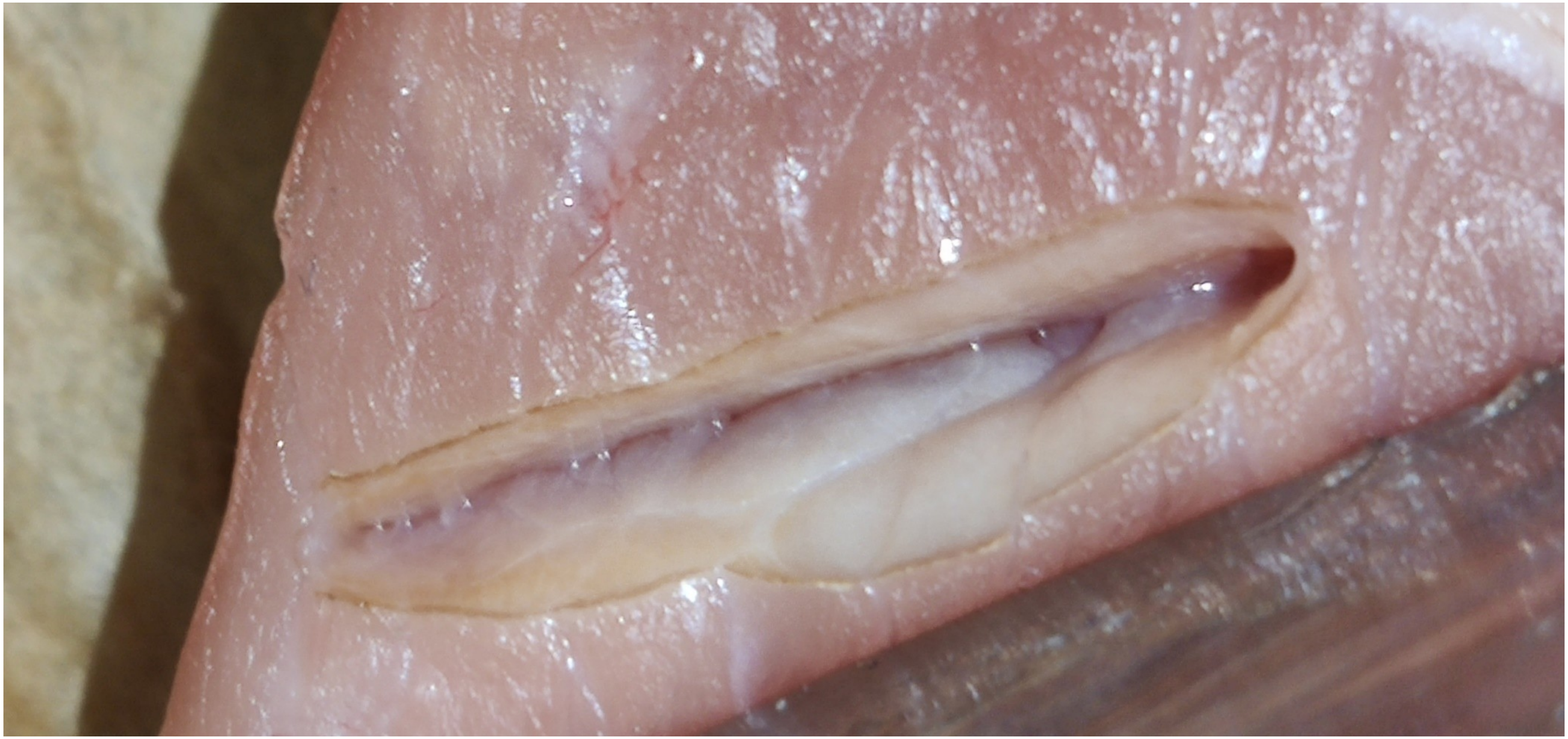

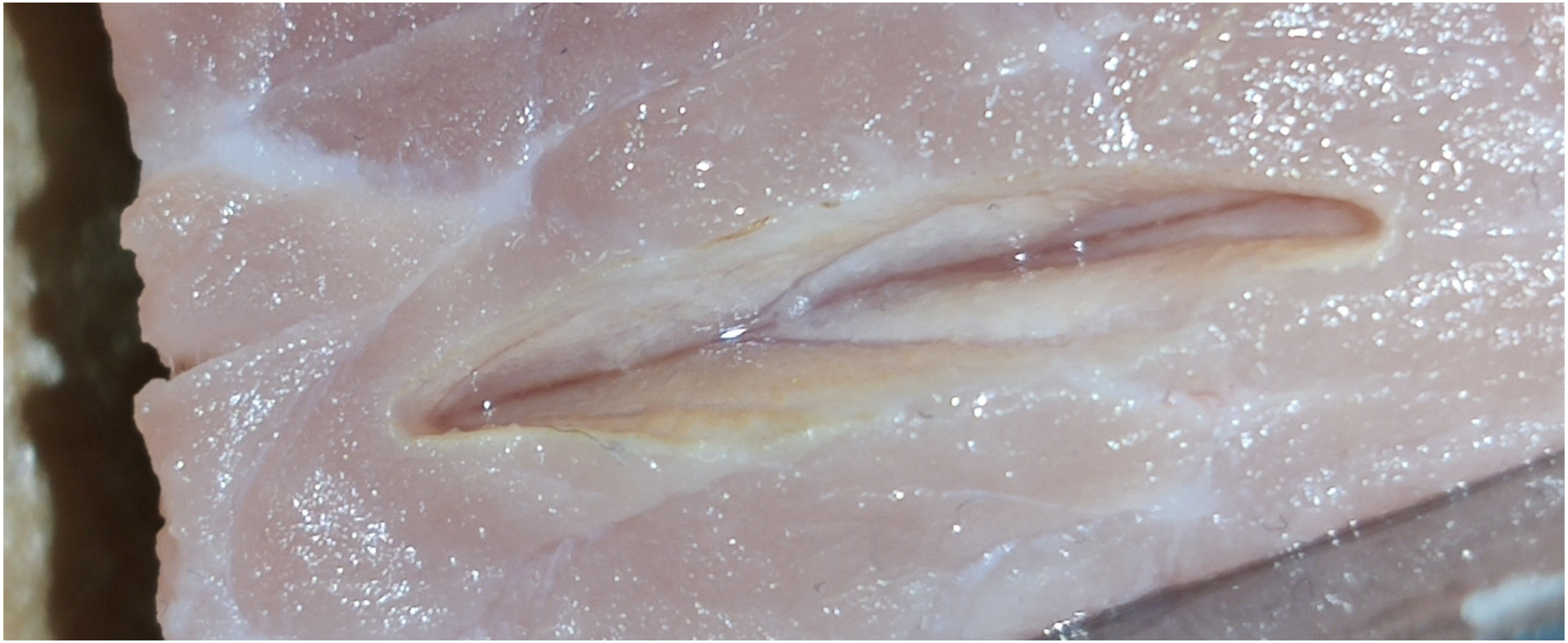

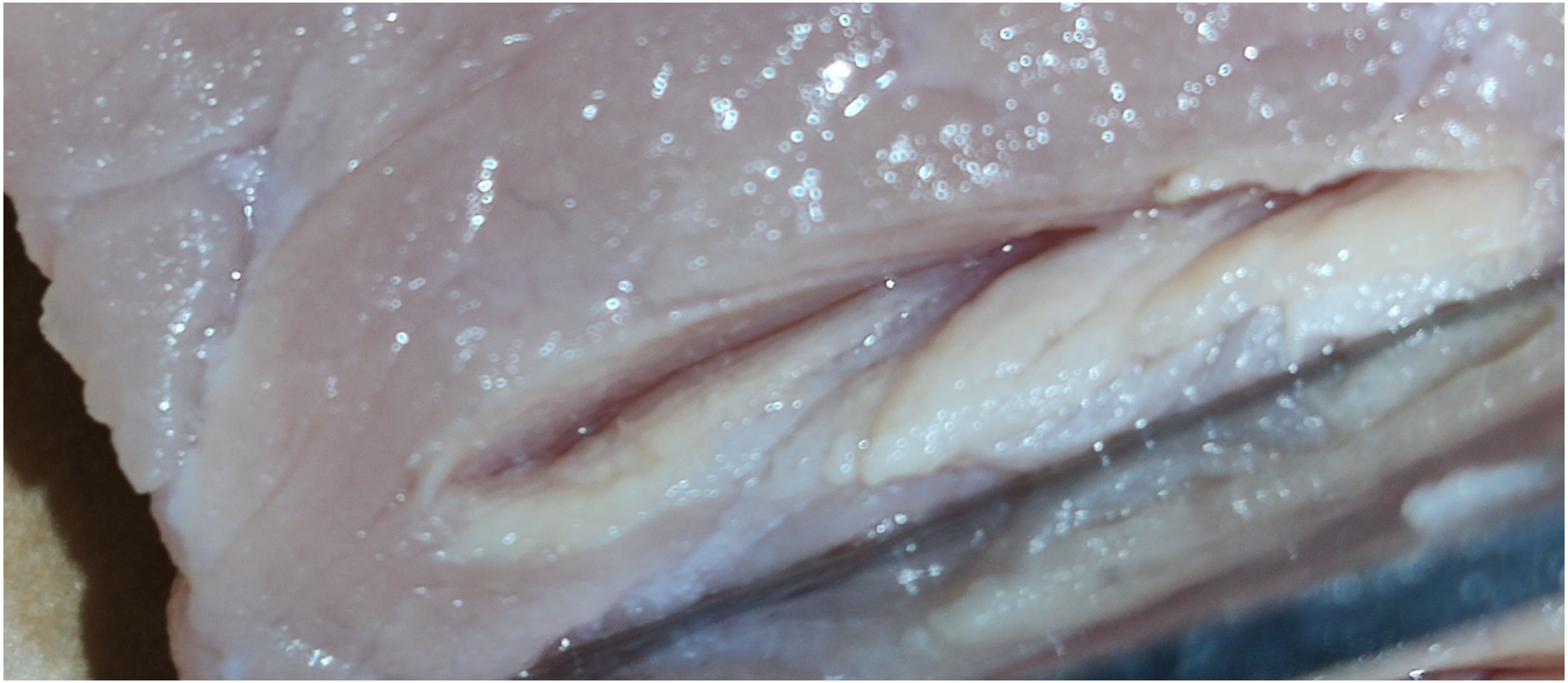

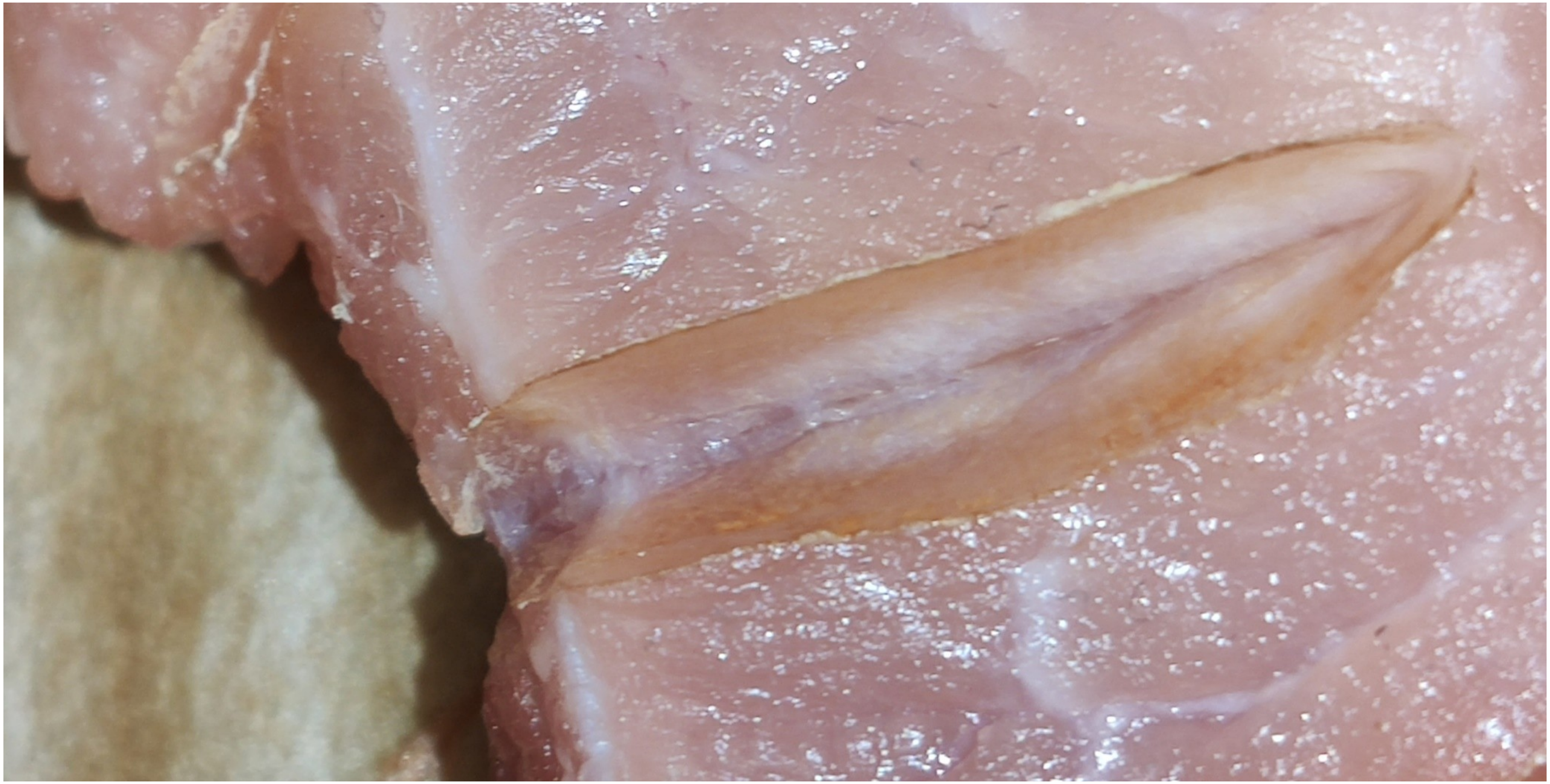

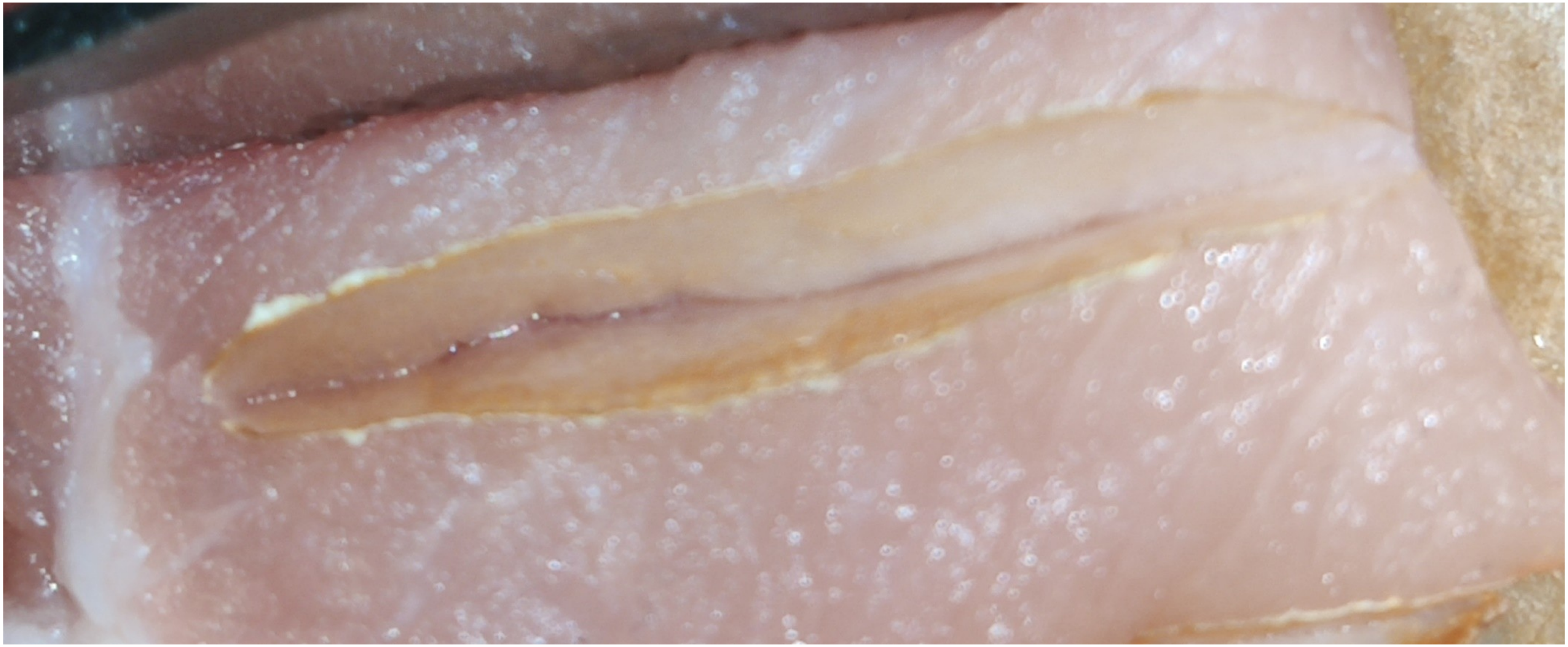

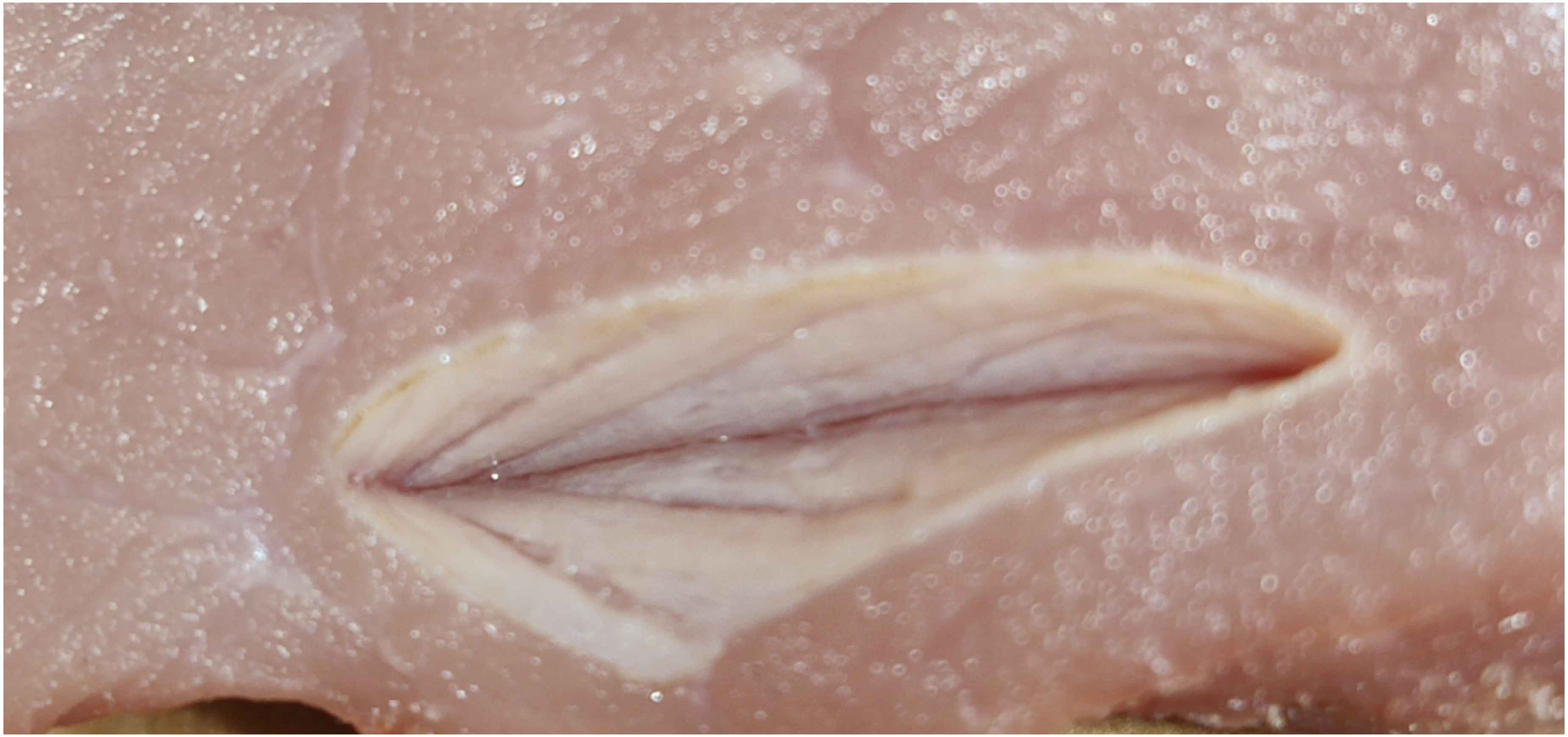

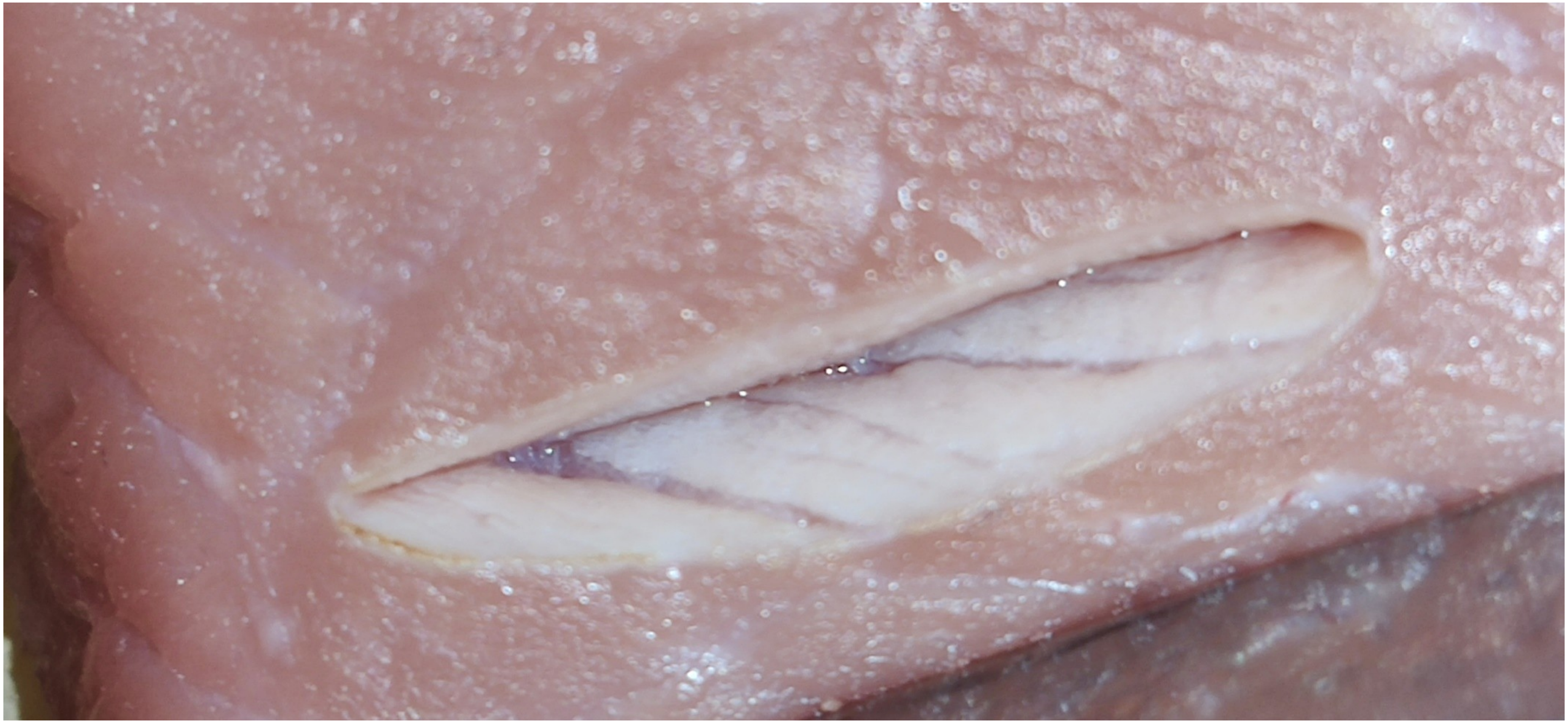

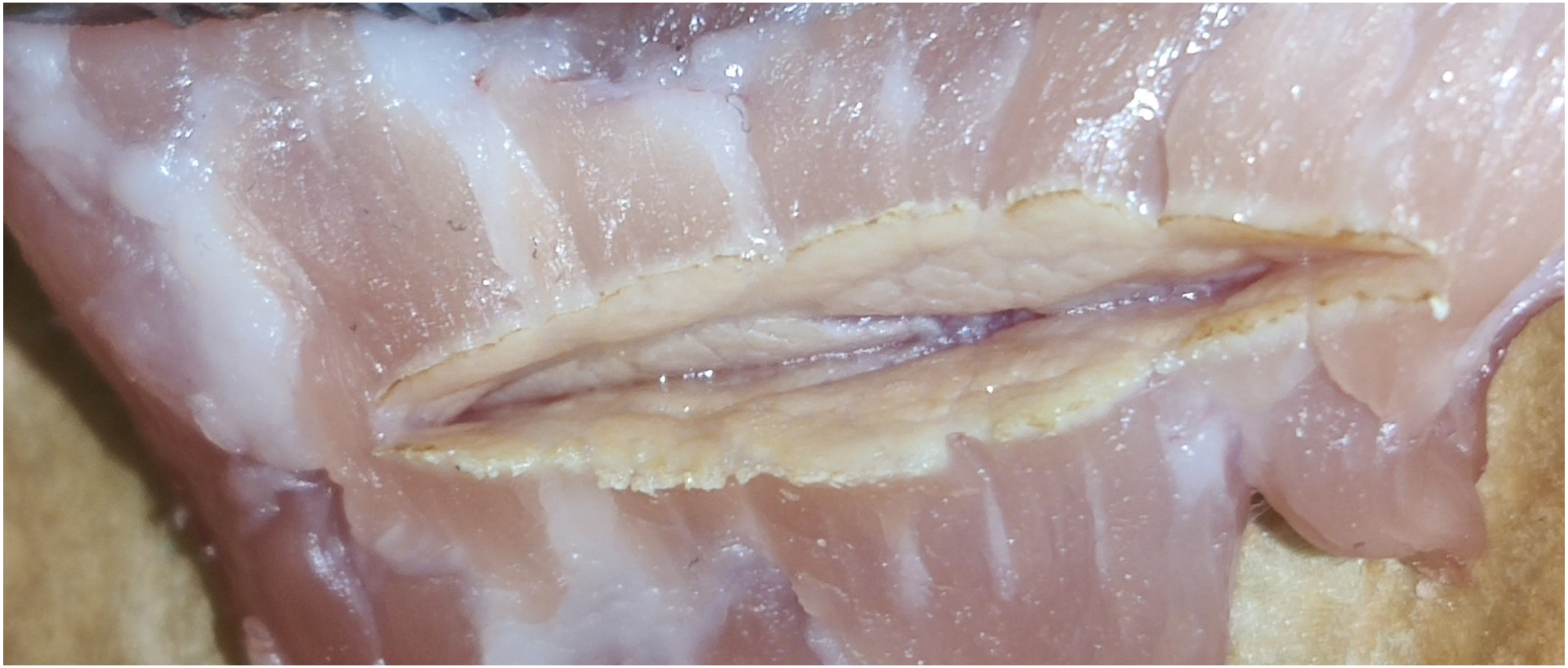

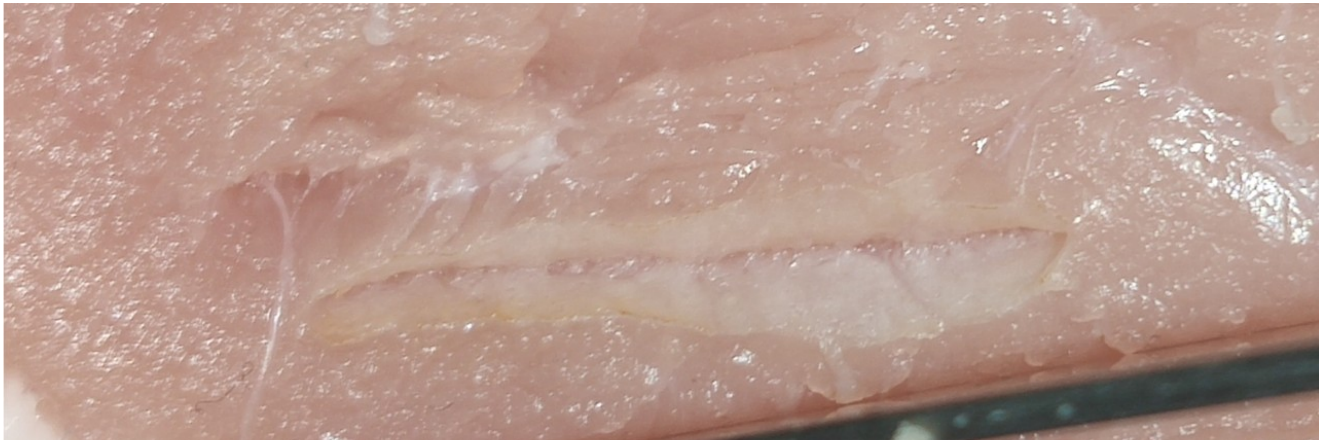

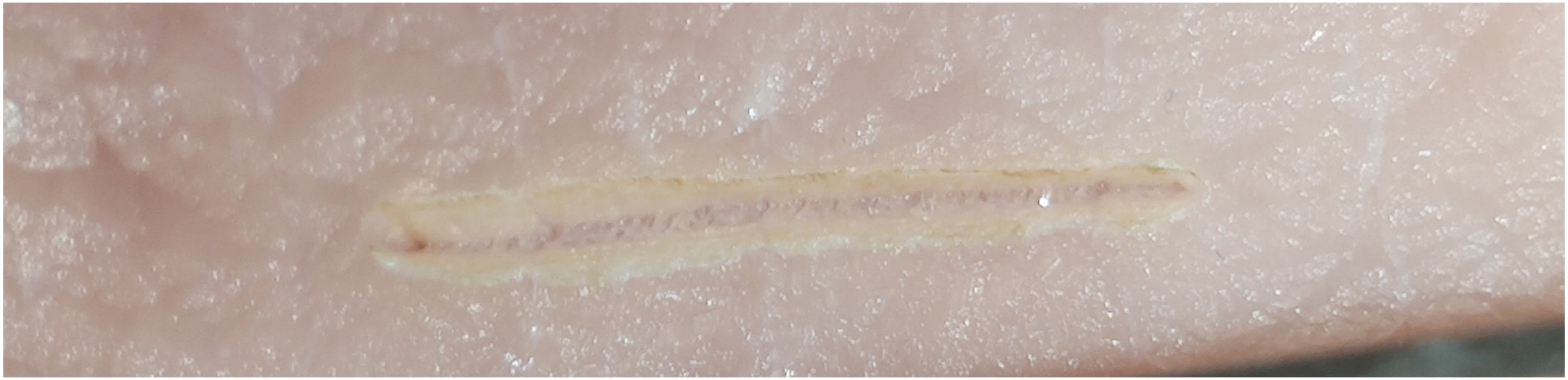

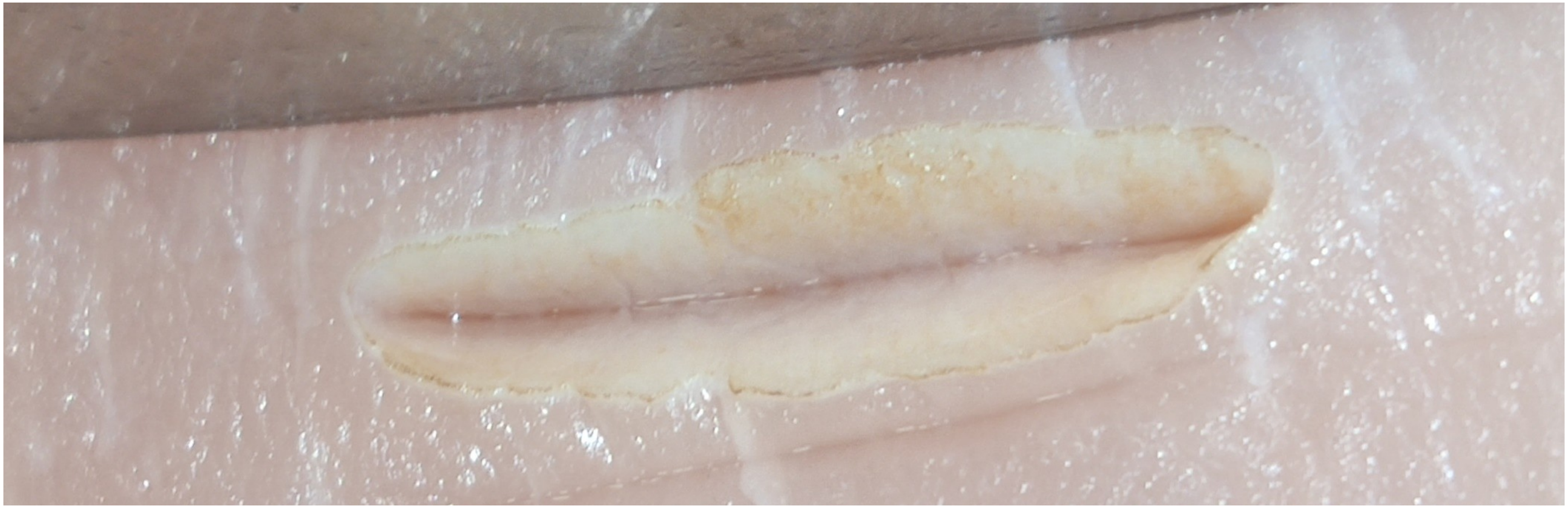

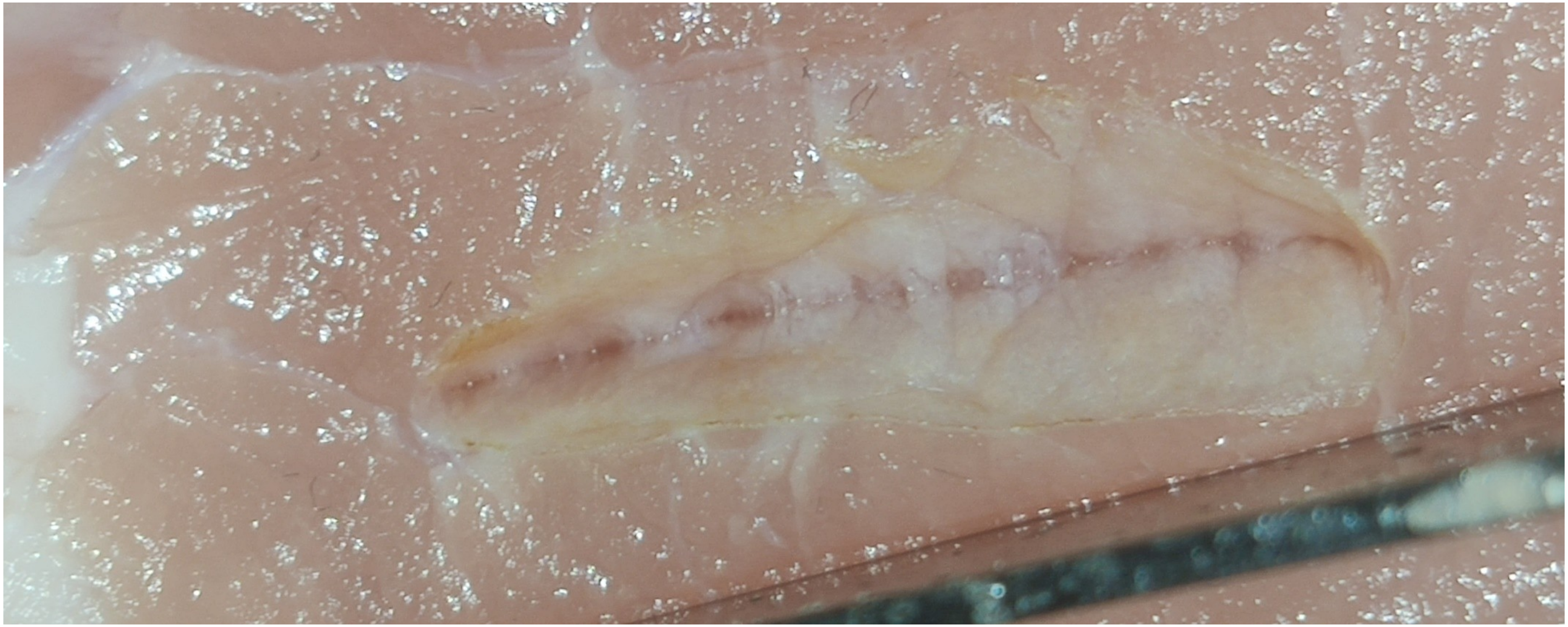

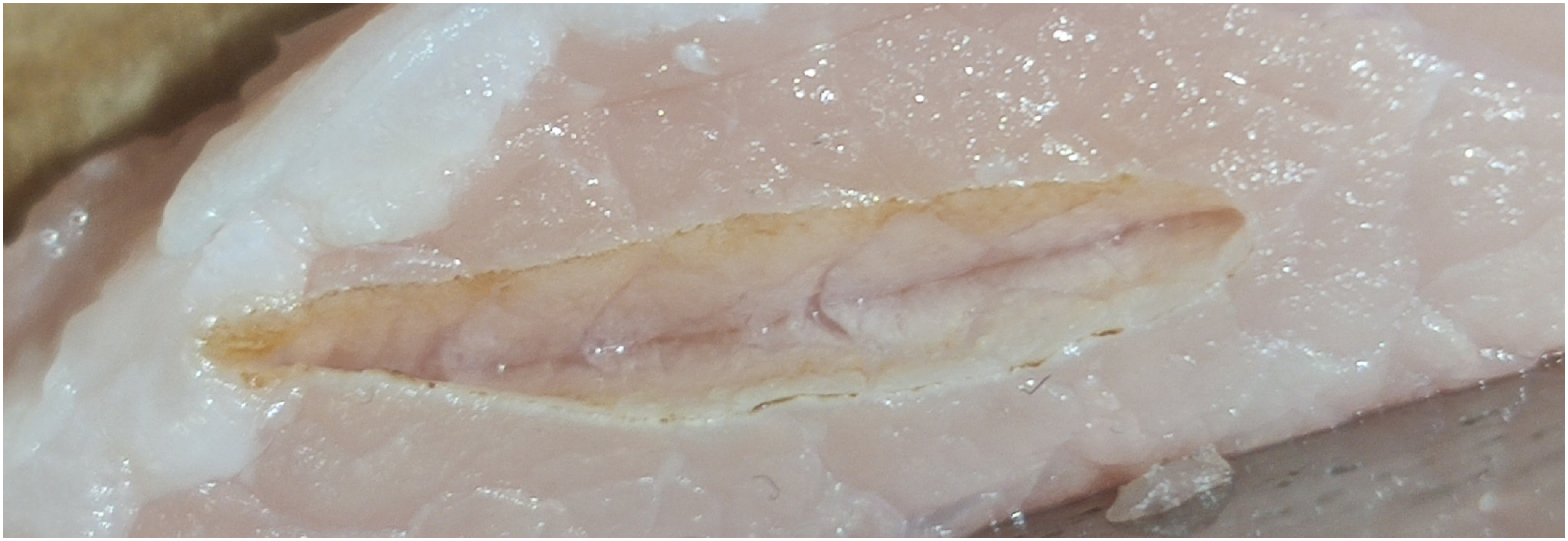

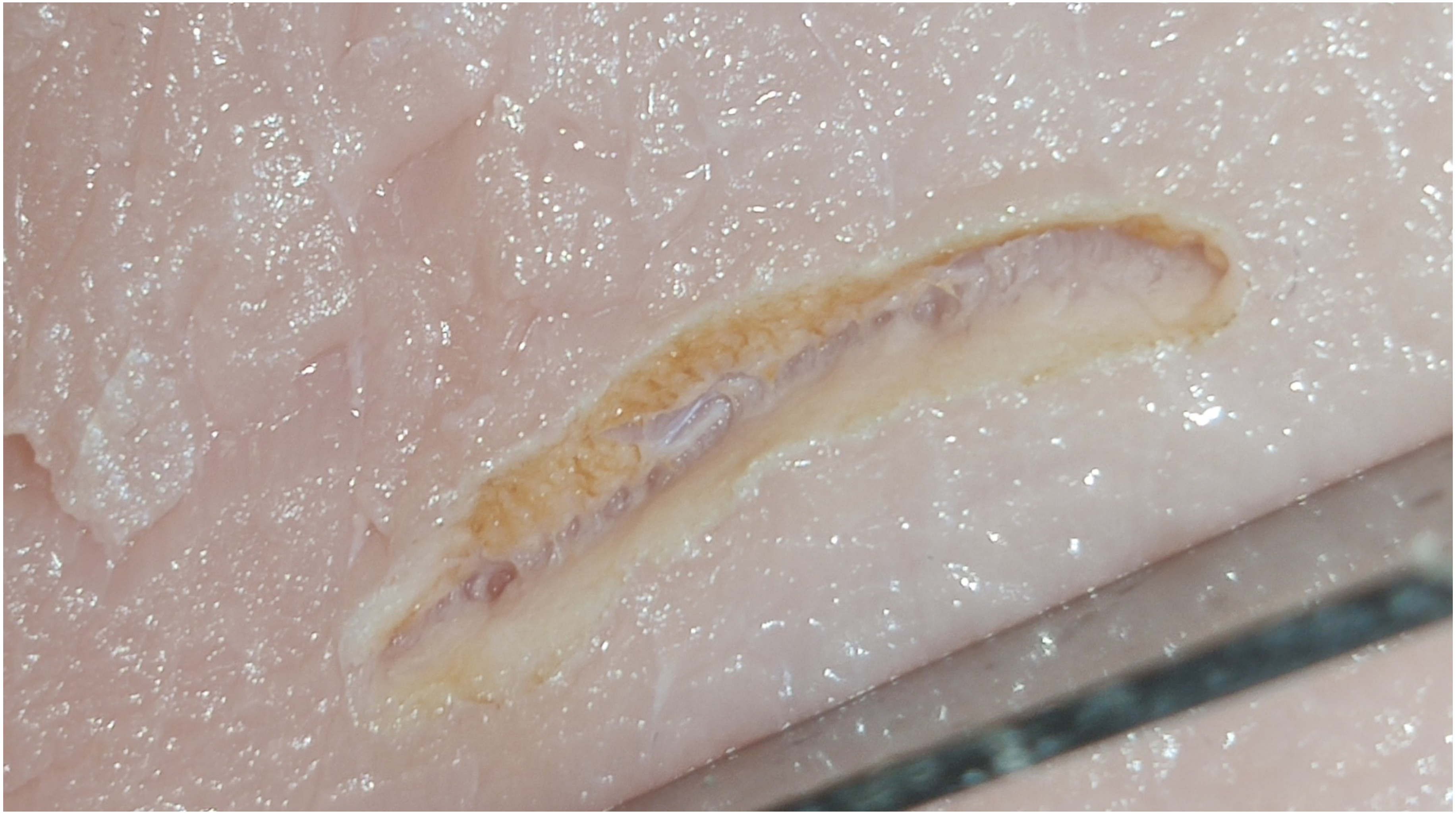

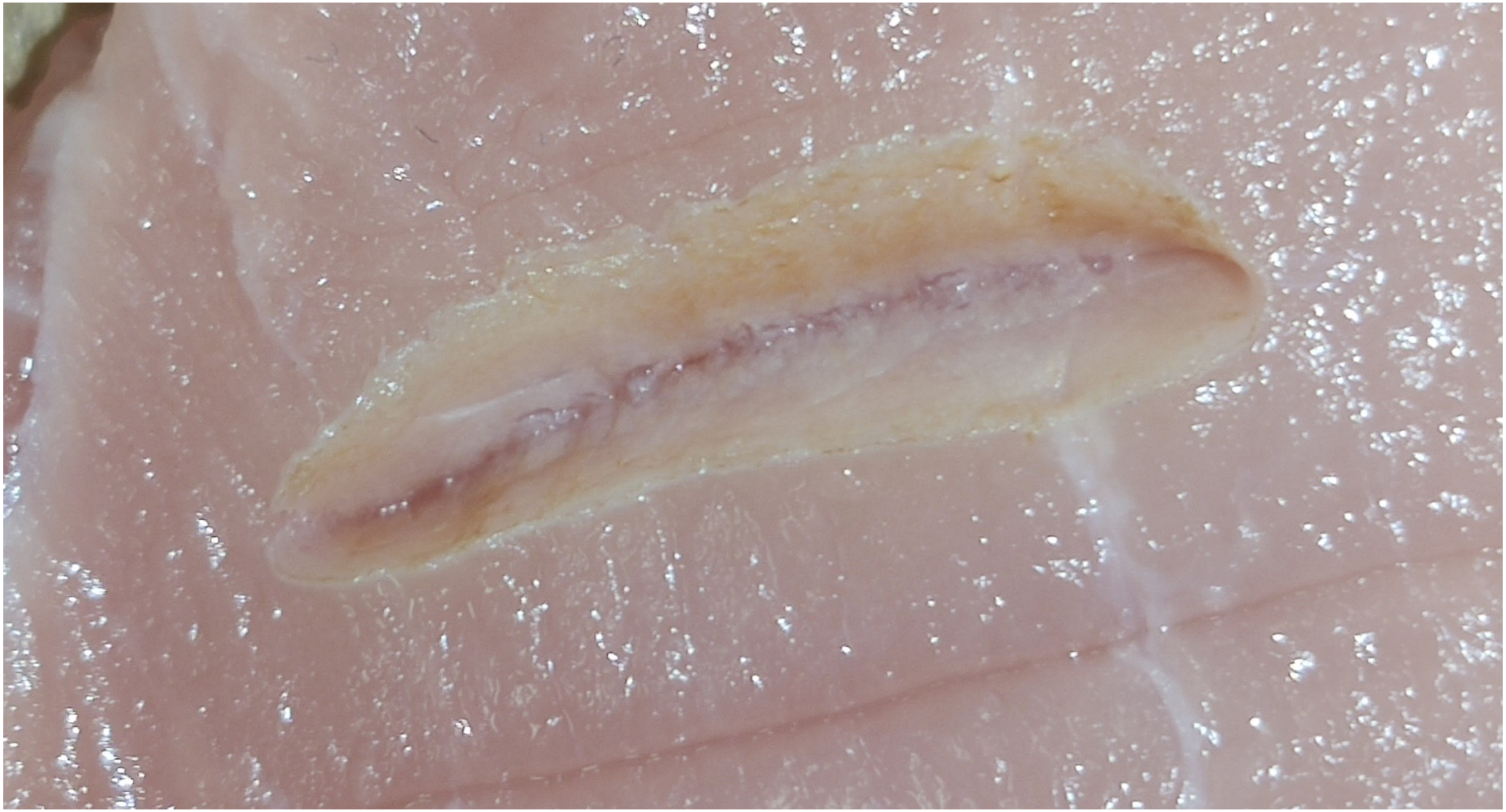

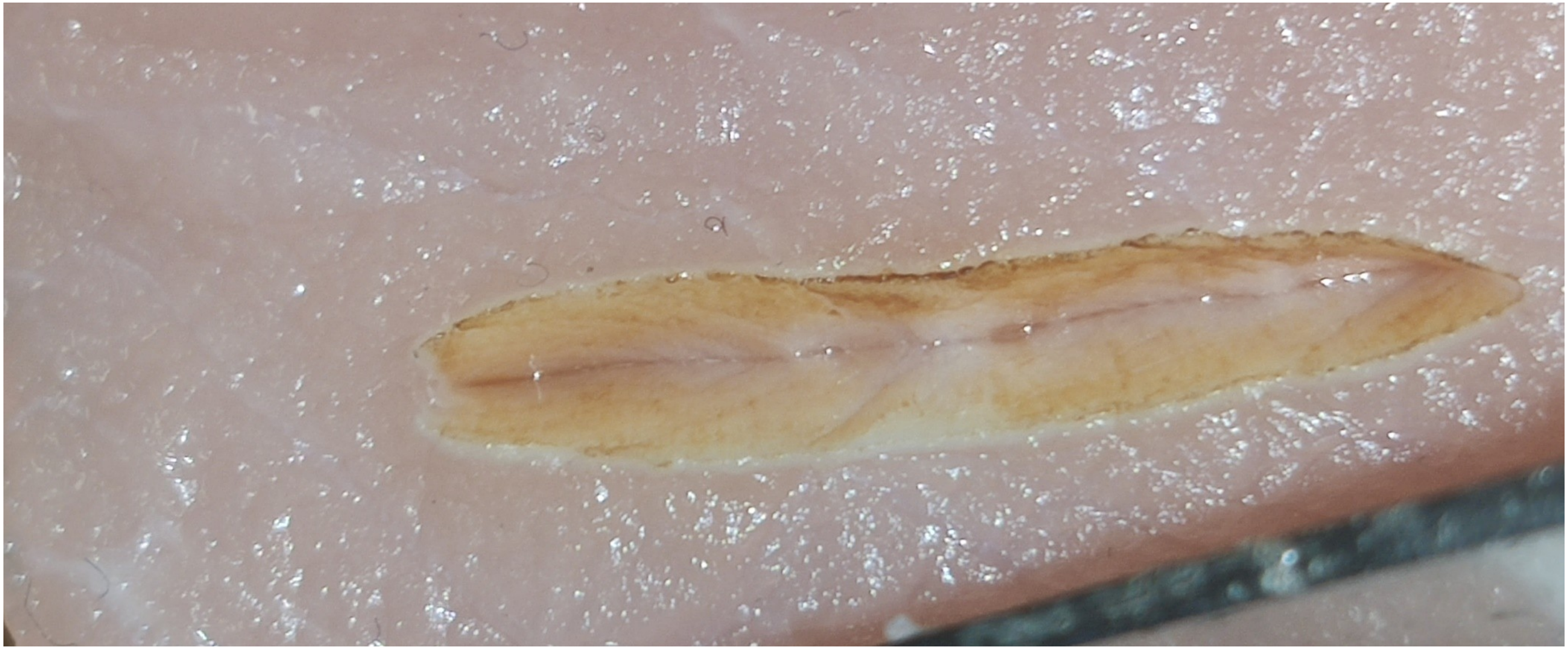

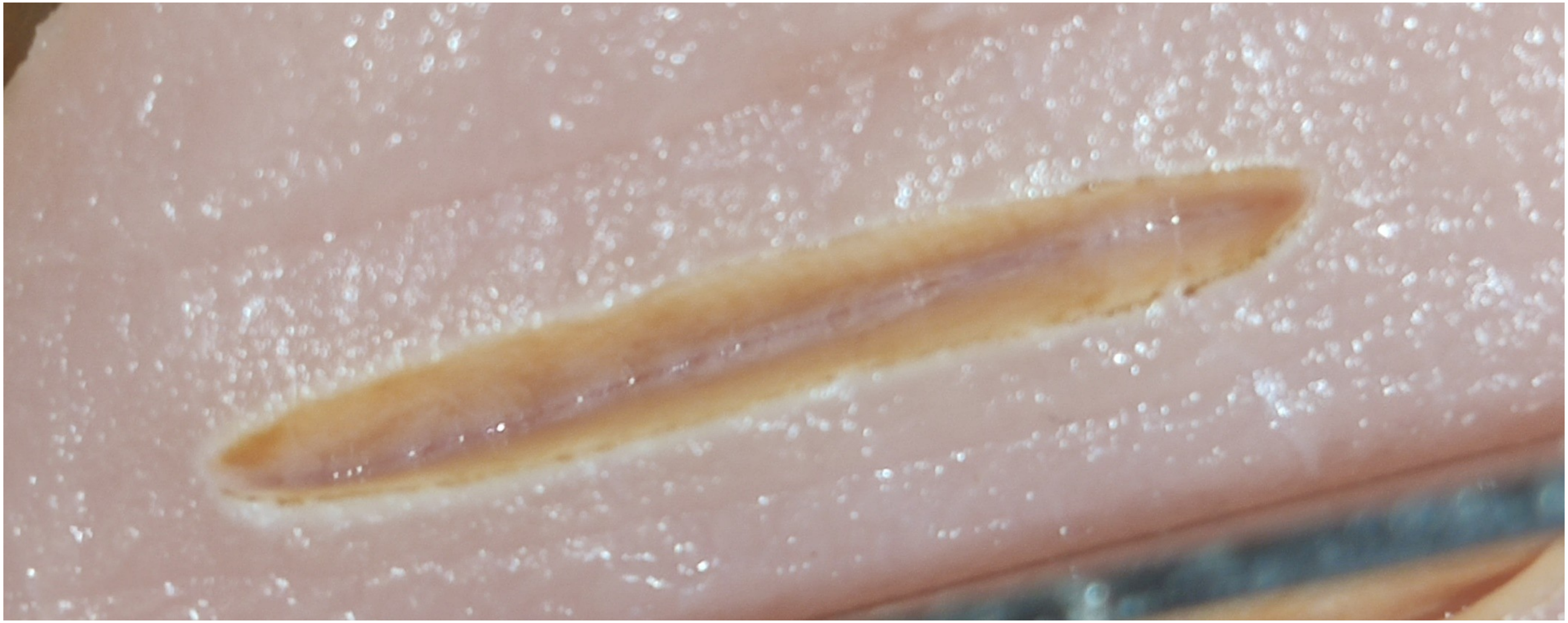

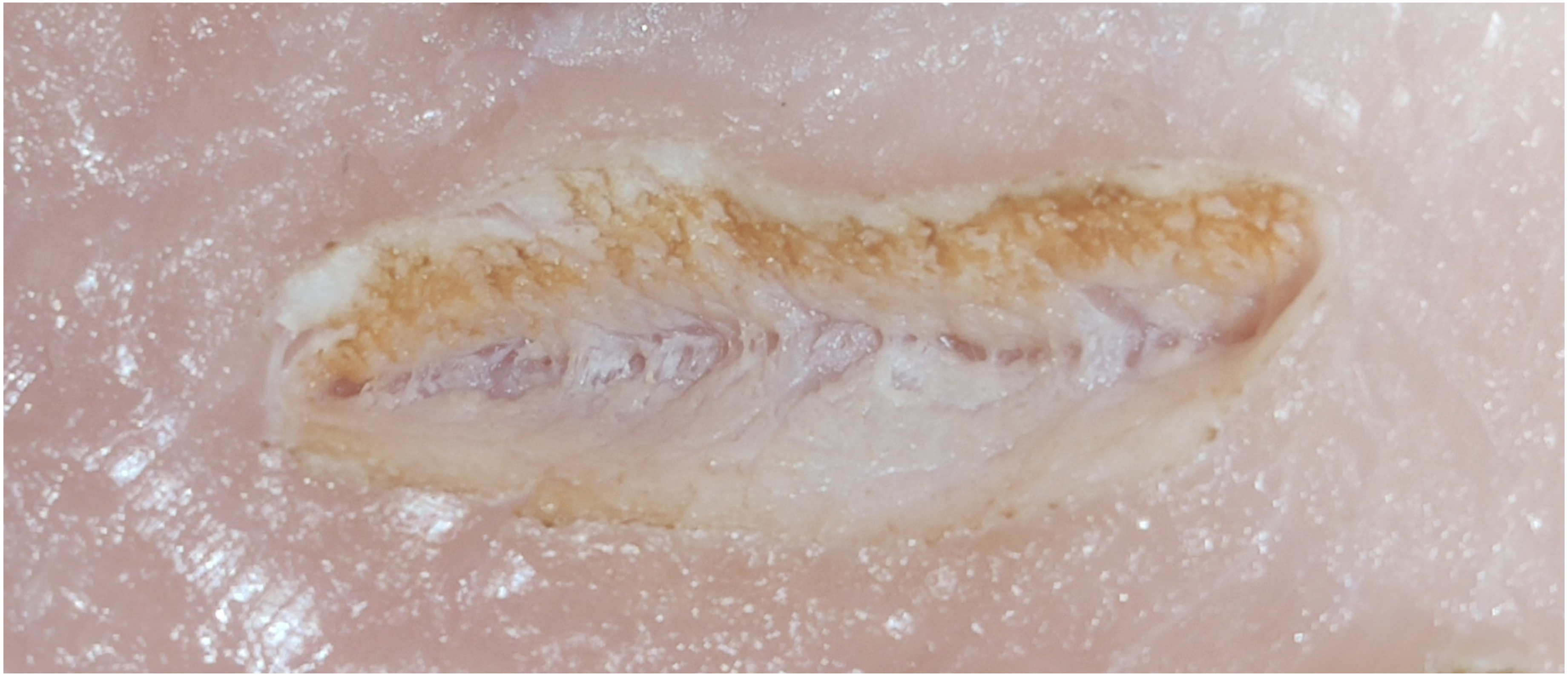

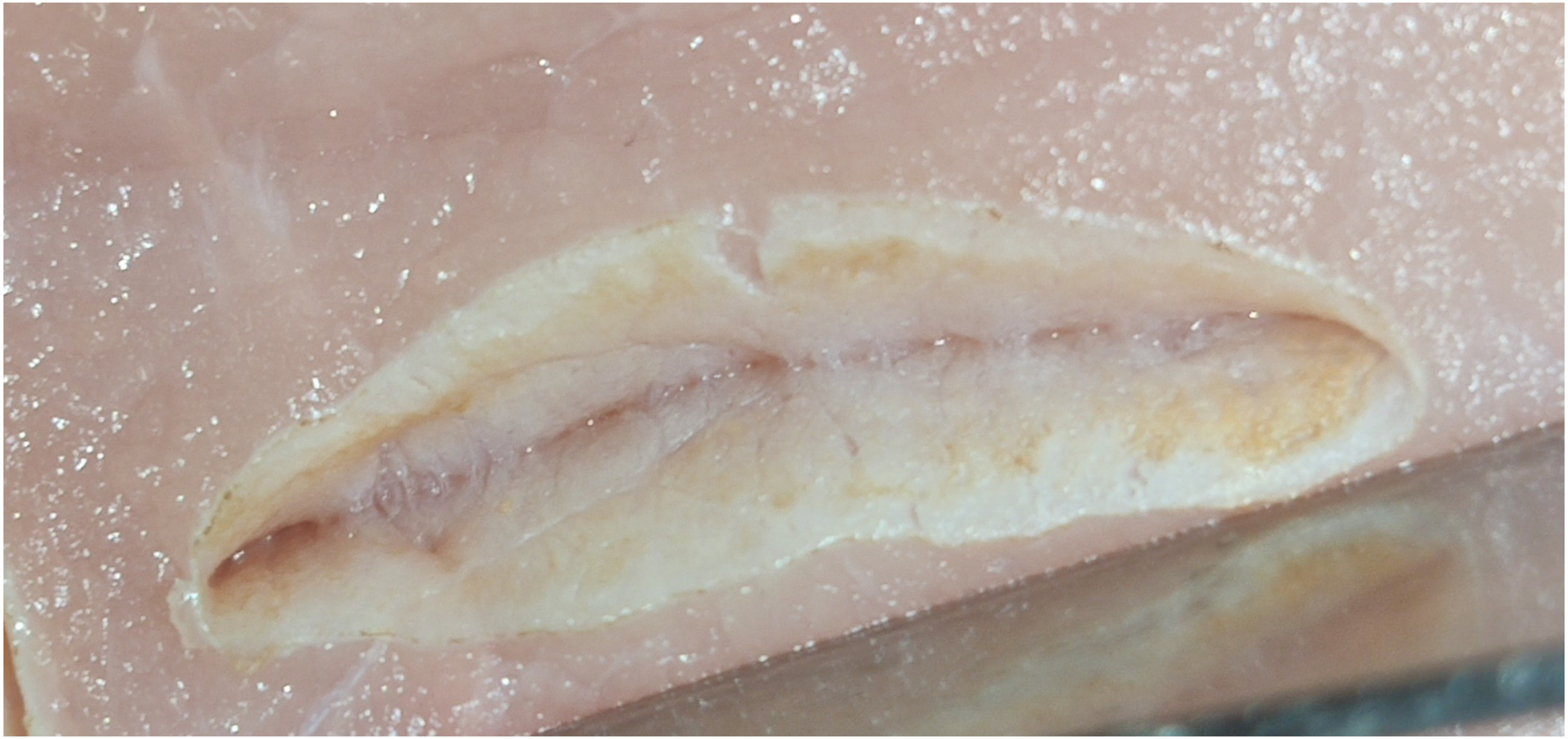

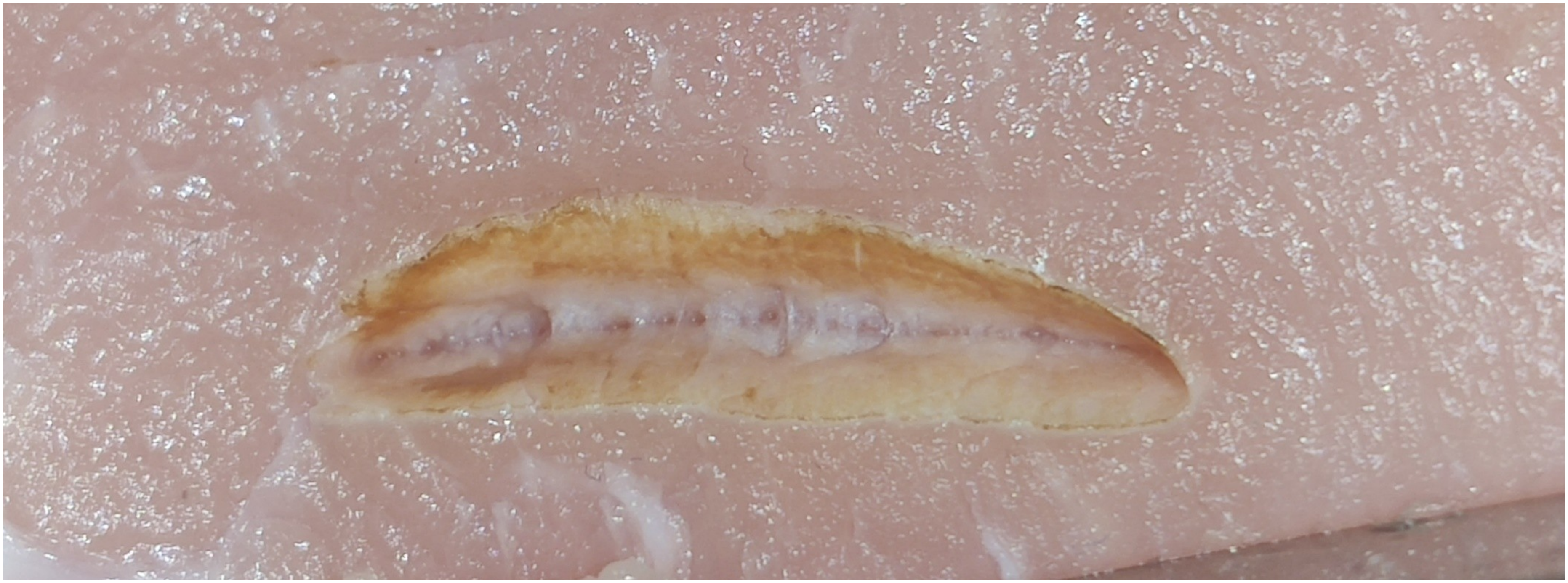

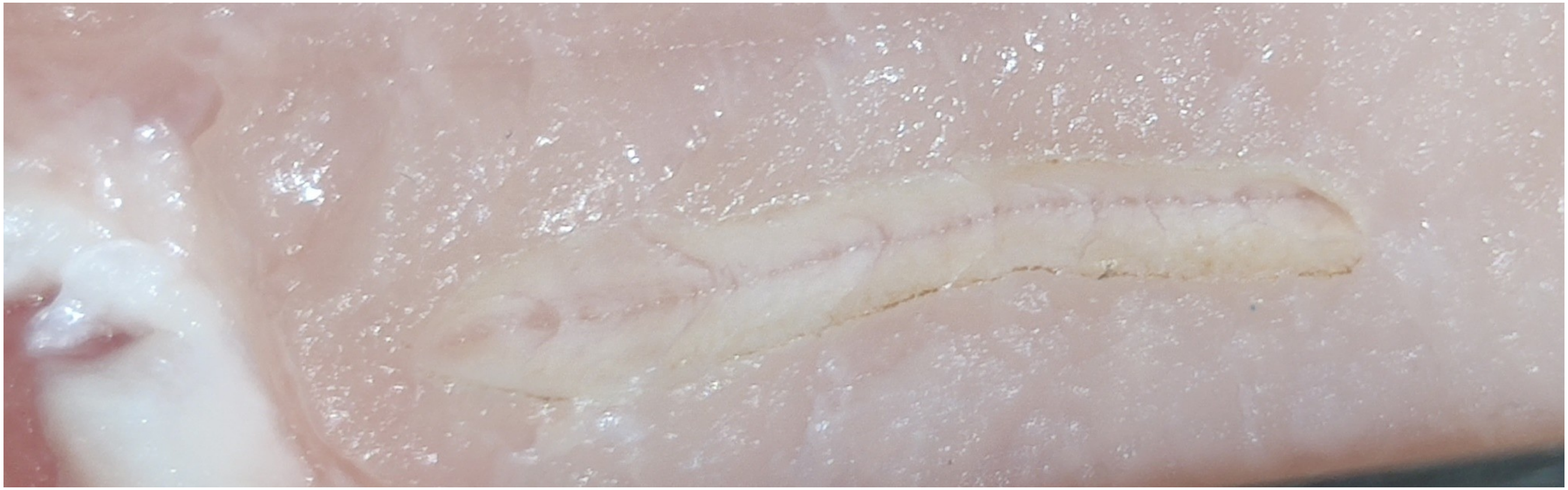

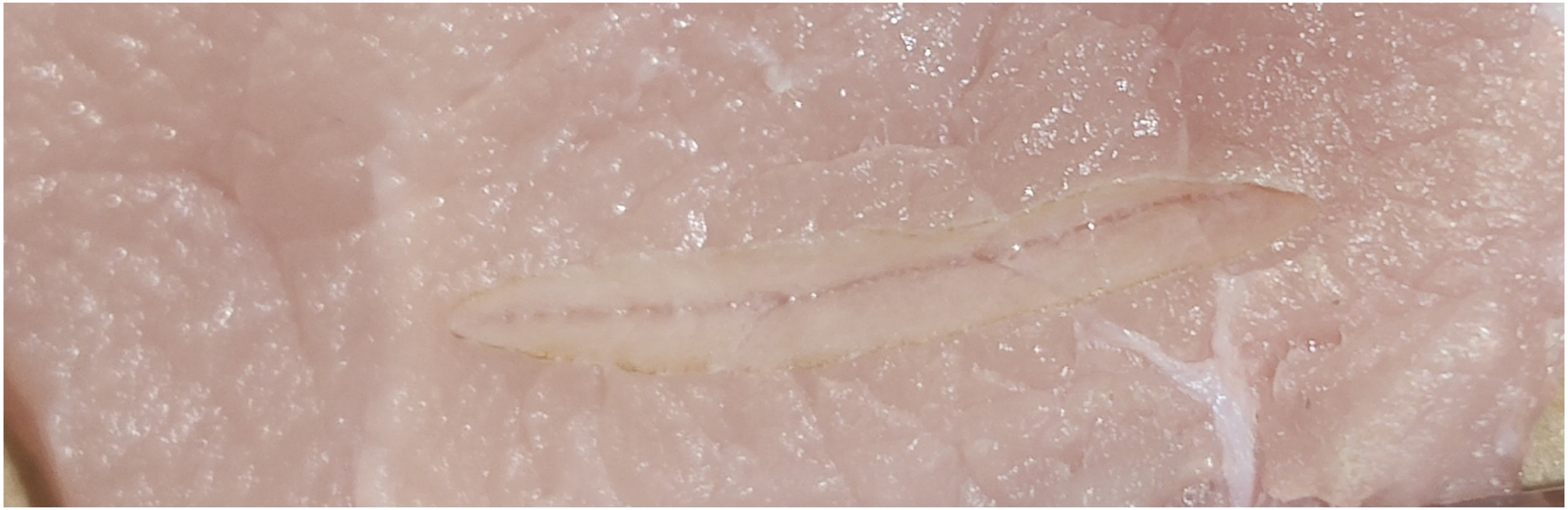

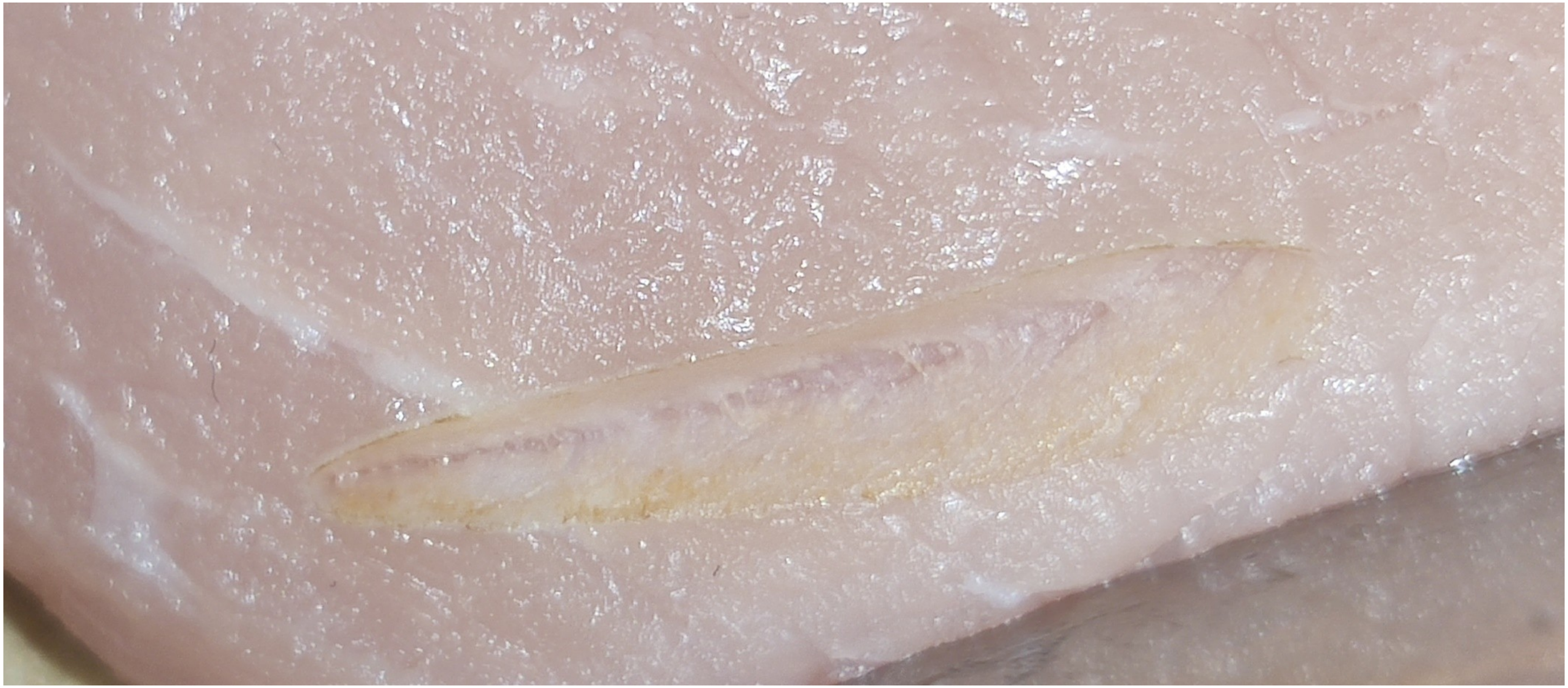

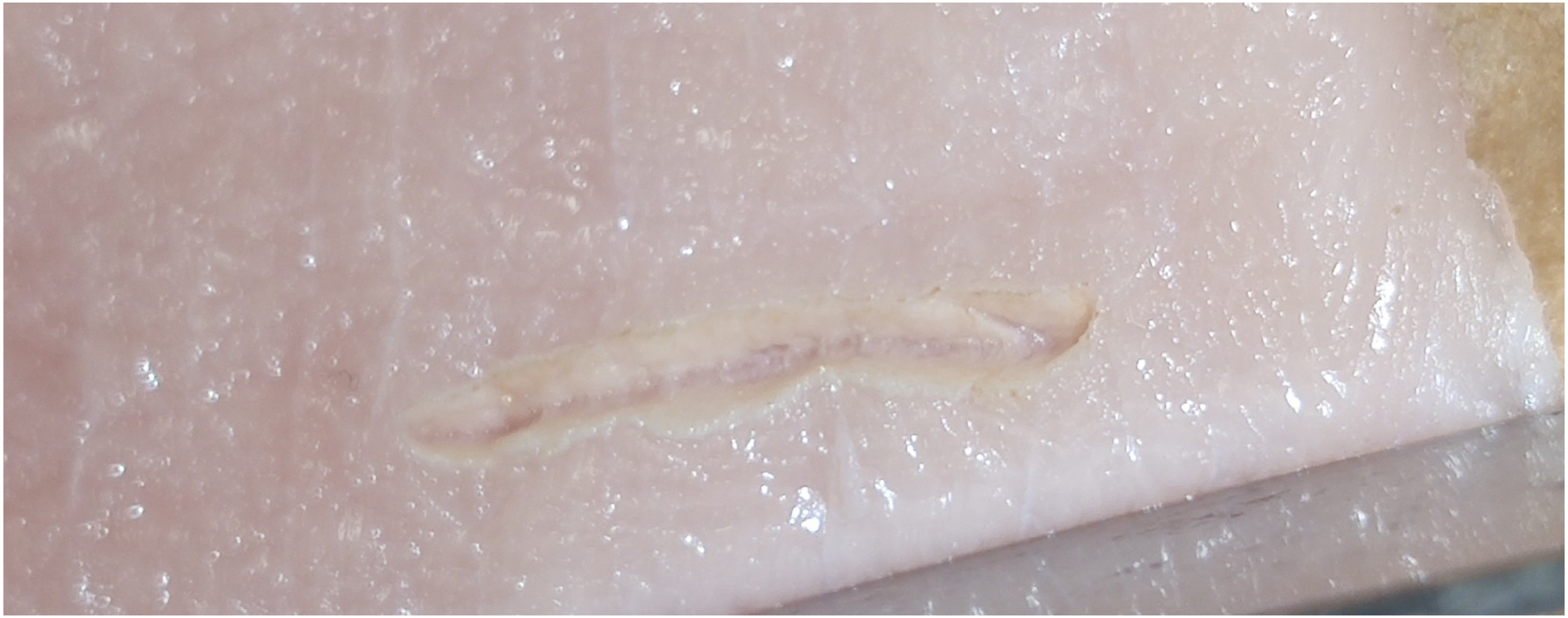

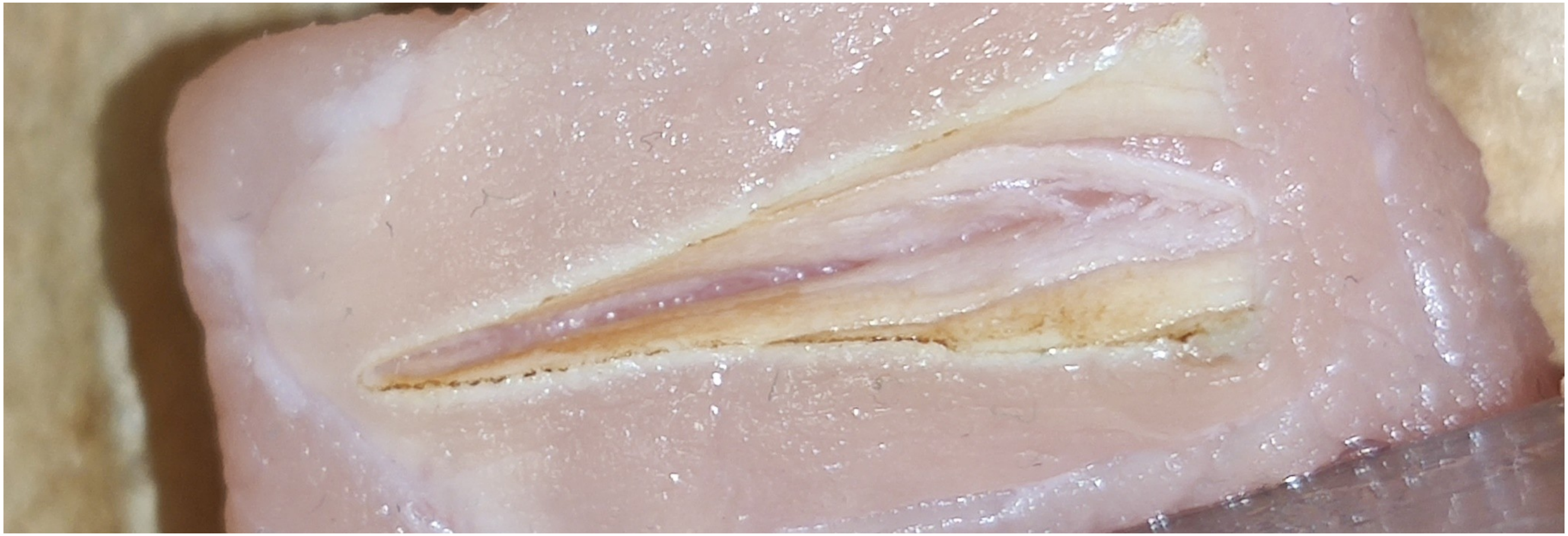

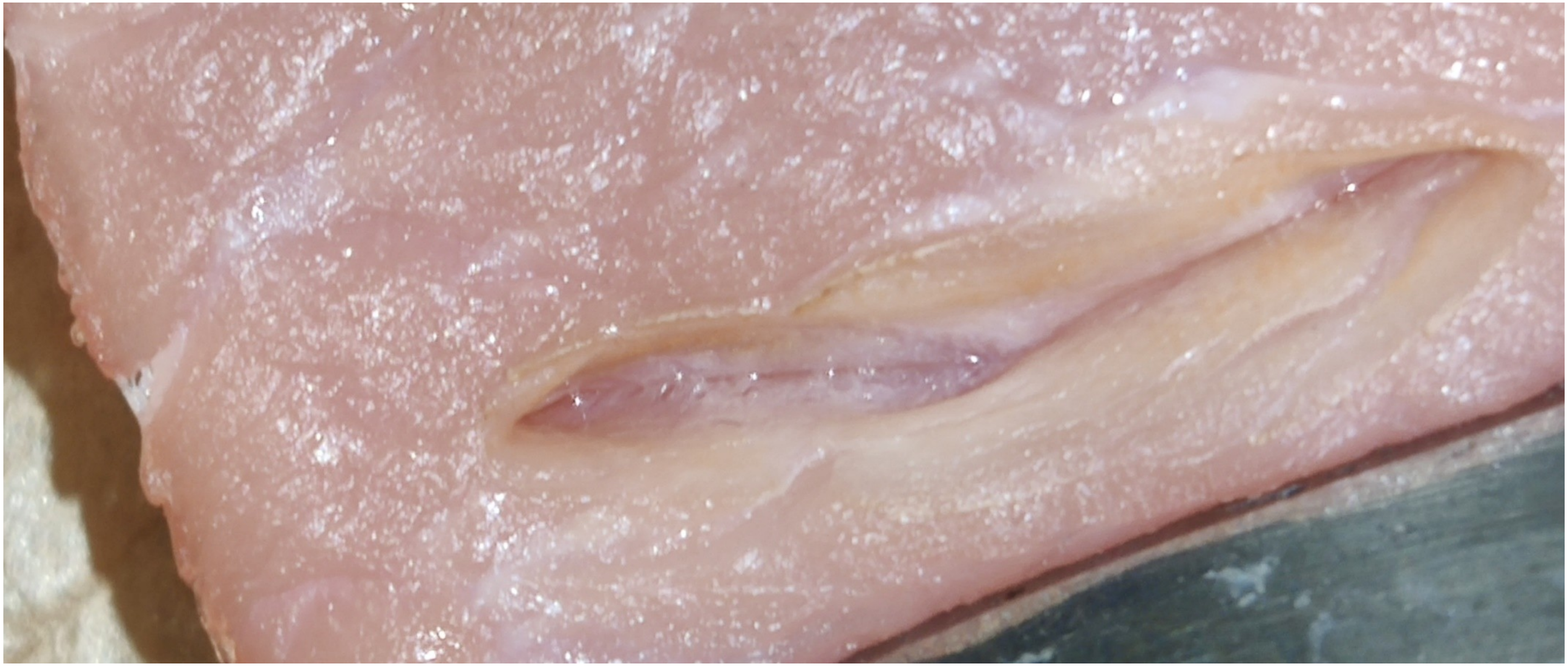

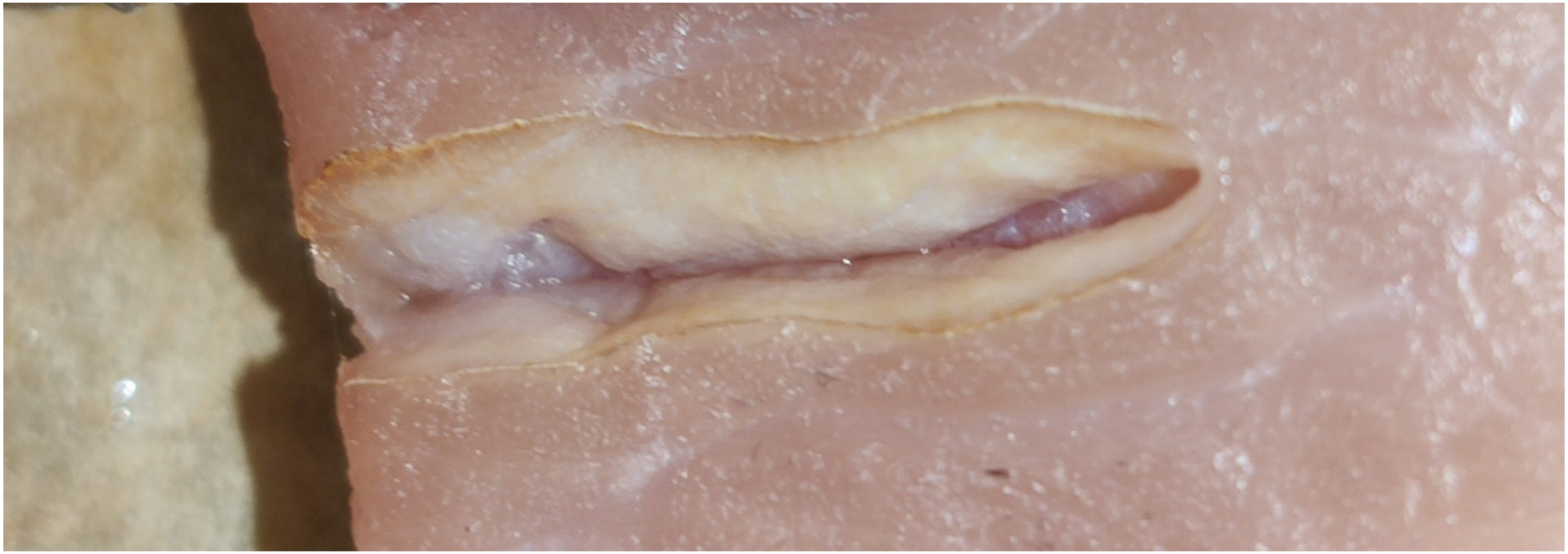

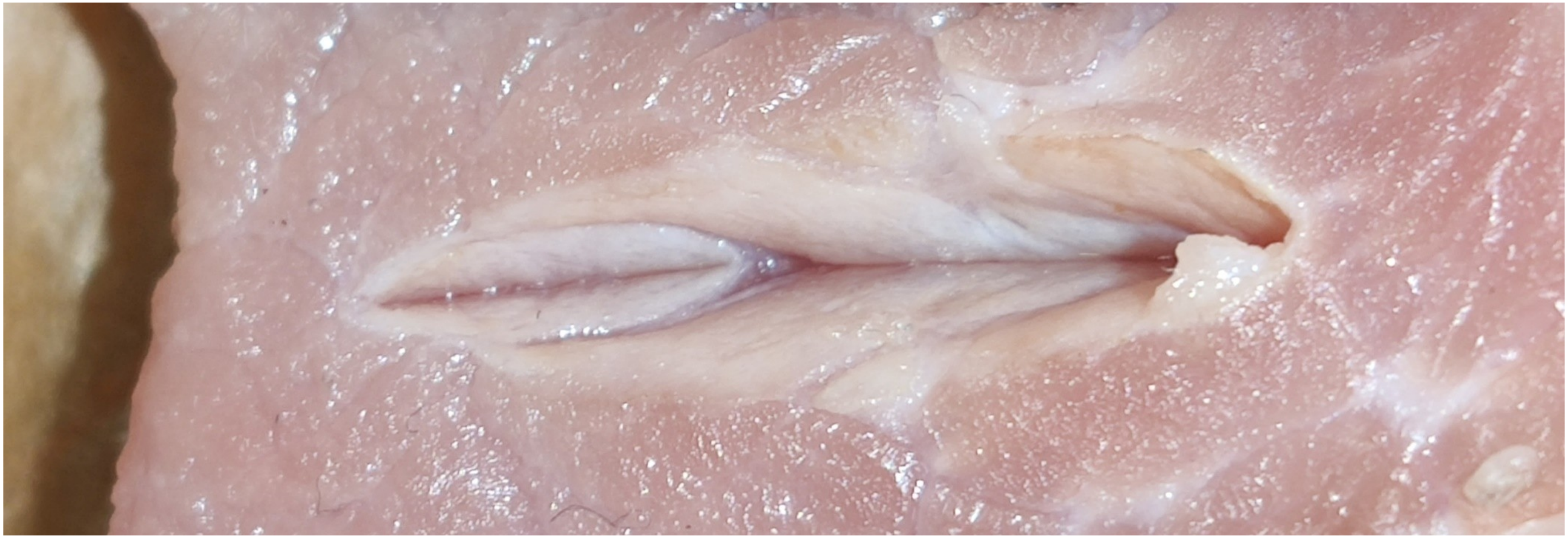

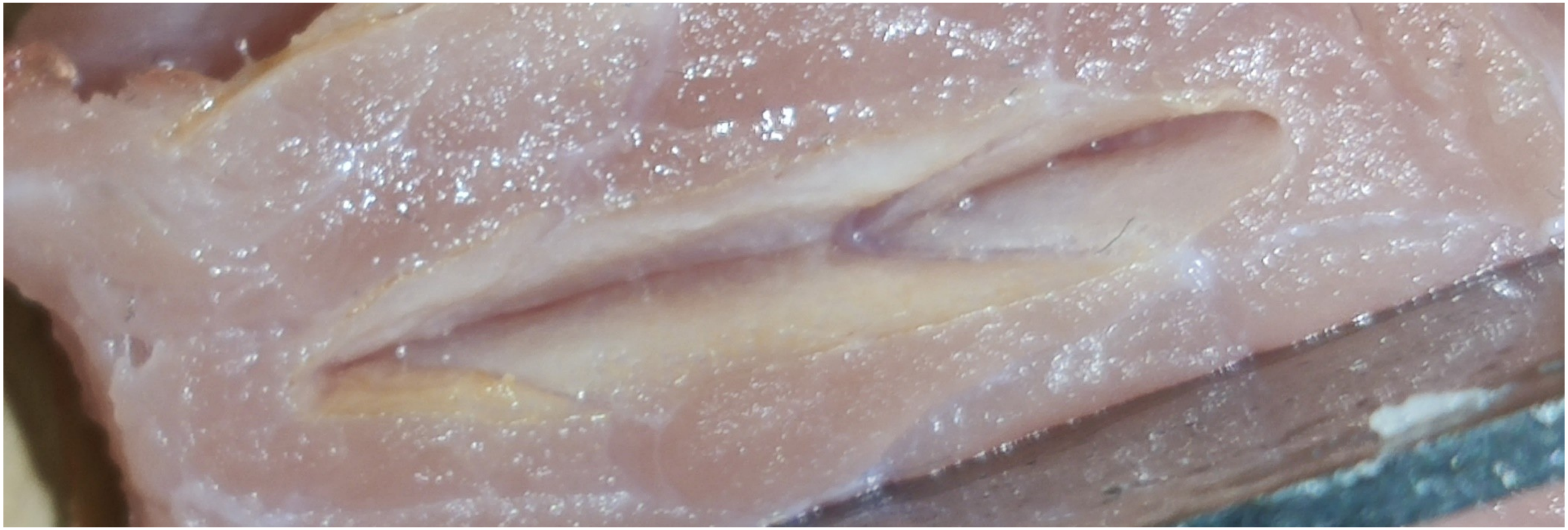

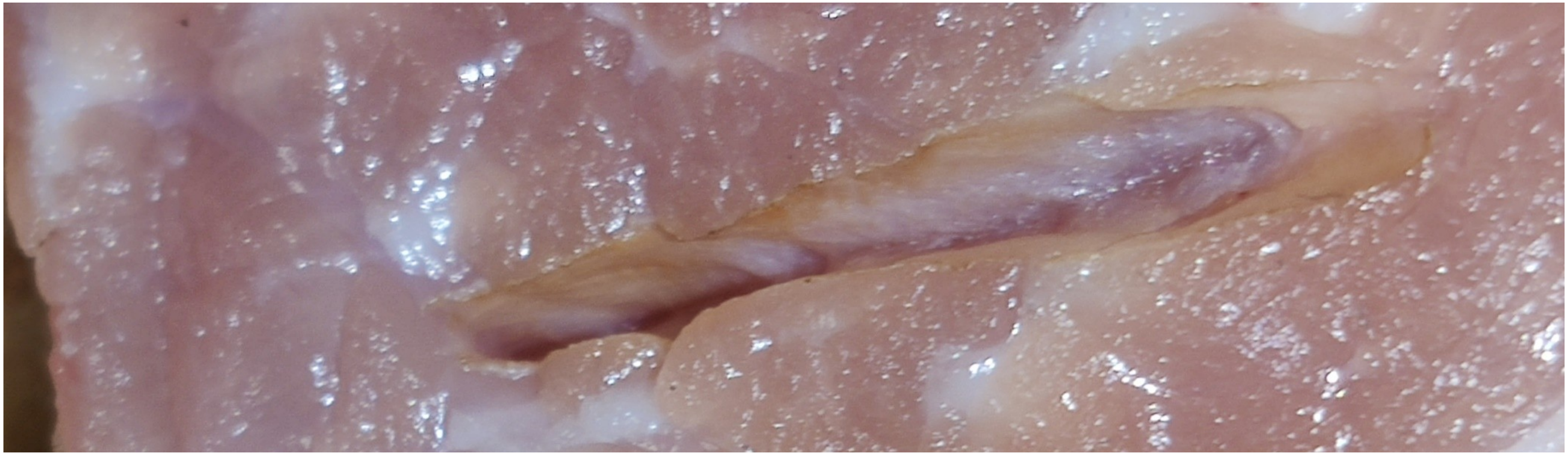

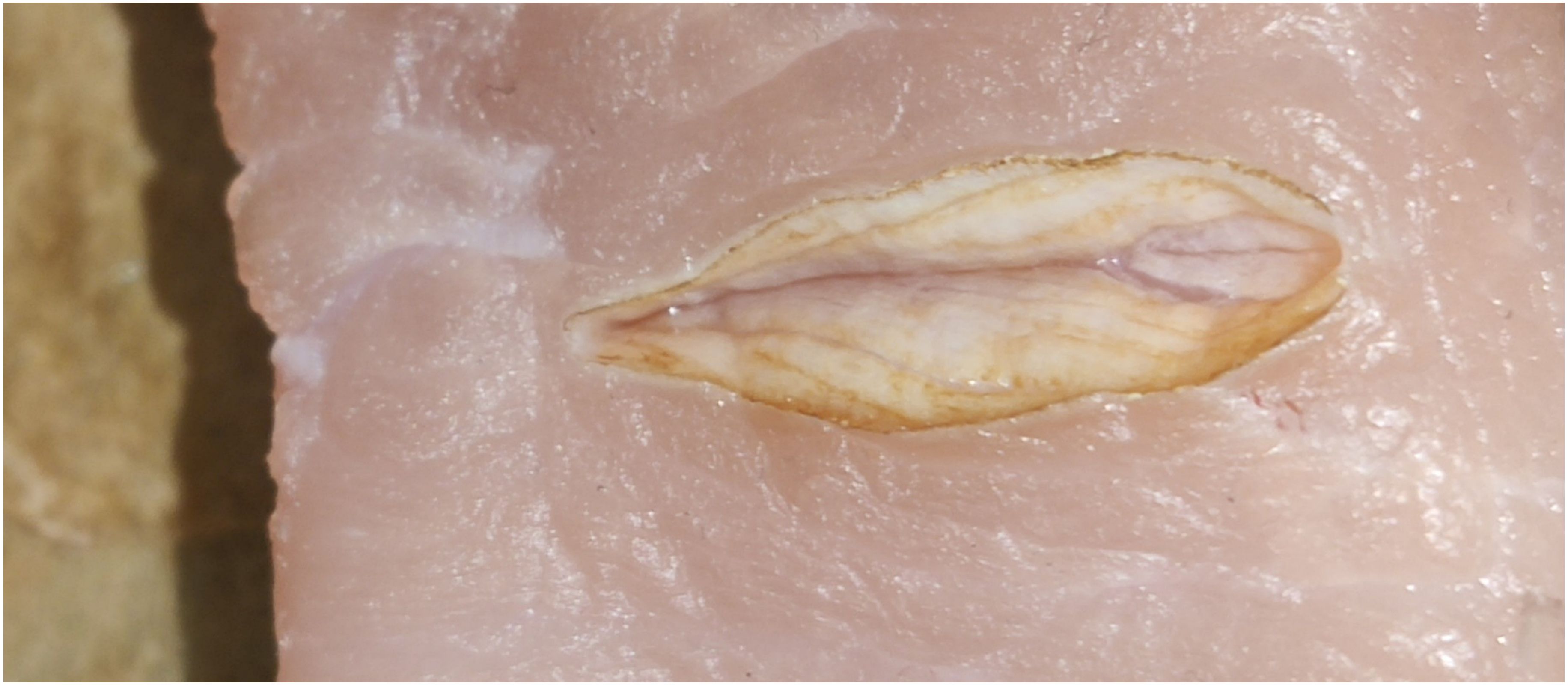

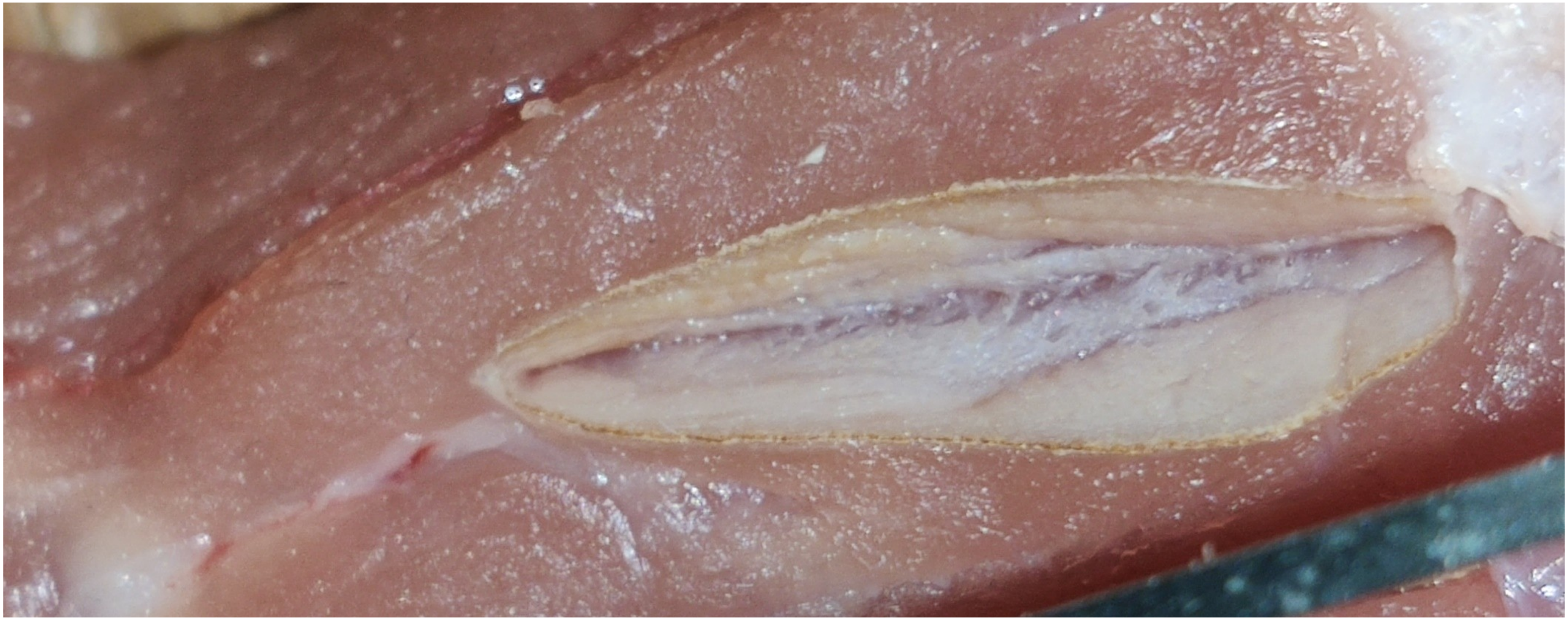

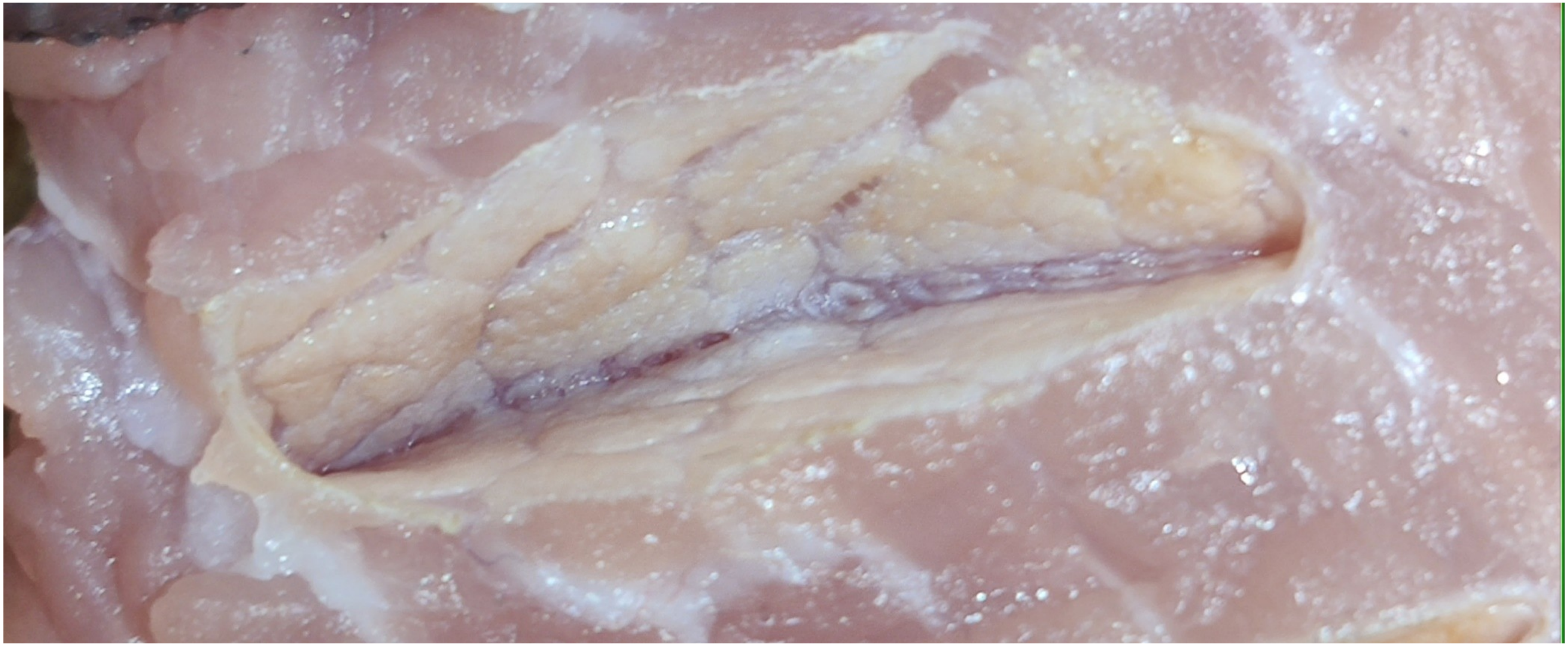

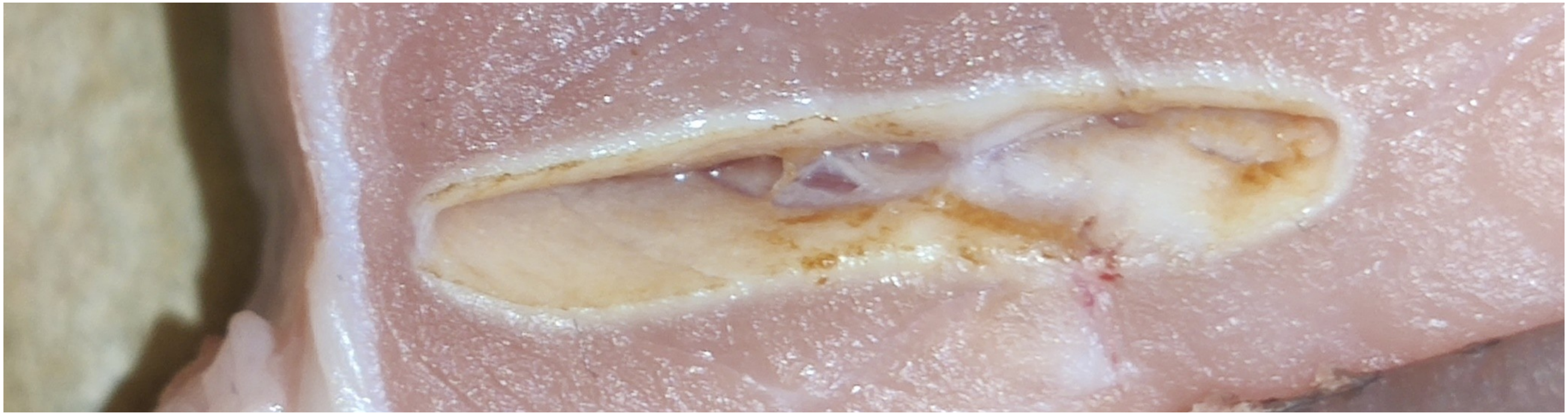

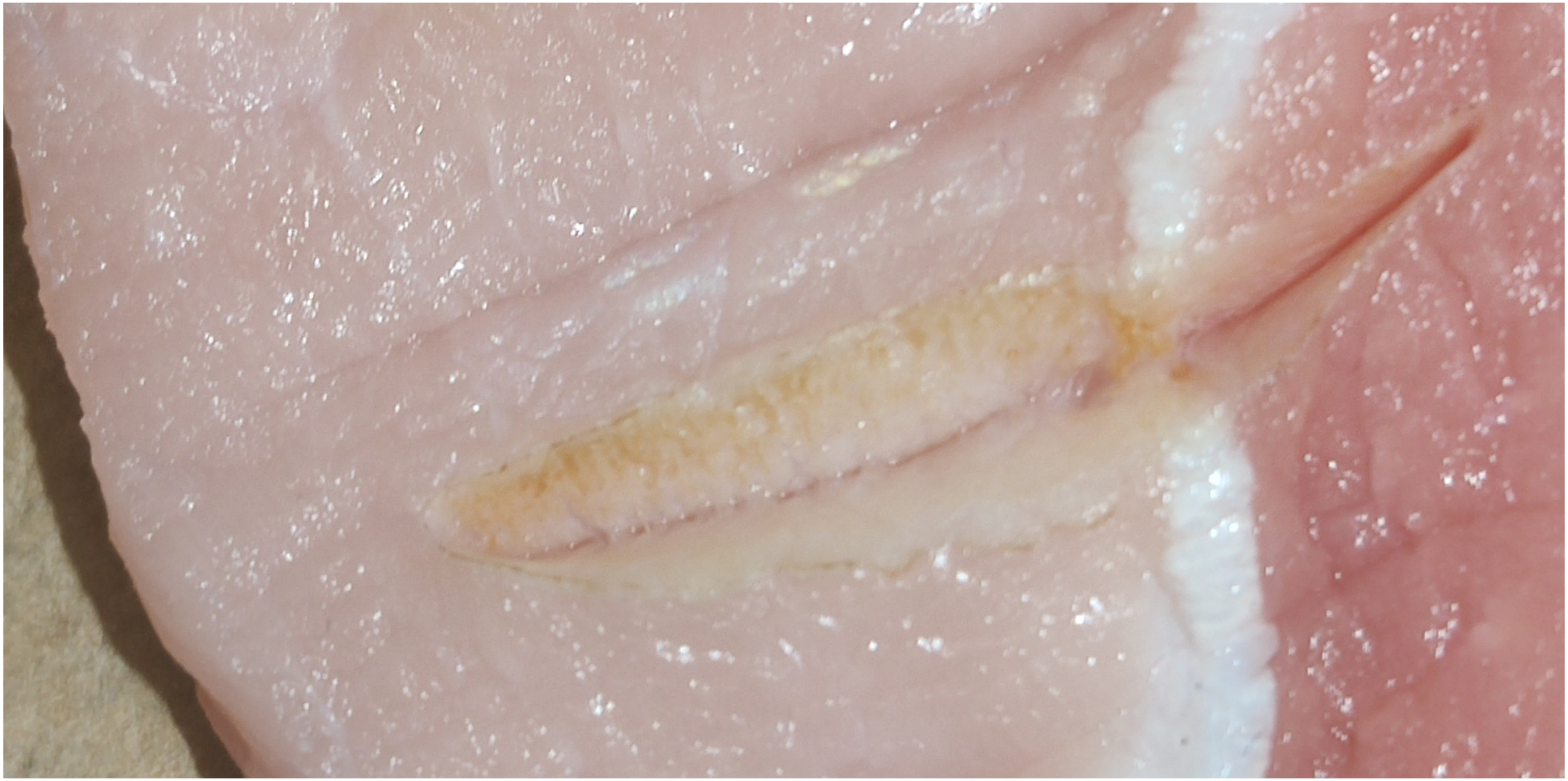

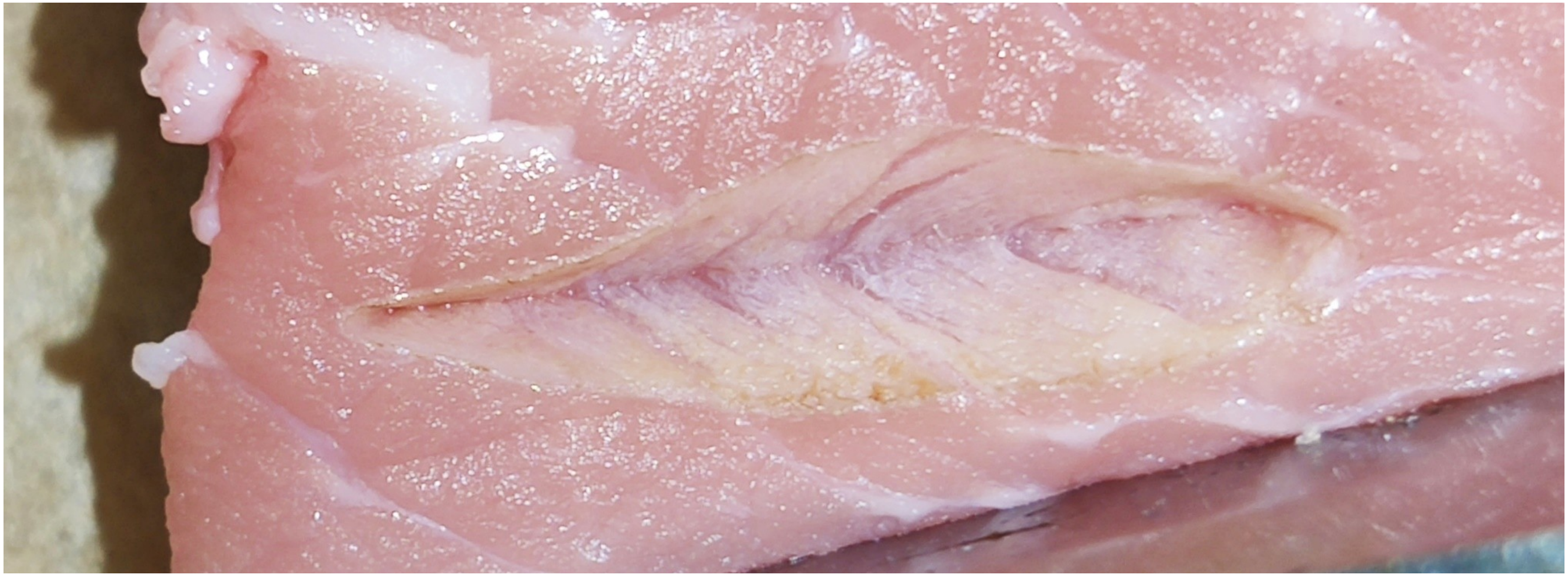

Supplement: Supplementary file 1 — Supplementary Information. [file 41598_2022_18969_MOESM1_ESM.pdf]
